# Glycosylated extracellular matrix drives immune suppression by modulating macrophage-T cell crosstalk in triple-negative breast cancer

**DOI:** 10.1038/s41467-026-73467-5

**Published:** 2026-06-16

**Authors:** Ludovica Tarantola, Eleanor J. Tyler, Ying Liu, Eleni Maniati, Katie A. Thornton, Celia Martín-Otal, Daire Hanna, Rithu Kumar, Valentine Gauthier, Priyanka Hirani, Marcos Burger Ramos, Nick J. Roth, Julie Bragg, Eliott H. Puttock, Jacqueline McDermott, Vinothini Rajeeve, Pedro Cutillas, Oscar Maiques, Annelise Soulier, Pedro Correa de Sampaio, Louise J. Jones, David M. Davies, John Maher, Stuart M. Haslam, Heinz Läubli, Oliver M. T. Pearce

**Affiliations:** 1 Queen Mary University of London, Barts Cancer Institute, John Vane Science Centre, London, UK; 2 Neobe Therapeutics, Salisbury House, Station Road, Cambridge, UK; 3 Leucid Bio, Guy’s Hospital, Great Maze Pond, London, UK; 4 Department of Life Sciences, Imperial College London, London, UK; 5 Department of Biomedicine and Division of Medical Oncology, University Hospital Basel, Basel, Switzerland

**Keywords:** Breast cancer, Glycobiology, Tumour immunology

## Abstract

The tumor extracellular matrix (ECM) is increasingly recognized as a key driver of immune suppression and therapy resistance in cancer. However, the specific ECM components and mechanisms that create this immunosuppressive environment remain poorly understood, hindering the development of new therapies. Here, we use comprehensive multi omics profiling of triple-negative breast cancer (TNBC), an aggressive and treatment-resistant subtype, to investigate this issue. We report that ECM immunomodulation in TNBC is mediated by post-translational glycan modifications on ECM proteins. Using decellularized human TNBC samples, we show that targeted enzymatic removal of these ECM glycans modifies the tumor immune microenvironment. This modification reprograms tumor-associated myeloid cells toward an immunomodulatory phenotype and improves infiltration of T cells. Notably, ECM desialylation alters selectin and selectin-ligand programs on T cells, consistent with improved trafficking and intratumoral access. In parallel, macrophage–T cell interactions are reshaped, leading to reduced T cell exhaustion. Our findings identify ECM glycan modifications as critical regulators of the innate and adaptive TNBC immune microenvironment. They suggest that targeting ECM glycosylation could offer potential strategies to boost anti-tumor immunity in this aggressive breast cancer subtype.

## Abstract (web summary)

The extracellular matrix provides structure to tumor tissue and can act as a barrier to anti-tumor immunity. Here, the authors demonstrate that enzymatic modification of matrix glycans shapes T cell-macrophage interactions and reduces immunosuppression within triple-negative breast cancer tumors.

## Introduction

Triple-negative breast cancer (TNBC) is an aggressive and therapeutically challenging subtype of breast cancer. Defined by the absence of estrogen receptor (ER), progesterone receptor (PR), and human epidermal growth factor receptor 2 (HER2) expression, TNBC accounts for approximately 10–20% of all breast cancers^1^. Its clinical management is hindered by the lack of targeted therapies, leaving chemotherapy as the primary treatment option. Despite initial responsiveness, TNBC is associated with high rates of recurrence and metastasis, emphasizing the urgent need for innovative therapeutic strategies^2^.

Immune checkpoint inhibitors (ICIs) have emerged as a promising therapeutic approach for TNBC. By targeting inhibitory pathways such as PD-1/PD-L1 and CTLA-4, ICIs aim to reinvigorate exhausted T cells and restore anti-tumor immunity. The approval of atezolizumab or pembrolizumab in combination with chemotherapy for PD-L1-positive metastatic TNBC represents a significant advance^3,4^. However, the clinical benefits of ICIs are limited to a subset of patients, underscoring the need to address resistance mechanisms such as immune exclusion. Among these emerging immunotherapeutic strategies, chimeric antigen receptor T-cell (CAR-T) therapy has shown remarkable efficacy in hematological malignancies but faces significant challenges in solid tumors like TNBC^5^.

A hallmark of TNBC is its complex and dynamic tumor microenvironment (TME), comprising cellular and non-cellular components, including immune cells, fibroblasts, vasculature, and the extracellular matrix (ECM)^6^. The ECM is a three-dimensional network of collagens, glycoproteins, and proteoglycans and polysaccharides that provide structural support and regulates cell signaling, migration and immune surveillance^7,8^. The ECM often makes up the majority of the tumor by volume, however it is only recently that the functional roles of ECM in driving tumor progression, immune suppression and therapeutic resistance are beginning to be untangled. In TNBC, the ECM frequently exhibits significant desmoplasia, resulting in increased stiffness, and altered composition of the ECM, all of which may contribute to reduced therapeutic efficacy^9^.

Recent studies highlight the ECM as a critical immunologic barrier in solid tumors, restricting cytotoxic immune cells to stromal regions and preventing effective tumor cell contact. Whilst the underlying mechanisms through which ECM exerts its immunological control remains poorly understood, limiting the potential for therapeutic intervention, recent work highlights ECM composition^10^, metabolism^11^, mechanosensing^12^, and structural organization^13^ in regulating immune cell activity and location within the TME. For example, our previous analysis of primary TNBC tissues identified the proteoglycan versican (VCAN) as a key regulator of T cell trafficking within TNBC^14^. We found VCAN can have opposing roles either supporting or inhibiting T cell trafficking that was dependent on the type of post-translational glycosaminoglycan (GAG) modifications on its protein backbone. This work indicated that the immunological function of ECM may be controlled via the display of post-translational glycans. It also indicated that a relatively tiny change in ECM glycan structure is enough to create different, in this case opposite, immune environments and suggest that targeting specific ECM post-translational modifications could overcome immune exclusion and improve anti-tumor immunity and immunotherapy response.

Aberrant glycosylation in cancer can promote immune evasion by engaging with immunomodulatory, often inhibitory, Siglec receptors expressed on immune cells^15^ and altering immunosuppressive immune cell infiltration^16^. Similarly, altered glycosylation in the ECM influences interactions between glycan-binding receptors, such as selectins, and their ligands on immune cells, affecting T cell responses^17^. In TNBC, we hypothesize that the glycosylation on tumor ECM exacerbates immune exclusion, through an orchestrated mechanism involving the direct education of immune cell activity, as we recently found for a population of tumor-associated macrophages^18^, and changing the crosstalk between immune cell types. This phenomenon could significantly limit the success of immunotherapies, which rely on robust immune cell activation and tumor infiltration, and therefore by disrupting ECM glycosylation patterns may improve immune cell infiltration and function within tumors. To further explore these questions around ECM-glycosylation in the regulation of tumor immunity, we begin with a multilayered analysis of the tumor microenvironment of human TNBC defining the diseased ECM and the transcript, translational and post-translational glycan composition finding significant upregulations in ECM glycoproteins and glycans that associate with both T cell and macrophage infiltrate, and the overall immune phenotype of the tumor. To test these associations, we then built a decellularized human tumor model of TNBC that we first validated using a combination of characterization data and reproducing well defined tumor cell phenotypes. Using this model we found ECM controls T-cell movement and macrophage phenotype, and in both cases these control mechanisms could be altered or overcome by deleting specific glycans on ECM proteins. Finally, we found that ECM glycosylation not only directly affects immune cell activity, but also mediates the crosstalk of macrophages with T cells. These data indicate that it is possible to make significant changes to immune cell activity through relatively small alterations to the tumor ECM, offering new opportunities for ECM-centric therapies that support immune cell mediated tumor destruction.

## Results

### Multi-layered characterization of TNBC reveals dysregulation of the ECM

**Fig. 1:**
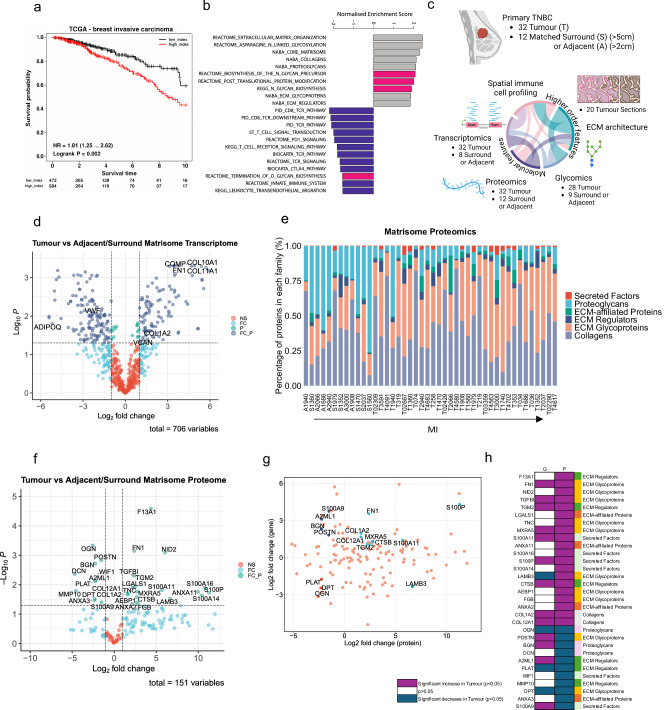
Multi-layered characterization of TNBC revealed a dysregulated extracellular matrix. **a** Kaplan–Meier survival curve for matrix index (MI) high (584 patients) vs MI low (472 patients) from TCGA invasive breast cancer dataset. Survival differences were assessed using a two-sided log-rank test. Hazard ratio (HR) and 95% confidence interval (CI) were estimated using a Cox proportional hazards model. No adjustments for multiple comparisons were applied. **b** Differentially expressed genes between MI high and MI low patient samples are enriched for genes upregulated in ECM remodeling (gray bars) and post-translational glycosylation (pink bars) and genes downregulated in immunity (blue bars). **c** Schematic of multi-layered matched bioinformatics analysis in TNBC. Created in BioRender. Tyler, E. (2026) https://BioRender.com/97bax21. **d** Volcano plot of differentially regulated matrisome genes between matched tumor vs ‘healthy’ adjacent/surround tissue. Differential expression was assessed using a two-sided Wald test. P values were adjusted for multiple comparisons using the Benjamini–Hochberg false discovery rate (FDR) method. Dashed lines indicate the thresholds for significance. Points are colored as non-significant (NS), fold change only (FC), P value only (P), or both (FC_P). **e** Stacked barchart of proteomic matrisome compartment proportions for all tumor and adjacent/surround tissues ranked by MI. **f** Volcano plot of differentially regulated matrisome proteins between matched tumor vs ‘healthy’ adjacent/surround tissue. Two-sided Student’s *t* test. P values were adjusted for multiple comparisons using the Benjamini–Hochberg false discovery rate (FDR) method. Dashed lines indicate the thresholds for significance. **g** Scatterplot of matrisome molecules significantly differentially expressed between tumor and matched ‘healthy’ adjacent/surround at protein and gene levels. Molecules significantly downregulated in tumor at gene and protein level (Red). Molecules significantly upregulated in tumor at gene and protein level (Light Blue). Molecules significantly downregulated in tumor at gene level, but significantly upregulated in tumor at protein level (Green). Molecules significantly upregulated in tumor at gene level, but significantly downregulated in tumor at protein level (Dark Blue). **h** Overlap of molecules significantly upregulated (Purple) or downregulated (Blue) in tumor at the gene (G) and protein (P) levels, and their annotated matrisome compartment.

Previously, we identified a tumor-associated ECM signature, termed the matrix index (MI), which was an indicator of poor prognosis in high-grade serous ovarian cancer (HGSOC) and associated with increased immunosuppressive immune cell types and prognostic value in 13 major cancer types, most significantly in TNBC^10^. Here, we further explored the dysregulated pathways in TNBC tissues with a high MI and poor prognosis (Fig. 1a, Supplementary Fig. 1a), discovering that these tissues were enriched for genes associated with N-linked glycosylation and suppression of adaptive immunity (Fig. 1b, Supplementary Fig. 1b), supportive of the hypothesis that glycans displayed on ECM proteins confer the tumor ECM with anti-tumor immunity in TNBC.

We analyzed primary tumor tissues from 32 patients with TNBC and matched histopathologically normal adjacent/surround tissues (‘adjacent’) from 12 patients using RNA-seq (Supplementary Data 1, Supplementary Fig. 1c–e), ECM-enriched proteomics (Supplementary Data 2), N-linked glycomics (Supplementary Data 3), and spatial immune cell profiling (Fig. 1c). Changes in the ECM between adjacent tissue and tumor tissue were assessed at the gene (Fig. 1d) and protein level (Fig. 1f). We identified 245 ECM genes (Supplementary Data 4) and 31 ECM proteins (Supplementary Data 5) that were significantly dysregulated, with 16 overlapping between the two datasets (Fig. 1g). Further categorization of ECM components into core matrisome proteins (proteoglycans, ECM glycoproteins, and collagens) and matrisome-associated proteins (secreted factors, ECM-affiliated proteins, and ECM regulators) revealed an expansion of glycosylated ECM proteins (‘ECM glycoproteins’) in tumor tissue compared to adjacent tissue, which positively correlated with MI (Fig. 1e). Among the dysregulated molecules, most overlapping between gene and protein datasets were classified as ECM glycoproteins (Fig. 1h), including fibronectin (FN1) and matrix remodeling-associated 5 (MXRA5), which we previously associated with an immunosuppressive macrophage phenotype in HGSOC^18^. These analyses describe the changes to ECM composition in TNBC in which ECM glycoproteins are prominent features of the diseased ECM.

### Aberrant ECM post-translational modifications are strongly associated with immune cell infiltration

**Fig. 2:**
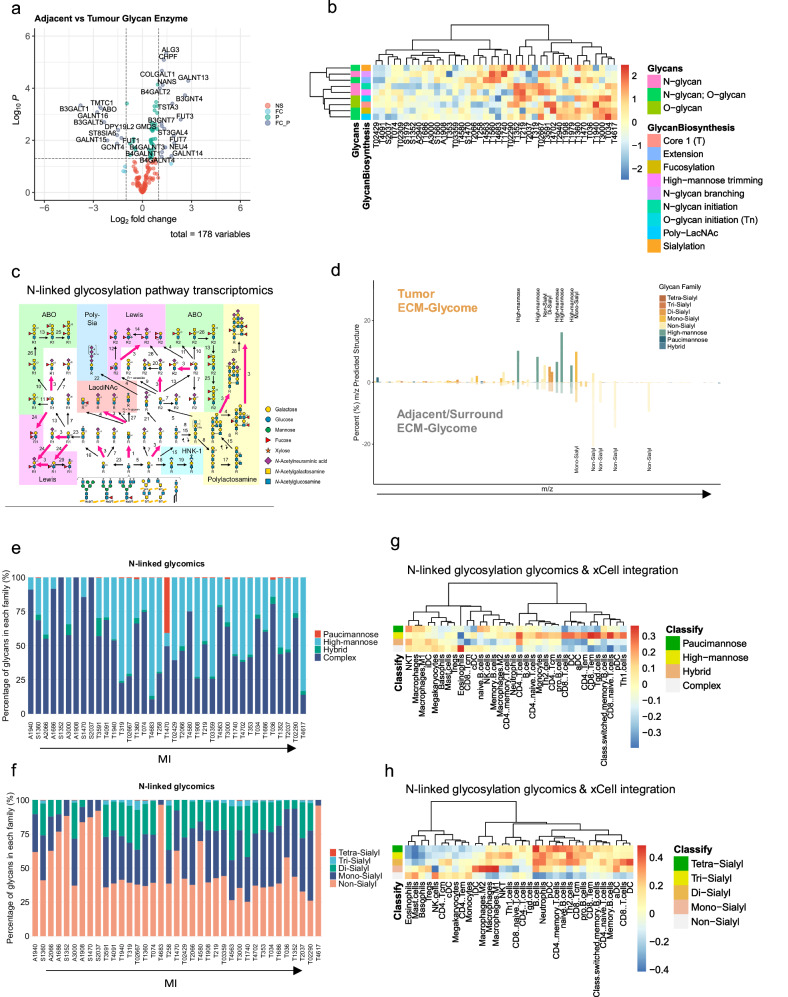
Multi-layered characterization of TNBC revealed altered ECM post-translational glycans which significantly associated with immune cell infiltration. **a** Volcano plot of differentially expressed (DE) glycosylation pathway genes between matched tumor and ‘healthy’ adjacent/surround tissues. Genelist from Huang et al., 2021. *N* = 8 per group. DE assessed using a two-sided Wald test. P values adjusted for multiple comparisons using the Benjamini–Hochberg false discovery rate (FDR) method. Dashed lines indicate the thresholds for significance. **b** Heatmap of row normalized values summing the gene expression values for genes annotated in each Glycan Biosynthesis family (Supp Data). Ordered by ward.D2 unsupervised clustering. *N* = 9 adjacent/surround tissues, *N* = 32 tumor tissues. **c** Glycomaple comparison of glycosylation pathways between tumor and adjacent tissues. X = (Tumor TPM + 1)/(Adjacent TPM + 1) Pink arrows x >= 1.5. Black arrows no change. Green arrows x < 0.5. *N* = 8 for each group. **d** All ECM N-linked glycans mass (*m/z)* observed in the MALDI-MS spectra from matched tumor and ‘healthy’ adjacent/surround tissues. Mirrored barchart plotting percent (%) intensity for each N-linked glycan detected. Tumor above x axis, adjacent/surround below x axis. Bars colored by glycan family. *N* = 9 for each group. **e** ECM N-linked glycans assigned to four main groups (Complex, dark blue; Hybrid, green; High-mannose, light blue; Paucimannose, red). Stacked barchart displaying the proportion of each family in the N-glycome for all adjacent/surround and tumor samples ranked by MI. *N* = 9 adjacent/surround tissues, *N* = 28 tumor tissues. **f** ECM N-linked complex glycans sorted into five groups (Non-Sialyl, peach; Mono-Sialyl, dark blue; Di-Sialyl, green; Tri-Sialyl, light blue; Tetra-sialyl, red). Stacked barchart displaying the proportion of each family in the N-glycome ranked by MI. *N* = 9 adjacent/surround tissues, *N* = 28 tumor tissues. **g** Heatmap of main N-linked glycan families (Complex, Hybrid, High-mannose, Paucimannose) and h, Heatmap of complex N-linked glycan families (Non-Sialyl, Mono-Sialyl, Di-Sialyl, Tri-Sialyl, Tetra-Sialyl) and their associations with immune cells from xCell analysis in tumor tissues. Spearman’s r values with two-sided alternative hypothesis testing representing correlation between immune abundances and N-linked glycan family. Ordered by unsupervised clustering. *N* = 28 tumor tissues.

Building on the finding that ECM glycoproteins were a prominent feature of the tumor ECM, we wanted to further define the glycans involved. We first examined changes in glycosylation biosynthesis pathways at the transcript level between adjacent tissues and tumor tissues from TNBC patients, revealing significant dysregulation of glycosylation biosynthesis genes (Fig. 2a). Annotating these genes into glycan biosynthesis families showed that tumor and adjacent tissues separated into two distinct clusters, with tumor tissues displaying upregulation of N-linked and O-linked glycan biosynthesis processes, including fucosylation, poly-LacNAc synthesis, and sialylation (Fig. 2b). GlycoMaple, a validated tool for estimating glycan structures based on gene expression, confirmed these findings, predicting a significant increase in N-linked glycan sialylation, fucosylation, and poly-LacNAc structures in tumor tissue (Fig. 2c). We confirmed the presence of these N-glycan structures on the ECM by fractionating patient tissues into cellular, membrane and ECM compartments and performing N-linked glycomics on the ECM-enriched fraction. This confirmed an increase in sialyl lewis (Supplementary Fig. 2a–c), fucosylation (Supplementary Fig. 2d–f), poly-LacNAc (Supplementary Fig. 2g–i), and sialylation (Fig. 2d–f) on the ECM, as predicted at the transcriptomic level. Additionally, we observed a shift towards high-mannose N-glycans of lower complexity in tumor tissues, consistent with previous literature (Fig. 2d, e)^19^. Similar shifts were observed in the cell membrane fraction (Supplementary Fig. 3).

Analysis of O-linked glycans on the ECM (Supplementary Fig. 4) and cell membrane (Supplementary Fig. 5) showed increased Sialyl-Tn antigen (STn) (Supplementary Fig. 4c) and sialylated glycans (Supplementary Fig. 4e, f). However, O-glycan detection was limited in our samples (Supplementary Fig. 4a). Using lectin immunofluorescence staining, we confirmed significant increases in sialylation, poly-LacNAc, high-mannose N-glycans, and O-glycans on the ECM in tumor tissue compared to adjacent tissue (Supplementary Fig. 6). Applying xCell, an in silico tool for dissecting the tumor immune microenvironment, to our RNA-seq data patient-matched to the N-linked glycan data (Supplementary Fig. 7), we found moderate positive correlations between high-mannose and hypersialylated N-linked glycans (di-, tri-, and tetra-sialylated) with T cell, dendritic cell, and B cell signatures, suggesting a relationship between the ECM glycome and adaptive immune infiltrates in the TME (Fig. 2g, h).

### TNBC TME is enriched for an immune-excluded phenotype, with N-linked glycosylation and sialylation associated with T cell localization

**Fig. 3:**
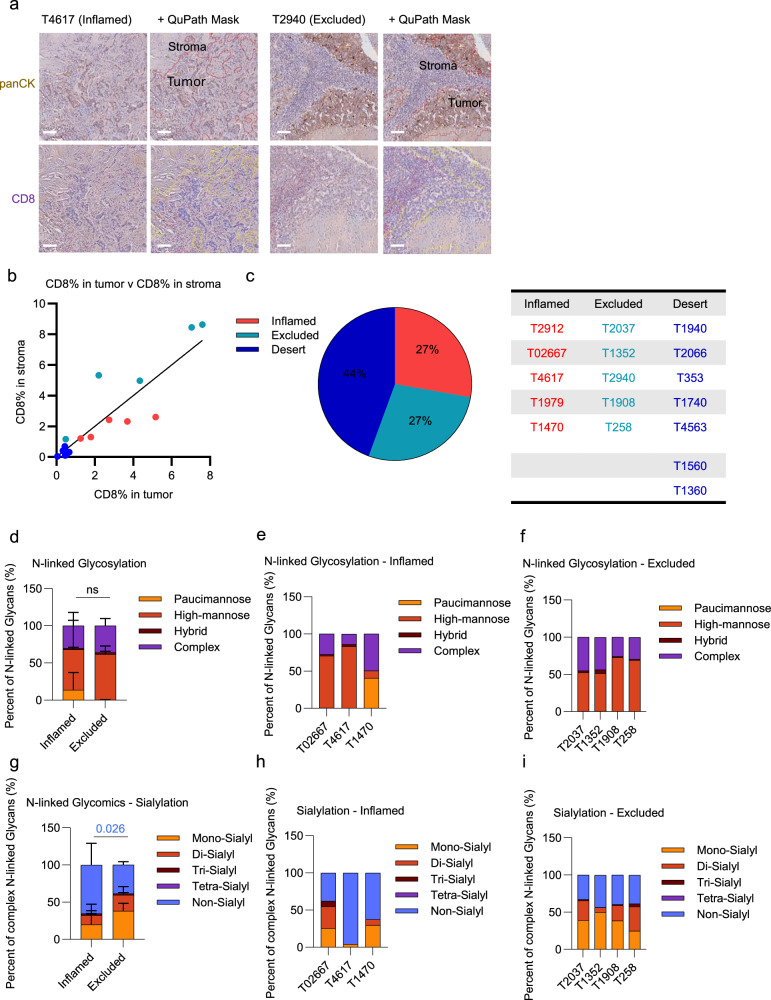
TNBC TME is enriched for an immune excluded phenotype, with N-linked glycosylation and sialylation associated with T-cell localization. **a** Representative images of TNBC patient tissue stained with anti-panCK (DAB) for tumor cells and anti-CD8 (VIP) for CD8+ cytotoxic T cells. Scale bar = 100 µm. Tumor and stroma annotations and CD8 T cell detections performed using QuPath as illustrated. Intensity features for annotations calculated using QuPath. *N* = 18 tissues. **b** Pearson’s r value for correlative analysis of CD8% in the stroma compared to CD8% in the tumor. Red = inflamed, green = excluded, blue = desert. *N* = 17 tissues. **c** Pie chart showing distribution of phenotypes for 17 tissues. ECM N-linked glycans assigned to four main groups (Complex, purple; Hybrid, dark red; High-mannose, red; Paucimannose, orange). Stacked barchart displaying the proportion of each family in the N-glycome for **d**, mean inflamed and excluded samples and individual **e**, inflamed or **f**, excluded samples. **g**–**i** ECM N-linked complex glycans assigned to four main groups (Non-Sialyl, blue; Mono-Sialyl, orange; Di-Sialyl, red; Tri-Sialyl, dark red; Tetra-Sialyl,, purple). Stacked barchart displaying the proportion of each family in the N-glycome for **g**, mean inflamed and excluded samples and individual **h**, inflamed or **i**, excluded samples. **d**–**i** Mean with SD. Two-way Repeated Measures ANOVA with Šídák’s multiple comparisons test, *N* = 4 excluded tissues, *N* = 3 inflamed tissues.

Bulk RNA-seq and ECM proteomics lack spatial resolution, so we performed spatial immune cell profiling of CD8 + T cells using immunohistochemistry on 20 FFPE patient tissues matched to our ECM analysis (Fig. 3a). We stratified tissues into three categories as we previously described^14^: 27% ‘inflamed’ tumors with CD8 + T cells infiltrating the tumor core, 44% ‘desert’ tumors with low overall CD8 + T cell infiltration, and 27% ‘excluded’ tumors with immune cells trapped outside the tumor nest (Fig. 3b-c). We integrated this analysis with the patient-matched N-linked glycomics data by stratifying the dataset by inflamed and excluded tumor phenotypes, which revealed that excluded tissues exhibit significantly elevated sialylation compared to inflamed tissues (Fig. 3d–i). Stratification of RNA-seq or proteomics data revealed no significant differences in matrisome genes or proteins at either the transcript or protein levels between inflamed and excluded phenotypes (Supplementary Fig. 8a, b).

### Decellularized TNBC tissue exhibits an immune-excluded TME, reversible by modifying ECM glycans

**Fig. 4:**
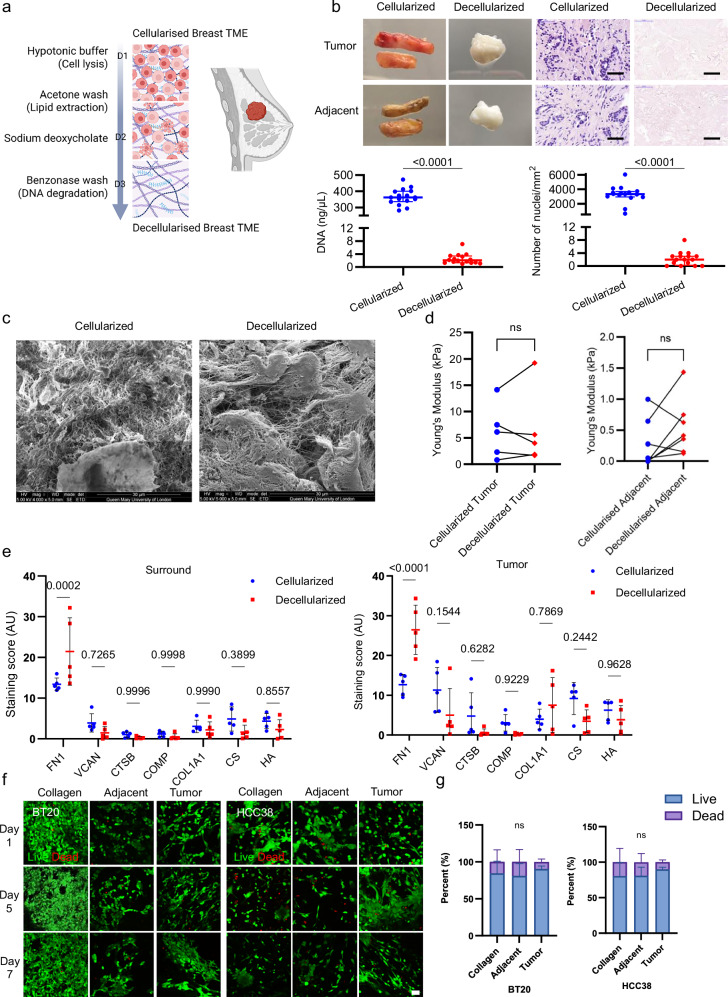
Development of a TNBC decellularized tissue model to test novel therapeutics targeting dysregulated tumor TNBC ECM. **a** Schematic of the decellularization process. Created in BioRender. Tyler, E. (2026) https://BioRender.com/up6uhfi. **b** Fresh tissue biopsies taken from TNBC patients. H&E staining of section TNBC samples. H&E, Scale bar = 50 µm. Nuclei and nuclei acid content in cellularized and decellularized tissue samples. Data are presented as median values +/- interquartile range (IQR). Two-tailed unpaired t test. *N* = 10 each. **c** Representative scanning electron microscope (SEM) images for cellularized and decellularized tumor tissue samples. Scale bar = 30 µm. **d** Tissue stiffness of cellularized and decellularized tumor and adjacent tissues using Instron 3342 with a 10 N load cell. Two-tail paired t test. *N* = 5 each. **e** IHC staining analysis using Definiens® digital image software for matrix molecules in cellularized and decellularized samples. Error bars represent mean ± standard deviation (SD). Two-way ANOVA with Šídák’s multiple comparisons test. *N* = 5 each. **f** Representative live (green)/dead (red) immunofluorescence images (IF) from decellularized tissues recellularized with BT20 and HCC38 TNBC cell lines. Scale bar = 100 µm. **g** Image analysis of Day 7 BT20 and HCC38 cell viability between cells seeded on collagen-coated plastic, adjacent tissue, and tumor tissue. Data are presented as mean values +/- standard deviation (SD). Two-way ANOVA with Sidak’s multiple comparisons test. *N* = 3 collagens, *N* = 3 adjacent tissues, *N* = 3 tumor tissues.

To investigate the influence of matrix glycosylation on the TME in vitro, we developed a decellularized tissue model of tumor ECM for TNBC, based on our previously published protocol^18^. Briefly, tissues were sectioned into 350 µm slices using a vibratome, followed by a series of washes to lyse cells, extract lipids, and degrade residual nucleic acids (Fig. 4a). Decellularization was confirmed by assessing cell removal (Fig. 4b), after which ECM architecture (Fig. 4c and Supplementary Fig. 10), mechanical properties (Fig. 4d), glycosylation patterns (Supplementary Fig. 10), and composition (Fig. 4e and Supplementary Fig.9) were found to remain intact post-processing.

To validate decellularized tissues for cell culture and testing the immune function of ECM glycosylation, we first evaluated cancer cell viability in the model. TNBC cell lines BT20 and HCC38 were cultured on decellularized matrices derived from adjacent or tumor tissues for 7 days. Notably, TNBC cells maintained high viability throughout the culture period, comparable to cells grown on collagen substrate (Fig. 4f, g).

**Fig. 5:**
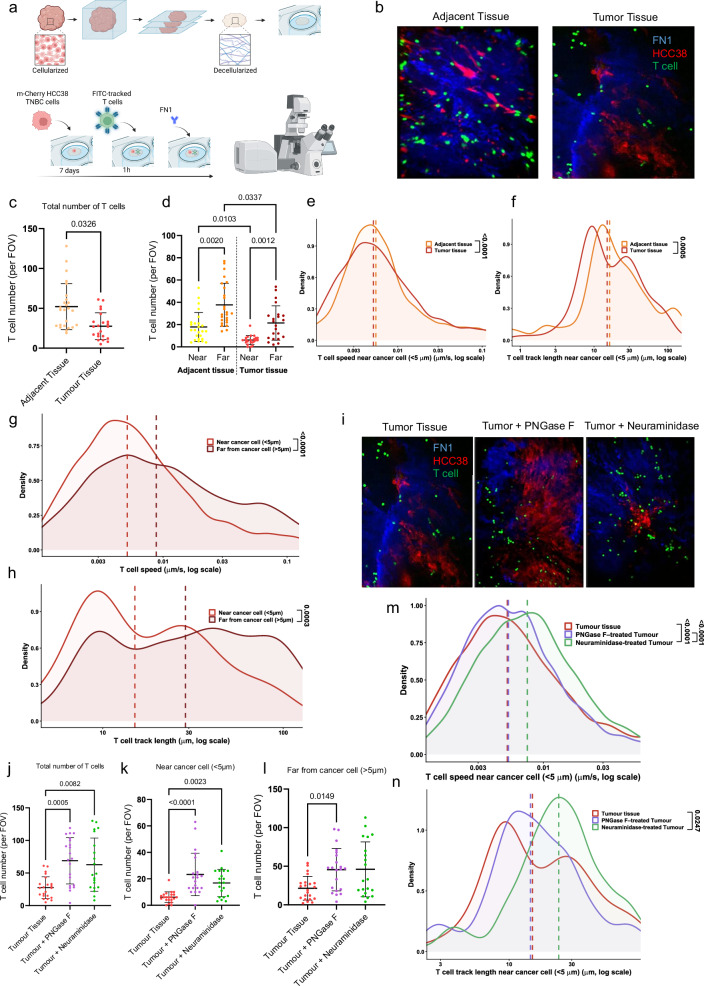
Decellularized TNBC tissue maintains an immune excluded TME that is converted to an immune inflamed phenotype through glycan modification. **a** Schematic of a fluorescence-based live confocal imaging assay using decellularized tissue. Created in BioRender (Tarantola, L., 2026) https://BioRender.com/de0fus5. **b** Representative images of adjacent and tumor decellularized tissue recellularized with mCherry HCC38 TNBC cells (red) and T cells (green) with ECM stained with anti-FN1-AF405 (blue). Scale bar = 100 µm. Total T cell number per field of view (FOV) (**c**) and T cell number near (<5 µm) or far (> 5 µm) from cancer cells (**d**) in tumor and adjacent tissues. *N* = 3 donors/group, 5–10 FOV/tissue. Error bars represent mean ± SD. Density distributions of T cell speed (**e**) and track length (**f**) near (<5 µm) cancer cells on tumor (red) and adjacent (orange) tissue. *N* = 3 tumor and *N* = 3 adjacent tissues, 2 technical replicates each. Density distributions of T cell speed (**g**) and track length (**h**) near (<5 µm) or far (>5 µm) from cancer cells in tumor tissue. *N* = 3 tumor tissues, 2 technical replicates each. **c**–**h** Dashed lines indicate medians. Data are presented in log scale. Kruskal–Wallis test with Dunn’s multiple comparisons test (two-sided, adjusted for multiple comparisons). **i** Representative images of tumor control tissue and tumor tissue treated with PNGase F or neuraminidase and recellularized with mCherry HCC38 TNBC cells (red) and T cells (green), with ECM stained with anti-FN1-AF405 (blue). Scale bar = 100 µm. Total T cell number per FOV (**j**), T cells near cancer cells (<5 µm) (**k**), and far from cancer cells (>5 µm) (**l**). *N* = 3 donors, 6–10 FOV/tissue. Error bars represent mean ± SD. Density distributions of T cell speed (**m**) and track length (**n**) near (<5 µm) cancer cells on tumor (red), PNGase F-treated (violet), or neuraminidase-treated (green) tissue. Dashed lines indicate medians. Data are presented in log scale. *N* = 3 tumor tissues, 2 technical replicates each. **j**–**n** Kruskal–Wallis test with Dunn’s multiple comparisons test (two-sided, adjusted for multiple comparisons).

Having established the model was suitable for exploring the function of ECM glycosylation, we now turned our attention to exploring immune cell activity. We previously established a relationship between ECM glycosylation and T cell infiltration in the multi-layered ‘omics analysis (Figs. 2f–h, 3), so we asked whether ECM glycosylation was directly involved in CD8 + T cell infiltration using the decellularized tissue model that was first recellularized with TNBC cells as described above. We developed a live-cell imaging assay to monitor the movement and localization of primary T cells or TNBC-targeting CAR T-cells within the ECM (Fig. 5a, Supplementary Fig. 11). We used CAR T-cells as a surrogate for a natural tumor reactive T cell population. Tissues recellularized with HCC38 were cultured with primary T cells isolated from healthy donor peripheral blood mononuclear cells (PBMCs), whereas tissues recellularized with BT20 were cultured with CAR T-cells. First, we observed that CAR T-cell infiltration was minimal in decellularized tissues which were not recellularized with TNBC cells (Supplementary Fig. 12a). In these tissues, CAR T-cells exhibited slower motility compared to recellularized tissues (Supplementary Fig. 12b), indicating that the presence of cancer cells stimulated CAR T-cell infiltration. Comparing primary T cells or CAR T-cell infiltration between tumor-derived ECM and adjacent tissue, we found that both T cell and CAR T-cells infiltration was significantly reduced in tumor-derived ECM (Fig. 5b, c, Supplementary Fig. 13b, c). Furthermore, fewer T cells or CAR T-cells penetrated the tumor nest (≤5 µm from a cancer cell) in tumor ECM compared to adjacent tissue (Fig. 5d, Supplementary Fig. 13d and Supplementary Fig.14), resembling an immune-excluded phenotype. In both tumor and adjacent tissues, T cells or CAR T-cells preferentially accumulated in the stroma, consistent with the behavior of resident CD8 + T cells documented in real-time microscopy of fresh sections of human ovarian and lung tumors (Fig. 5d, Supplementary Fig. 13e, f)^20^.

Analysis of T cell motility revealed that T cells located near an HCC38 cancer cell (<5 µm) have an increased speed and displacement in adjacent tissues compared to tumor tissues (Fig. 5e, f). Moreover, within tumor ECM, T cells that are located near tumor islets (HCC38) exhibited a decreased speed and displacement compared to those in the surrounding ECM (Fig. 5g, h). These observations are consistent with previous reports showing that dense extracellular matrix architecture restricts T cell migration and limits their penetration and mobility into tumor islets^21^.

As we previously observed a relationship between T cells and N-glycosylation, in particular sialylation, in our multi omics’ analysis (Fig. 2h, Fig. 3), we wanted to test whether enzymatic removal of N-glycans or sialic acids significantly enhanced T cell infiltration. We confirmed firstly that the model was sensitive to enzymatic modifications by treating decellularized tissues with PNGase F to cleave N-glycans or with neuraminidase from *Clostridium perfringens* to remove terminal sialic acids on N- and O-linked glycans, which was confirmed by lectin staining (Supplementary Fig. 15).

Enzymatic removal of N-glycans or sialic acids significantly enhanced both T cell and CAR T-cells infiltration into tumor ECM (Fig. 5i, j, Supplementary Fig. 13i, j and Supplementary Fig.16) and increased the proportion of T cells or CAR T-cells penetrating the tumor nest (Fig. 5k, l, Supplementary Fig. 13k and Supplementary Fig. 16). Interestingly, T cells in PNGase F or neuraminidase-treated tissues exhibited greater speed and displacement, compared to untreated tumor tissue, when in proximity of a cancer cell (<5 µm), indicating that T cells are more easily able to move and reach the tumor islets through the ECM in enzyme-treated tissues (Fig. 5m, n).

However, the same analysis performed with BT20 co-cultured with CAR T-cells revealed that CAR T-cells located near tumor islets exhibited increased speed and displacement compared to those in the surrounding ECM (Supplementary Fig. 13g, h). This observation aligns with reports suggesting that tumor islets can serve as zones of enhanced T cell migration^20^, and also indicates cancer cell specific effects on T-cell migration are also active within the model. This phenomenon was not observed with BT20 and CAR T-cells co-culture in adjacent tissue, suggesting a specific role of tumor ECM in modulating CAR T-cells behavior (Supplementary Fig. 14d, e). Moreover, CAR T-cells in PNGase F or neuraminidase-treated tissues exhibited greater speed traveling through the ECM (>5 µm), indicating that CAR T-cells are more easily able to move through the ECM in enzyme-treated tissues (Supplementary Fig 13l, m).

Overall, our results demonstrate that enzymatic treatment of the tumor ECM can reverse the immune-excluded phenotype.

To explore the mechanism by which glycans modulate T cell infiltration, we focused on the sialic acid-Siglec axis, a known immune-inhibitory pathway that could be associated with the sialylation seen on tumor ECM. Analysis of Siglec receptor expression on CAR T-cells seeded on tumor tissue or neuraminidase-treated tumor tissue revealed approximately 5% of cytotoxic CD8 + T cells expressed Siglec-7 and 10% expressed Siglec-10, with no significant differences between conditions (Supplementary Figs. 17, 18). While Siglec-7 and Siglec-10 expression levels on T cells were unchanged following ECM desialylation, this does not exclude a role for Siglec-mediated signaling, as neuraminidase treatment primarily alters ligand availability rather than receptor expression. We also observed that the enzymatic treatments with PNGase F or neuraminidase altered tissue architecture, in particular increasing the arrangement of ECM fibers potentially enhancing CAR T-cells or T cell access to the tumor (Supplementary Figs. 19, 20). Taken together, these findings suggest that modifying ECM glycans can reprogram the immune-excluded TME into an immune-inflamed phenotype. Notably, removing glycans from the ECM leads to a reorganization of fiber architecture. Although this phenomenon was not explored further here, it may arise from altered glycan–glycan or glycan–protein interactions between ECM fibers, and/or from the release of ECM-associated factors that are normally retained through glycan-mediated binding. Such structural changes are likely to influence T-cell infiltration by jointly affecting glycan-dependent signaling, matrix organization, and the physical properties of the tissue.

### TNBC TME educates an immunomodulatory macrophage population mediated by ECM glycans

**Fig. 6:**
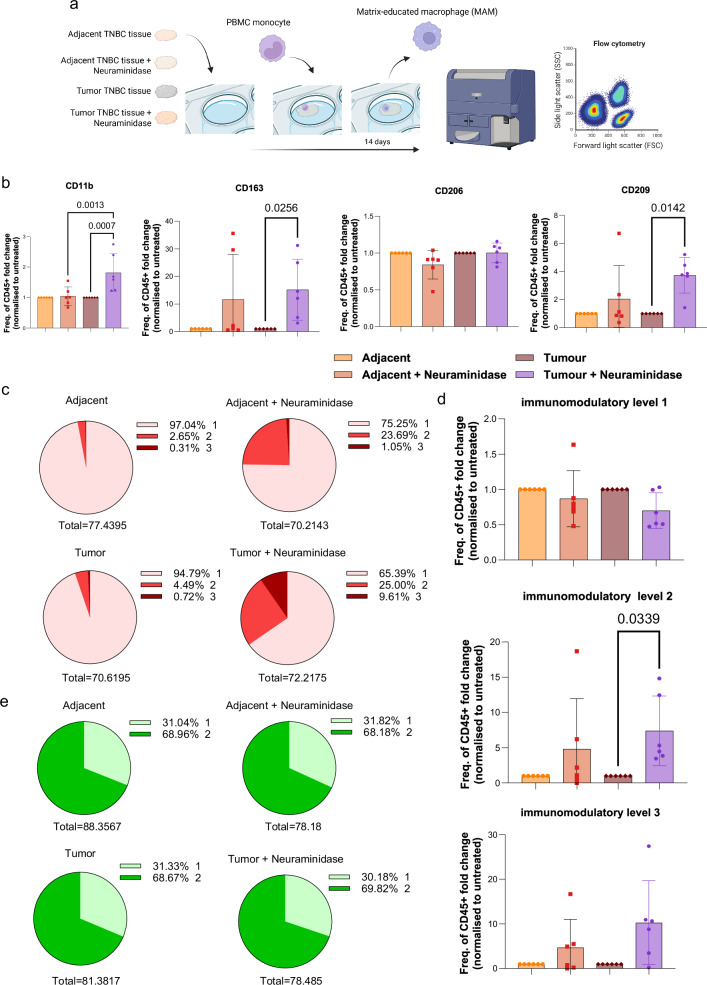
TNBC TME educates a tumor-associated macrophage population mediated by ECM-glycans. **a** Schematic of macrophage decellularized tissue culture. Monocytes from six separate blood donors were cultured for 14 days on tumor (*N* = 6) or adjacent (N = 6) decellularized tissues. Flow gating strategy provided in Supplementary Fig. 21. Created in BioRender. Tarantola, L. (2026) https://BioRender.com/wldk4w7. **b** Barchart of flow cytometry expression patterns of CD11b, CD163, CD206, CD209, shown as fold change normalized to untreated tissue of positive cells from the CD45+ population. Line at mean with standard deviation error bars. Mixed-effects analysis with Tukey’s multiple comparisons adjustment test (two-sided). *N* = 6 (*N* = 6 adjacent, *N* = 6 tumor, *N* = 6 blood cones). **c**–**e** Pie charts of combinatorial gating for **c** immuno-modulatory markers (CD163, CD206, CD209) or **e** antigen presentation markers (CD86, & HLA-DR). *N* = 3 (*N* = 3 adjacent, *N* = 6 tumor, *N* = 3 blood cones). **d** Barchart of combinatorial gating for activation markers (CD163, CD206, & CD209) shown as fold change normalized to untreated tissue of positive cells expressing one marker (activation level 1), two markers (activation level 2), or three markers (activation level 3) from the CD45+ population. Line at mean with standard deviation error bars. Mixed-effects analysis with Tukey’s multiple comparisons adjustment test (two-sided). *N* = 6 (N = 6 adjacent, *N* = 6 tumor, *N* = 3 blood cones).

Successful adaptive anti-tumor immune responses can heavily rely on support from innate immune cells, and in particular macrophages are often necessary for supporting T cell activity in suppressing tumors^22^. We previously observed that tumor ECM can directly educate the behavior of a tumor-associated macrophage phenotype in HGSOC^18^. To test whether this matrix-educated macrophage (MAM) was also a feature in TNBC, we utilized our decellularized tissue model. Monocytes isolated from healthy donor PBMCs were cultured on decellularized tumor and adjacent tissues, and macrophages were collected after 14 days (Fig. 6a, Supplementary Fig. 21). Macrophage phenotypic transitions were assessed through flow cytometric analysis of cell surface markers, including CD11b, CD209, CD206, CD163, CD86, HLA-DR, Siglec-1, and Siglec-9 (Supplementary Fig. 21b).

We observed significantly fewer CD11b+ MAMs educated on tumor tissue than adjacent tissue (Supplementary Fig. 22a). Given that CD11b has been implicated in regulating myeloid cell polarization and functional states^23^, this shift suggests that tumor ECM exposure may be associated with altered macrophage differentiation or immunomodulatory programs.

We previously observed a relationship between macrophages and sialylation in our multi omics’ analysis (Fig. 2h) and the transcriptomic profile of MAMs in HGSOC^18^ associates with a published dataset of hypersialylated MUC1 stimulated macrophages^24^. To test whether sialylation on ECM was educating the MAM phenotype in TNBC, we treated tumor ECM with neuraminidase which significantly increased the proportion of CD11b + , CD163+ and CD209+ macrophages (Fig. 6b). This effect was not observed on neuraminidase-treated adjacent ECM, implicating only sialic acids displayed on tumor ECM as modulators of macrophage phenotype. Combinatorial flow analysis of immunomodulatory markers (CD163, CD206, CD209) revealed that the proportion of macrophages co-expressing two immunomodulatory markers was significantly increased on neuraminidase-treated tumor tissue (Fig. 6c, d), suggesting that ECM sialic acids play a role in immunomodulatory changes of macrophage phenotype in the TME. Combinatorial analysis of antigen presentation markers (CD86, HLA-DR) revealed no significant changes (Fig. 6e). Across all conditions, the majority of macrophages expressed Siglec-1 and/or Siglec-9, highlighting a potential mechanistic role for sialic acid–Siglec interactions in macrophage phenotype modulation (Supplementary Fig. 22b).

From whole tissues, analysis of tissue sialylation levels revealed a significant negative correlation between tissue sialylation and CD11b+ macrophage differentiation. Hypersialylation of tumor ECM was associated with a reduction in CD45 + CD11b+ macrophages, consistent with our findings that neuraminidase-mediated desialylation increases the CD45 + CD11b+ macrophage population (Supplementary Fig. 22c, d). These results underscore the role of tumor ECM sialylation in shaping macrophage phenotypes and suggest a functional link between sialic acid levels and immune modulation within the TME.

**Fig. 7:**
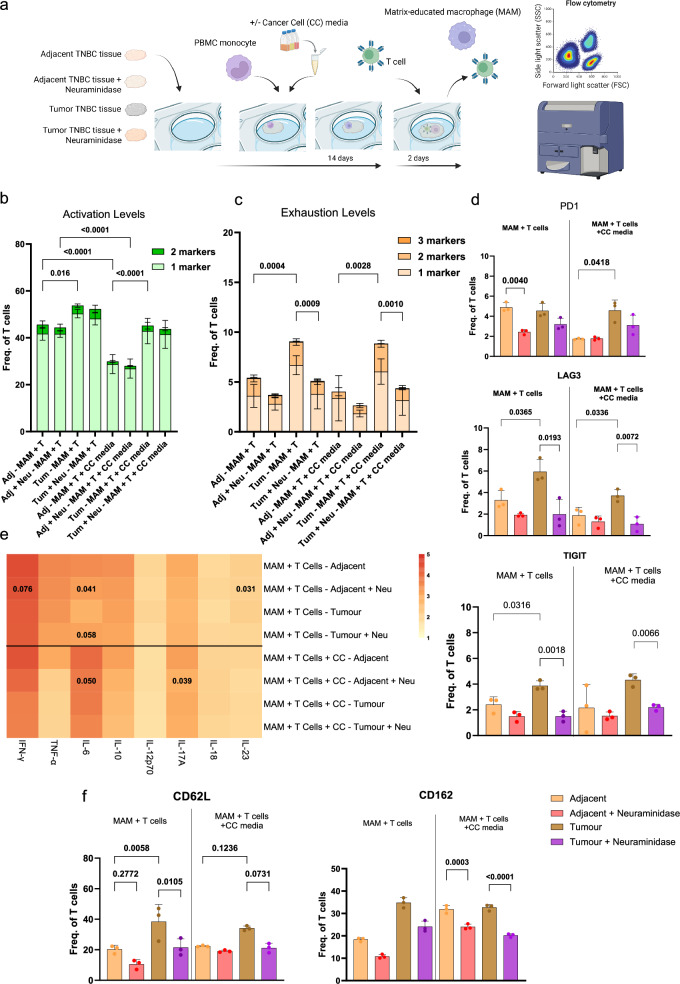
MAMs influence T cell phenotype in a glycan-dependent manner. **a** Schematic of macrophage decellularized tissue culture co-cultured with donor-matched T cells, with or without cancer cell (CC) media. Monocytes from three separate blood donors were cultured for 14 days on tumor (*N* = 3) or adjacent (N = 3) decellularized tissues treated with neuraminidase (Neu) or sodium acetate vehicle control. Flow gating strategy provided in Supplementary Fig. 23. Created in BioRender. Tarantola, L. (2026) https://BioRender.com/ohvhmfy. Stacked bar of **b** activation markers (ICOS & CD137) or **c** exhaustion markers (PD1, LAG3, TIM3) shown as percentage positive cells expressing one marker, two markers or three markers from the T cell population. Error bars represent mean ± SD. 2way ANOVA test. *N* = 3 (*N* = 3 adjacent, *N* = 3 tumor, *N* = 3 blood cones). **d** Barchart of flow cytometry expression patterns of PD1, LAG3 and TIGIT shown as percentage of positive cells from T cell population. Line at mean with standard deviation error bars Mixed-effects analysis with Tukey’s multiple comparisons adjustment test (two-sided). *N* = 3 (*N* = 3 adjacent, *N* = 3 tumor, *N* = 3 blood cones). **e** Heatmap showing log10-transformed concentrations (pg/mL) of 8 cytokines (IFN-γ, TNF-α, IL-6, IL-10, IL-12p70, IL-17A, IL-18 and IL-23) measured in culture supernatants from macrophage (MAM) and T cell co-cultures grown on decellularized adjacent or tumor ECM scaffolds, in the absence or presence of cancer cell–conditioned medium (CC). ECM scaffolds were either untreated or treated with neuraminidase (Neu) prior to cell seeding. *N* = 3 (*N* = 3 adjacent, *N* = 3 tumor, *N* = 3 blood cones). Overlaid numbers indicate paired t-test p-values for comparisons between untreated and Neu-treated ECM within the same tissue and CC condition. **f** Barchart of flow cytometry expression patterns of CD62L and CD162 shown as percentage of positive cells from T cell population. Line at mean with standard deviation error bars. Mixed-effects analysis with Tukey’s multiple comparisons adjustment test (two-sided). *N* = 3 (*N* = 3 adjacent, *N* = 3 tumor, *N* = 3 blood cones).

As we have shown previously that MAMs educated on tumor tissue can affect T cell proliferation and exhaustion markers (Puttock et al.^18^), we sought to determine whether MAMs in TNBC modulate T cell function. We further tested whether soluble factors derived from cancer cells (CC) conditioned media influence MAMs and T cell crosstalk and downstream T cell responses (Fig. 7a, Supplementary Fig. 23). Donor-matched T cells were cultured with MAMs educated on tumor or adjacent tissue with or without neuraminidase treatment and in the presence or absence of CC media, to determine whether these macrophages directly modulated T cell phenotype. Combinatorial analysis of activation markers (CD137 and ICOS) revealed that T cells co-cultured with MAMs on tumor tissue have significantly increased activation compared to adjacent tissue. Moreover, addition of cancer cell soluble factors reduced activation of T cells co-cultured with MAMs on adjacent tissue, whereas on tumor tissues, the influence of cancer cell media on T cell activation was muted (Fig. 7b). This suggests that tumor ECM has a dominant influence on T cell activation, regardless of the presence of cancer cell-derived soluble factors. Furthermore, combinatorial expression of three exhaustion markers (PD1, LAG3 and TIM3) was significantly increased on T cells co-cultured with MAMs on tumor tissue compared to adjacent tissue (Fig. 7c, d, Supplementary Fig. 23b). Interestingly, neuraminidase treatment significantly reduced exhaustion markers on T cells co-cultured with MAMs on tumor tissue, while only inducing a slight reduction on MAMs-educated T cells on adjacent tissue (Fig. 7c, d, Supplementary Fig. 23b). This result indicates a role for the hypersialylated tumor ECM in directing this immunosuppressive macrophage – T cell phenotype. Importantly, cancer cell soluble factors did not have a significant effect on exhaustion marker expression in any condition. Interestingly, T cells cultured alone on decellularized tumor tissue treated with neuraminidase exhibited a smallest reduction in exhaustion marker expression (based on two combinatorial markers), which was not observed on decellularized adjacent tissue. However, overall exhaustion levels in T cells cultured on ECM alone were higher than in co-culture with MAMs. This indicates that the dominant influence on T-cell phenotype arises from interactions with MAMs rather than from direct effects of ECM sialylation on T cells (Supplementary Fig. 24a, b).

To further explore the immunomodulatory function of ECM in macrophage – T cell cross-talk, we performed a cytokine profiling on supernatants from co-cultures of T-cells and macrophages on decellularised models of tumor and adjacent tissue with or without CC media. Cytokine profile analysis revealed that IFN-γ and TNF-α are reduced in the supernatant of MAMs and T cell co-cultures on tumor tissue indicating that tumor ECM influences immune cell cross-talk evidenced by a decreasing immunoinflammatory cytokines release (Fig. 7e and Supplementary Fig. 24c). Interestingly, neuraminidase treatment on both adjacent and tumor tissues reduced the release of anti-tumoral cytokines, such as IL-6, IL-10, IL-17A and IL-23 (Fig.7e and Supplementary Fig 24c), indicating that sialic acid removal decreases pro-tumoral cytokines production, dampening immune suppression, which correlates with the reduction seen in the exhaustion phenotype (Fig. 7c, d).

Finally, we sought to further elucidate the mechanisms by which ECM desialylation enhances T-cell infiltration. Whilst we hadn’t noted a change in Siglec expression (supplementary Figs 17, 18) we wondered whether removal of sialic acids might influence selectin or selectin ligands expression on T cells instead. Cutaneous Lymphocyte Antigen (CLA), CD62L (L-selectin) and CD162 (P-selectin glycoprotein ligand-1, PSGL-1) are highly expressed on T cells alone on our model (Supplementary Fig. 24d, e). Interestingly, MAMs-educated T cells on tumor tissue significantly increase CD62L and CD162 compared to adjacent tissue (Fig. 7f, Supplementary Fig. 23c). This suggests that tumor ECM influence a CD62L and CD162 high phenotype, which is known to dampen the cytotoxic effect of T cells and indicate poor infiltration capacity^25,26^. Neuraminidase treatment on tumor tissues significantly reduces the expression of both CD62L and CD162, indicating a cytotoxic phenotype improvement, decrease of exhaustion and better infiltration in tumor tissue.

Taken together, our results suggest that ECM desialylation enhances T-cell infiltration and motility within tumor nests, reshapes macrophage immunomodulatory programs, and through MAMs–T cell crosstalk reduces T-cell exhaustion, which correlates with decreased release of pro-tumoral cytokines. In parallel, ECM desialylation also modulates selectin and selectin ligand expression on T cells to promote a more cytotoxic, infiltration-competent phenotype, collectively fostering a more immune-permissive tumor microenvironment.

## Discussion

Our study highlights the pivotal role of ECM glycosylation in shaping the tumor immune microenvironment and mediating immune suppression in TNBC in two ways, by controlling T cell trafficking, and educating infiltrating macrophage phenotype that in turn dictates the conversation between macrophages and T cells. We defined here the ECM composition of TNBC, which was associated with significant alterations in glycan presentation on the ECM, particularly elevated sialylation and an increase in immature N-glycan processing resulting in an over representation of high-mannose glycans.

Our data demonstrate that immune-excluded TNBC tumors are characterized by hypersialylated ECM glycoproteins which impede primary T cells, as well as CD8 + CAR T-cells, infiltration into tumor nests. This aligns with previous reports linking glycocalyx sialylation to immune evasion through the engagement of Siglec receptors on immune cells^27–30^ and extends these findings by demonstrating that tumor ECM sialylation itself, rather than just tumor cell surface sialylation, can drive exclusion^31^.

Mechanistically, our study reveals that hypersialylation of the tumor ECM sustains a feed-forward loop of immune suppression in TNBC by restricting effective T cell infiltration and by conditioning immunosuppressive macrophage–T cell crosstalk. Building on our multi-layered ‘omics analyses linking ECM glycosylation to reduced CD8 + T-cell infiltration, live-cell imaging in decellularized, TNBC-recellularized tissues demonstrated that both primary T cells and CAR T-cells infiltrate tumor-derived ECM significantly less efficiently than adjacent tissue and exhibit reduced penetration into tumor nests, recapitulating an immune-excluded phenotype. Moreover, T cells proximal to tumor islets within tumor ECM displayed reduced speed and displacement relative to adjacent ECM, indicating that ECM composition and glycosylation constrain both T cell entry and intratumoral motility. This complements earlier findings by Weaver and colleagues on ECM stiffness and architecture as drivers of malignant progression and immune modulation^7,9,11,32,33^ and aligns with work from Salmon et al., showing that stromal remodeling actively shapes immune cell localization in breast cancer^21^. Overall, our findings provide functional evidence that ECM glycosylation contributes to immune exclusion in TNBC.

Enzymatic modification of ECM glycans via PNGase F or neuraminidase reprogrammed the immune-excluded TME into an immune-inflamed state in our models. This intervention enhanced primary T cell and CAR T-cells infiltration and altered macrophage phenotypes toward a distinct immunomodulatory state. Similar desialylation strategies using neuraminidase mimetics or antibodies have shown preclinical promise in enhancing immune responses by specifically stripping terminal sialic acids from tumor-associated glycans without broad off-target effects^34,35^.

The macrophage data builds on our earlier observations that MAMs can suppress T cell proliferation and drive exhaustion^18^. Here, we demonstrate that tumor ECM sialic acids influence macrophage immunomodulatory phenotype, promote an immunosuppressive phenotype capable of inducing T cell exhaustion and induce the reduction of IFN-γ and TNF-α, indicating dampened immunoinflammatory signaling. Importantly, removal of sialic acids reduces the expression of exhaustion markers and promotes the release of cytokines associated with tumor-supportive inflammation (IL-6, IL-10, IL-17A, IL-23). These cytokines have well-described roles in shaping immunosuppressive tumor microenvironments and myeloid skewing across solid tumors^36–40^. The concordant reduction in exhaustion phenotypes following desialylation supports a model in which ECM sialylation tunes macrophage-driven cytokine milieus that secondarily program T cell dysfunction.

Notably, removal of ECM sialic acids reduced T cell exhaustion mainly in the presence of macrophages, indicating that ECM sialylation acts primarily through macrophage education rather than direct effects on T cells. Similarly, Cao et al. found that genetic deletion of sialic acid biosynthesis in tumor cells improved anti-tumor immunity and immunotherapy response through a macrophage supported interaction with T cells^30^. Together with our findings, this highlights that sialylation within the tumor microenvironment can exert multiple, context-dependent immunological functions. In our system, ECM desialylation primarily alters ligand availability and ECM properties, and while our data are consistent with macrophage education by sialylated ECM, we cannot exclude contributions from sialoglycan–Siglec signaling in this process. Notably, we did not observe changes in Siglec-7 or Siglec-10 expression on T cells following ECM desialylation, although reduced engagement of inhibitory Siglecs by desialylated ECM would be consistent with the increased T-cell infiltration and motility observed. Interestingly, we found that enhanced T-cell infiltration and motility following desialylation may also reflect changes in ECM architecture, creating a more permissive physical environment. This observation suggests that glycan-dependent regulation of ECM organization may influence tissue architecture and barrier properties, potentially through the adhesive properties of glycans bringing fibers together and creating the patterns of ECM seen in disease that are associated with prognosis^41,42^.

We also observed that tumor ECM-educated MAMs induce a CD62L^high and CD162^high (PSGL-1^high) phenotype in T cells, which was reversed by neuraminidase treatment on tumor ECM. The enrichment of this phenotype in tumor ECM conditions is consistent with impaired intratumoral access and effector function, while its reduction following desialylation aligns with improved infiltration and cytotoxic potential. These findings are supported by evidence that PSGL-1 acts as an immune checkpoint-like regulator contributing to T cell exhaustion in tumors^43^, and that elevated CD62L is characteristic of less differentiated/lymph node-homing T cells with limited tissue-infiltrative capacity^25^. Importantly, while neuraminidase treatment altered macrophage surface marker expression (including CD163 and CD209), the limited marker panel used here does not fully resolve macrophage functional states, and such markers should not be interpreted as defining strictly pro- or anti-tumoral phenotypes. Despite marker changes that are classically associated with “TAM-like” states, the net functional consequence of ECM desialylation on MAMs – T cells crosstalk was a reduction in T-cell exhaustion and partial restoration of anti-tumoral T-cell features. Together with our motility data, these results suggest that ECM sialylation indirectly programs T cell trafficking via macrophage education and local cues, further strengthening physical barriers to infiltration.

Our findings suggest that targeting ECM glycosylation could serve as a therapy in combination with existing immunotherapies to overcome immune exclusion. For instance, inhibitors of sialyltransferases or glycan-cleaving enzymes could be explored to disrupt the pro-tumorigenic ECM glycan architecture. Additionally, strategies to enhance immune checkpoint inhibitor efficacy may benefit from concurrent modulation of ECM glycosylation to improve immune cell access to the tumor core sensitizing the tumor to cell-centric immunotherapies.

While our study establishes ECM glycosylation as a key driver of immune exclusion, further in vivo validation is required to confirm the therapeutic potential of targeting glycosylation in TNBC. The heterogeneity of glycosylation patterns across TNBC subtypes and between patients also warrants deeper investigation to identify predictive biomarkers for stratified therapies. Importantly, a related limitation of our study is the integration of immune phenotypes (inflamed, excluded, and desert) with ECM glycosylation features, as intratumoral heterogeneity constrains direct mapping in the decellularised model. Consistent with our previous analysis (Hirani et al.)^14^, 69% of TNBC tissues contained mixed immune phenotypes, which may be reflected in the decellularised models made from tissue. Therefore, the ECM features and immune behaviors observed likely reflect localized microenvironments specific to each model, which should be considered in translational interpretation and addressed in future spatially resolved studies.

In conclusion, our findings reveal that aberrant ECM glycosylation is a critical regulator of immune exclusion in TNBC regulating both macrophage and T cell immunity albeit in different ways either through direct interaction or by changing the crosstalk between cell types. Therapies designed to edit the pattern of glycosylation on ECM could improve both the location and phenotype of immune cells within the TME, sensitizing the tumor to immune cell mediated destruction. The relatively small changes to ECM required to facilitate this process may help to limit off-target or unwanted effects that are more likely to occur in approaches that target ECM biosynthesis. Finally, glycans should be considered attractive targets for therapy design because their biosynthesis and presentation can be disrupted through small molecule drugs that block enzyme activity, glycomimetics that could replace natural sugars whilst altering their immunobiology, or enzymatic degradation of the displayed glycan structure. These findings suggest that to fully understand how the TME regulates immunity and usher in new era of ECM-focused therapies, we must look beyond translation to the post-translational tumor microenvironment.

## Methods

### Ethics approvals for tissue samples

All human female TNBC tissue was obtained from the Barts Cancer Institute Breast Biobank (REC 21/EE/0072). Each patient gave written informed consent. The work was conducted in accordance with the Declaration of Helsinki and International Ethical Guidelines for Biomedical Research Involving Human Subjects (CIOMS). All ethics used for this study were approved by the East of England - Cambridge Central Research Ethics Committee. Patient information is detailed in Supplementary Data 6.

### Isolation of monocytes from peripheral blood samples

Peripheral blood mononuclear cells were isolated from leukocyte cones derived from healthy donors by centrifuging with Ficoll-Paque PLUS (GE Healthcare, 17-1440-03 AG) at 1400 × *g* for 20 min with no deceleration. CD14^+^ monocytes were isolated using CD14 microbeads (Miltenyi, 130-050-201) in LS columns (Miltenyi, 130-042-401) and a QuadroMACS Separator (Miltenyi MACS, 13308).

### Cell culture

The human TNBC BT20, HCC38, and MDA-MB-468 cell lines were cultured in Minimum Essential Medium Eagle (MEM, Lonza), Roswell Park Memorial Institute 1640 medium (RPMI, Gibco), and Dulbecco’s Modified Eagle Medium (DMEM, Gibo), respectively. All the media were supplemented with 10% fetal bovine serum (FBS, Gibco), 1% L-glutamine (Gibco) and 1% penicillin/streptomycin (Gibco) and cells were maintained at 37 °C in a humidified 5% CO_2_ incubator and routinely tested for mycoplasma contamination (Lonza). All cell lines were authenticated by short tandem repeat profiling using the authentication service of the American Type Culture Collection (FTA Sample Collection kits).

### NKG2D-targeting CAR-Ts

Patient matched untransduced T cells and NKG2D (Natural Killer Group 2D) ligand-targeted parallel CAR-T (LEU-011) or MUC-1 ligand-targeting CAR-T were kindly provided by Dr Maher and Dr Davies (Leucid Bio).

### In vitro cytolytic activity, cytokine release, and activation of CAR-T cells

Tumor cells were seeded in triplicate in a 24-well plate (Day 1). After 24 h (Day 2), 1 × 10^5^ CAR-T or control untransduced T cells were added to the target TNBC cells. The supernatant was then harvested 24 h later (Day 3) for enzyme-linked immunosorbent assay (ELISA) measurement of IL-2 (Cat: 88-7025-88, Invitrogen) and IFN-γ (Cat: DY285B-05, Bio-techne) following manufacturer’s protocols. At Day 5, the CAR-T supernatants were harvested, and the target cells were washed with PBS and incubated in 500 μl per well of Thiazolyl Blue Tetrazolium Bromide (MTT, Apollo Scientific BID2165) at 5 mg/ml in PBS diluted in 1:10 in cell media for 1 h in the dark at 37 °C to analyze cytotoxicity. After incubation, the MTT solution was aspirated, and cells were incubated for 5 min in 500 μl/well DMSO to solubilize the formazan crystals. Absorbance was read on a plate reader at 570 nm. CAR-T supernatants were washed and stained then flow cytometry was performed for an activation marker: anti-human CD233 (LAG-3) (BV421, 1:100, Cat: 303415, Biolegend) and Zombie NIR (1:1000, Cat: 423106, Biolegend), to confirm CAR-T activation. Samples were acquired on LSRFortessa (Becton Dickinson) and analysed using FlowJo v10.8.1 (BD FlowJo LLC).

### Tissue transcriptomics

Tissue samples were trimmed at 100 µm thickness, and 50 mg for RNA extraction. RNA was extracted with the RNeasy mini kit (Qiagen, USA), and the concentration was measured by NanoDrop ND1000 UV-Vis spectrophotometer (Thermofisher, USA). The RNA Integrity was evaluated by 4200 TapeStation (Agilent) before RNAseq.

RNA quality control, mRNA library preparation (with RNA-Seq Ribodepletion), RNASeq and data quality control were performed by Oxford Genomics Centre, UK and Dr. Eleni Maniati, Barts Cancer Institute.

### xCell

Log2 base RNA-seq data was delogged and processed to fit the input requirements of each computational method. xCell analysis was performed using the *N* = 64 gene signature and the RNA-seq box ticked.

### Tissue proteomics

Decellularized human breast cancer samples were prepped using our in house whole matrisome proteomics method, optimized to assess whole tissue ECM. Tissues were solubilised in 200 µL 8 M urea buffer, 20 mM HEPES (pH 8) supplemented with protease inhibitors (100 mM Na3VO4, 0.5 M NaF, 1 M β-glycerol phosphate and 0.25 M Na2H2P2O7). Samples were sonicated 5 times for 30 s (on/off), then centrifuged at 17,000 × *g* for 10 min at 4 °C, supernatant transferred into a lo-bind Eppendorf. BCA assay measured protein concentrations by absorbance at 595 nm. Denaturation of protein used 10 µL 500 mM dithiothreitol (DTT) for 1 h in darkness at RT, followed by 20 µl 415 mM iodoacetamide (IAM), for 1 h in darkness at RT. Samples were diluted 1 in 4 with 20 mM 4-(2-hydroxyethyl)-1-piperazineethanesulfonic acid (HEPES), 1500 Units N-glycosidase F (PNGaseF) and incubated at 37 °C for 2 h, followed by 2 µl of 0.8 µg/µl LysC (Endoproteinase) incubated at 37 °C for 2 h. Trypsin beads conditioned with 20 mM HEPES buffer were added to each sample (80 µL) and incubated at 37 °C for 16 h. Samples were centrifuged at 2000 g for 5 min at 4 °C, supernatant transferred to lo-bind Eppendorf. De-salting used C-18 microspin columns (Glygen). Columns were washed with 100% acetonitrile (ACN; LGC), then 99% dH2O (+ 1% ACN, 0.1% Trifluoroacetic acid (TFA)), prior to passing the samples through the columns. Columns were washed with 99% distilled water (dH2O) and transferred into new 1.5 mL lo-bind Eppendorf. Protein samples were eluted in 300 µL 70/30 ACN/H2O + 0.1% formic acid (FA) and lyophilized using a speed-vac, before reconstitution in 0.1% TFA to 0.5 µg/µL and loaded into a Q-Exactive plus mass spectrometer connected to a Ultimate 3000 liquid chromatography (Thermo Scientific).

### Tissue glycomics

Before isolation and purification of glycans from membrane and ECM proteins, we firstly isolated different compartmental proteins from patients tissue: Cytoplasmic, Nuclear, Membrane, Cytoskeleton and ECM Proteins, using CNMCS Compartmental Protein Extraction Kit (Cat no: C6012-25, US Biological) previously described (PMID: 26273955; PMID: 22159717) and also shown in Supplementary Fig. 1, which can enrich ECM proteins from tissues, digest into peptides for Mass Spectrometry Analysis (PMID: 22159717; PMID: 29196464), and avoid glycans mixtures among different protein fractions.

The ECM protein samples were reduced by dithiothreitol (DTT, 10 mg/5 mL) solution in degassed 0.6 M Tris pH 8.5 and incubated at 37 °C for 1 h, and carboxymethylated by iodoacetic acid (IAA, 60 mg/5 mL) in 0.6 M degassed Tris pH 8.5 and incubation of the sample at RT in the dark for 90 min. The homogenate was transferred to a dialysis cassette, and dialyzed for 24 h at 4 °C with continuous stirring. Then the samples were digested by trypsin (1 μg/μL, pH 8.4) and incubated at 37 °C for 14–16 h and the proteolytic digestion was terminated by dispensing 1 drop of acetic acid with a Pasteur pipette. Digested products were purified by reversed-phase chromatography (Classic C18 cartridge, Waters) against propan-1-ol/5% (v/v) acetic acid. N-glycans were released by PNGaseF (Roche) following a 24 h incubation at 37 °C, and were permethylated by sodium hydroxide and methyl iodide in dimethyl sulfoxide. The permethylated glycans were cleaned by Sep-Pak C18 cartridge and freeze dried before mass spectrometry analysis. O-glycans were released by the addition of four hundred microliters of 0.1 M potassium hydroxide containing potassium borohydride (54 mg/ mL) to dried glycopeptide samples and incubated at 45 °C for 14–16 h. The reaction was terminated by adding a few drops of 5% (v/v) acetic acid followed by purification with Dowex 1-X8 desalting column. Excess borates in the samples were subsequently removed by co-evaporating with 10% (v/v) acetic acid in methanol under a stream of nitrogen at room temperature. The purified native O-glycans were then permethylated following the NaOH procedure. Glycan profiling was done on AB Sciex 4800 MALDI-TOF/TOF mass spectrometer. The methylated glycans were dissolved in 10 ul methanol. 1 μL of sample was mixed with 1 μl of 10 mg/ml DABP matrix in 75% ACN. The mixture was spotted on a MALDI plate for MALDI-TOF-MS and MS/MS analysis. N- and O-glycan assignments are based on molecular ion composition information, tandem MS/MS and knowledge of biosynthetic pathways.

### Immunohistochemistry (IHC)

#### panCK and CD8 dual staining

Human TNBC formalin-fixed paraffin-embedded (FFPE) tissue sections were obtained from the BCN tissue bank. Slides were deparaffinized with xylene and rehydrated through a descending ethanol series. Antigen retrieval was performed using a citrate-based unmasking buffer (pH 6, H-3300, Vector). Endogenous peroxidases were blocked with 3% hydrogen peroxide in methanol for 30 min and 2.5% goat serum was used to block tissues for horseradish peroxidase (HRP) staining. Antibodies were diluted in blocking solution and incubated for 1 h at room temperature or 4 °C overnight. HRP stains were detected using Impress-HRP anti-rabbit (MP-7451, Vector). Conditions for each antibody are shown in Supplementary Table 1. 3,3′-Diaminobenzidine reagent (DAB, K3468, DAKO) and Vector VIP (SK-4600, Vector Laboratories) was used to detect dual stains. Hematoxylin counterstain was completed followed by dehydration with an ascending ethanol series and clearing in Xylene. Slides were mounted and scanned using a Pannoramic scanner (3D Histech).

#### ConA and SNA dual staining

Concanavalin A conjugated to AF488 (ConA-AF488) specific for to α-D-mannosyl and α-D-glucosyl residues (C11252, ThermoFisher) and elderberry lectin from Sambucus nigra (SNA, EBL, CY3), which binds preferentially to sialic acid attached to terminal galactose in α-2,6 and to a lesser degree, α-2,3 linkage (CL-1303-1, Vector Laboratories), were used for the lectin stains of tissue. Samples were blocked in 5% BSA PBS for 1 h then washed and treated with 400 μg/mL ConA-AF488 or 20 μg/mL SNA-FITC for 1 h at room temperature before a further wash and visualization. Tissues were stained with DAPI (1:10,000, Cat: 40043, Biotium). Slides were mounted with anti-fade mounting media (H-1000, Vector Laboratories) and scanned with Nanozoomer S60 (Hamamatsu, Japan).

### Immunohistochemistry spatial analysis

QuPath 0.5.1^44^ was used for image analysis of the TNBC tissues. panCK staining was used to threshold for tumor cells and then used as a mask and annotation for tumor area. Expansion of the tumor annotation was used to create a mask and annotation for stroma area surrounding tumor nests. CD8 staining was used to threshold for T cell detection using StarDist segmentation and then we analysed T cells within tumor and stroma annotations^45^.

### TNBC tissue decellularization

TNBC tissues were decellularized according to Puttock et al.^18^. In brief, tissue samples were frozen at −80 °C and sliced into small sections using a Vibratome. Sections were then washed under agitation with a hypertonic buffer solution (Tris 10 mM, EDTA 5 mM, PMSF 0.1 mM, H_2_O) for 4 h at room temperature (RT), 100% acetone overnight at 4 °C, washed twice with PBS + 1% pen/strep, then 4% or 2.5% sodium deoxycholate was added to the tumor or surrounding/adjacent tissues respectively, for 4 h at RT, the tissue washed as before, next benzonase solution (Tris 50 mM, MgCl_2_ 1 mM, BSA 0.1% (w/v), benzonase 40units/ml, H_2_O) for 20 h at 37 °C. The samples were then washed as before and clean PBS + 1% pen/strep added, and the samples were then left in agitation at 4 °C for 48 h with a change in PBS + pen/strep. Decellularised tissue was then stored at −80 °C.

### Nucleic acid

Nucleic acid was extracted from the decellularised samples using the DNeasy blood and tissue kit (Qiagen) according to the manufacturer’s instructions and quantified using a NanoDrop 2000 (ThermoFisher).

### SEM

Tissue samples were fixed for 3 h with 2.5% glutaraldehyde in water at room temperature. The hydrogels were gradually dehydrated with increasing concentrations of ethanol (20, 50, 70, 80, 90, 96, and 100%, v/v), twice per solution for 10 min. Dehydrated samples were then subjected to critical point drying (K850, Quorum Technologies, UK). SEM micrographs of the tissue were acquired on Inspect F50 (FEI Comp, the Netherlands) after sputter-coating with gold.

### Tissue stiffness

Tissue stiffness was measured by Instron 3342 with a 10 N load cell. The samples were applied an initial tare load of 0.1 N, and subjected to a 20% uniaxial unconfined compressive strain at a strain rate of 20%/min. This was followed by a stress relaxation period in which the load was recorded for a further 300 s. Stress–strain and stress–time curves were generated for each specimen and the mechanical properties of the samples determined according to the following equations where, σ1 and σ2 are the stress at 18% and 20% strain during the loading phase.

### Histochemistry

Tissue samples were cut with a scalpel and a sample from each was preserved in formalin overnight at 4 °C. The samples were then paraffin embedded and sliced using a microtome. Haematoxylin and eosin (H&E), and Masson’s trichrome staining was then performed using standard protocols.

### Collagen alignment analysis

Masson’s trichrome stain was performed and collagen ECM patterns were analyzed using The Workflow Of Matrix BioLogy Informatics (TWOMBLI) as described before^46^.

### Immunohistochemistry

Paraffin embedded tissue slides were heated at 60 °C to remove paraffin and then rehydrated. Slides were then placed into an unmasking buffer (Vector) and incubated in a pressure cooker for antigen retrieval, except for fibronectin staining. Hyaluronic acid and chondroitin sulphate stains were then enzyme treated for 2 h at 37 °C. All slides were then treated with an endogenous peroxidase solution (H_2_O_2_ 2% (v/v) in methanol). Tissue samples on the slides were circled with a PAP pen (DAKO). Blocking buffer (2.5% BSA (w/v), 2.5% goat serum (v/v) in 1X PBS) was then added and the primary antibody solution was then added to the slides with blocking buffer; fibronectin (FN1) (1:500, Abcam: ab23750), versican (VCAN) (1:200, ATLAS: HPA004726), collagen 1A1 (COL1A1) (1:300, ATLAS: HPA011795), chondroitin sulphate (CS) (1:600, Abcam: ab11570), cathepsin B (CTSB (1:400, Abcam: ab58802), cartilage oligomeric matrix protein (COMP) (1:80, Abcam: ab11056), and incubated overnight at 4 °C. The secondary antibody and blocking buffer (1:200) was then added. ABC kit vectastain (Vector labs) was added and washed and then DAB reagent (DAKO) was added and allowed to develop. Slides were then counterstained with 50% Gills haematoxylin and dehydrated. Hyaluronic acid (HA) staining followed the same steps as above until the primary, which was added and then incubated for 1 h at RT instead, (1:100, Merck: 385911). These stains were then washed and did not require a secondary antibody, so the slides had ABC vectastain added and followed the same steps as above from then onwards. Slides were then imaged using a Pannoramic 250 slide scanner (3DHistech) and quantified using Definiens™ software.

### Staining score

Stain data including the intensity marker stain area (IMSA) was collected from Definiens, and was analysed using the following equation in order to produce the staining score for that image.

$$({{\mathrm{highIMSA}}}\times 3)+({{\mathrm{mediumIMSA}}}\times 2)+({{\mathrm{lowIMSA}}})({{\mathrm{totaltissuearea}}}\times 3)100$$

### Decellularized tissue cancer cell culture

Breast adjacent/surround and tumor tissue was sliced using a scalpel into pieces roughly 5 mm in diameter and then decellularized as described previously. This decellularized tissue was then placed into a 96-well plate. 100,000 cells from each of the cultured cell lines was added to each of the gels in 10 μl of media, these were then incubated for 2 h 5% CO_2_ incubator at 37 °C before 200 μl media added to each tissue. The next day, tissues and their attached cells were then transferred to a 96-well plate with 200 μl of media and incubated for a further 6 days in a CO_2_ incubator, with a media change every 2–3 days.

### Live/Dead

LIVE/DEAD Viability/Cytotoxicity Kit (Cat: L3224, Thermo Fisher Scientific) was performed according to manufacturer’s instructions and imaged using a Nikon Eclipse TE Spinning Disk Confocal microscope.

Decellularized slice CAR-T cell co-culture, immunostaining and spinning disk confocal real-time timelapse imaging and analysis

The immunostaining method was adapted from Laforêts et al.^47^. Slices were transferred to a coated plastic petri dish and a stainless steel ring washer was placed around the tumor slice to hold drops of antibody mix, washing medium and T cells/CAR-Ts. Slices were incubated with an antibody mix diluted in phenol red-free RPMI 1640 medium (Cat: 11835030, Thermo Fisher Scientific) for 30 min at 37 °C and washed twice 2 min with phenol red-free RPMI 1640. T cells/CAR-T cells were stained at 37 °C with an antibody mix in phenol red-free RPMI 1640 medium for 15 min, and then washed in phenol red-free RPMI 1640 and then plated onto the recellularized slices at effector:target ratios of 1:1 for 1 h at 37 °C, before the slices were transferred to a glass bottom dish. 3 mL phenol red-free RPMI1640 was added to the imaging dish after securing the slice with a tissue slice anchor.

The real-time imaging method was performed according to Laforêts et al.^47^. In brief, tumor slices were imaged with a Nikon Eclipse TE equipped with a spinning disk and a temperature-controlled chamber set on 37 °C and 5%CO_2_ with phenol red-free RPMI 1640 bubbled with carbogen (95% O_2_, 5% CO_2_) perfused in the imaging dish.

Analysis was performed according to Laforêts et al.^47^. In brief, still-frame images were analyzed in 3D and time-lapse recordings were projected to a single z-plane (maximum intensity projection) with the Nikon NIS-Elements software and imported in Imaris 9.1 (Oxford Instruments, Bitplane) for analysis of CAR-T movement and location within the tissue slice.

### Decellularized tissue slice glycan cleavage enzyme treatment

N-glycans of the decellularised tissue were cleaved using the amidase PNGase F (Cat: P0704S, New England Biolabs), according to the manufacturer’s protocol with slight modifications. Tissue samples were plated into a flat bottom 96-well plate and 15 μL of PNGase F was used with 10 μL of Glycobuffer in a final volume of 115 μL made up with ddH_2_O per well. Samples were incubated at 37 °C for 24 h. Terminal sialic acid residues on N- and O- linked glycans were removed using a Neuraminidase Clostridium perfringens (Cat: 11585876001, Roche). Neuraminidase (5 U/ml in a final volume of 100 μL acetate buffer at pH 5) was added onto tissue samples in a 96-well plate. Samples were incubated at 37 °C for 24 h. After treatment with either PNGase F or Neuraminidase samples were washed with PBS + 1% P/S.

### Decellularized tissue slice lectin staining

ConA- AF488 specific for to α-D-mannosyl and α-D-glucosyl residues (C11252, ThermoFisher); SNA-FITC, specific for sialic acids with the α2,6 linkage (FL-1301-2, Vector Laboratories); Maackia Amurensis Lectin I (MALI), Fluorescein (FL-1311-2, 2B Scientific), specific for sialic acids with α2,3 linkage; Lycopersicon Esculentum (Tomato) Lectin (LEL), DyLight 488 (FL-1151, 2B Scientific) which recognizes poly-LacNAc structures; Galanthus Nivalis Lectin (GNL) Fluorescein (FL-1241-2, 2B Scientific) which recognizes high mannose structures; and PNA From Arachis hypogaea (peanut), AlexaFluor 488 (L21409, Thermo Fisher Scientific), specific for Galβ1-3GalNAc linkage found in O-glycans were used for the lectin stains of decellularized tissue. Samples were blocked in 5% BSA PBS for 1 h then washed and treated with 200 μL 100 μg/mL ConA-AF488 or 10 μg/mL SNA-FITC, MALI, LEL, GNL, PNA for 1 h at RT before a further wash and imaging using the Nikon TE Eclipse microscope.

### Decellularized tissue slice CAR-T cell Siglec phenotype flow cytometry

After co-culture on decellularized tissue for 1 h at 37 °C, CAR-T cells were collected by gently pipetting up and down to detach cells. Cells were then washed and stained with a flow cytometry antibody cocktail and analyzed by flow cytometry. Staining panel used: CD4 (BV605, 1:250, Cat:, Biolegend), CD8 (AF647, 1:100, Cat: 344725, Biolegend), Siglec-5 (PE, 1:100, Cat: 352003, Biolegend), Siglec-7 (AF700, 1:100, Cat: 339209, Biolegend), Siglec-9 (BV421, 1:100, Cat: 743363, BD Biosciences), Siglec-10 (PE Cy7, 1:100, Cat: 347607, Biolegend), Siglec-15 (AF488, 1:100, Cat: FAB9227G, R&D Systems), Zombie NIR (1:1000, Cat: 423106, Biolegend),

### Decellularized tissue macrophage culture

Sliced decellularized tissues were equilibrated with RPMI 10% FBS 1% L-Glutamine 1% P/S at 4 °C overnight. Decellularized tissues were removed from RPMI 10% FBS 1% L-Glutamine 1% P/S and excess liquid removed. Decellularized tissue slices were placed into the wells of 96-well plate. To the decellularized tissues, 20 μL of isolated monocytes were added to the center of the tissue at seeding density of 2 × 10^5^/20 μL. The monocytes were incubated with the decellularized tissue at 37 °C for 2 h to allow the cells to attach to the tissue. After 2 h, 200 μL RPMI 10% FBS 1% L-Glutamine 1% P/S was added carefully to the cultures, not to disturb the tissue adhered cells. Cultures were maintained for 14 days and media changed every 48 h.

### Decellularized tissue macrophage flow cytometry

Macrophages were harvested, washed and stained with a flow cytometry antibody cocktail and analyzed by flow cytometry. Staining panel used: CD45 (FITC, 1:100, Cat: 368508, Biolegend), CD36 (BV421, 1:100, Cat: 336229, Biolegend), CD163 (BV605, 1:100, Cat: 333616, Biolegend), CD86 (BV650, 1:100, Cat: 105035, Biolegend), HLADR (BV711, 1:100, Cat: 307643, Biolegend), Siglec-9 (BV786, 1:100, Cat: 743366, BD Bioscience), CD209 (APC, 1:100, Cat: 330107, Biolegend), SIRpa (AF700, 1:100, Cat: 323816, Biolegend), Siglec-1 (PE, 1:100, Cat: 346003, Biolegend), CD11b (AF594, 1:100, Cat: 301340, Biolegend), CD206 (PE-Cy7, 1:100, Cat: 321123, Biolegend), Fixable Viability Dye eFluor™ 780 (1:1000, Cat: 65-0865-14, Invitrogen). Samples were acquired on a LSR Fortessa II (BD Bioscience) and analysed using FlowJo v10.8.1 (BD FlowJo LLC).

### T cell isolation

Pan-T cell negative selection was performed by magnetic cell sorting (Pan T Cell Isolation Kit, human, 130-096-535, Miltenyi) from frozen PBMCs of autologous samples according to the manufacturer’s instructions. Isolated T cells were activated for 24 h using T Cell TransAct™, human (130-128-758, Miltenyi) with 1:500 IL-2 (1 × 10^5^ IU/mL, 200-02-100UG, ThermoFisher) according to manufacturer’s instructions.

### Decellularized tissue T cell and macrophage co-culture

Activated T cells were collected and stained with CellTracker Green (C2925, ThermoFisher) in RPMI, 1:1000 for 30 min at 37 °C. Decellularized tissues with macrophages were removed from media, and 1 × 10^5^/20 μL activated T cells were added to the center of the tissue. The T cells were incubated with the decellularized tissue at 37 °C for 2 h to allow the cells to attach to the tissue. After 2 h, 200 μL RPMI supplemented with 10% HS (human serum, H4522-100ML, Sigma-Aldrich) + 1:500 IL-2 was added carefully to the cultures, not to disturb the tissue adhered cells. Co-culture was maintained for 48 h. In some conditions, cancer cell media derived from HCC38 were used in 1:1 ratio with RPMI to culture macrophages and macrophages – T cell co-culture.

### Decellularized tissue T cell and macrophage co-culture flow cytometry

At the end of the 14 days, tissues were dissociated with 1 mg/ml Liberase TL (05401020001, Sigma) and T cells and macrophages collected for flow cytometry staining. Staining panel used: PD1 (PerCP, 1:100, Cat: 329937, Biolegend), CD206 (AF700, 1:100, Cat: 321132, Biolegend), CLA (AF647, 1:100, Cat: 321310, Biolegend), TIM3 (BUV805, 1:100, Cat: 368-3109-42, ThermoFisher), CD8 (BUV563, 1:100, Cat: 612915, BD Bioscience), CD11b (SparkUV 387, 1:100, Cat: 301366, Biolegend), ICOS (BV785, 1:100, Cat: 313533, Biolegend), CD62L (BV711, 1:100, Cat: 304860, Biolegend), Siglec-9 (BV650, 1:100, Cat: 743366, BD Bioscience), CD163 (BV605, 1:100, Cat: 333616, Biolegend), TIGIT (BV510, 1:100, Cat: 372737, Biolegend), LAG3 (BV421, 1:100, Cat: 369314, Biolegend), CD209 (PE-Cy7, 1:100, Cat: 330114, Biolegend), CD162 (Pe, 1:100, Cat: 328806, Biolegend), Fixable Viability Dye eFluor™ 780 (1:1000, Cat: 65-0865-14, Invitrogen).

Samples were acquired on a BD Symphony A3 1 (BD Bioscience) and analysed using FlowJo v10.8.1 (BD FlowJo LLC).

### Cytokine quantification by flow cytometry

At the end of 48 h macrophage–T cell co-culture, supernatants were collected and diluted 1:1 in RPMI. The concentration of eight cytokines (IFN-γ, TNF, IL-6, IL-10, IL-12p70, IL-17A, IL-18 and IL-23) was measured by flow cytometry using a multiplex bead-based assay (Cat: 740809, LEGENDplex™; Biolegend), according to the manufacturer’s instructions. Briefly, standards and cell culture supernatant samples were incubated with capture beads in polypropylene 96-well V-bottom plates for 2 h. After washing the plate, biotinylated detection antibodies were added to each well and incubated for 1 h. Streptavidin-phycoerythrin (SA-PE) was subsequently added and incubated for 30 min. The plate was washed, and samples were resuspended in wash buffer and transferred to 5 mL FACS tubes for analysis. Sample acquisition was performed on a LSR Fortessa II (BD Bioscience) flow cytometer using FACSDiva 6.0 software (BD Biosciences, San Diego, CA, USA), and the data were analyzed with LEGENDplex™ Data Analysis Software. Cytokines were identified based on the bead size, their intrinsic fluorescence, and the fluorescent signal emitted from the anti-cytokine antibody/SA-PE complex. Cytokine concentration in samples was determined from the geometric mean fluorescence intensity of PE interpolated on the standard curves calculated from eight standard dilutions measured in duplicate.

### Statistical analysis

All statistical analyses were performed using either GraphPad Prism software version 8.3.0 for Windows, GraphPad Software, San Diego, California, USA, www.graphpad.com or the statistical programming language RStudio (2022.02.3  +  492 “Prairie Trillium” Release) and R (version 4.1.1) using the following software plugins: Hmisc for correlation analysis, gplots for correlation scatter plots, ggplot2 for bar charts, pheatmap for heatmaps, dendextend for dendrograms and ggpubr for editing Figs to publication standard. Multivariate correlations were calculated using Spearman’s or Pearson’s correlation as appropriate, applied on linear or log transformed data, where *p*  <  0.05 is considered significant unless otherwise specified and indicated with asterisk: *p  <  0.05, **p  <  0.01, ***p  <  0.005. Statistical tests used were indicated in the Fig. legends.

### Reporting summary

Further information on research design is available in the Nature Portfolio Reporting Summary linked to this article.

## Supplementary information

Supplementary Information

Transparent Peer Review file

Reporting Summary

Description of Additional Supplementary Files

Supplementary Data 1

Supplementary Data 2

Supplementary Data 3

Supplementary Data 4

Supplementary Data 5

Supplementary Data 6

## Source data

Source Data

## Data Availability

The RNAseq data have been deposited in NCBI’s Gene Expression Omnibus under the accession number GSE318780^48,49^. The mass spectrometry proteomics data have been deposited to the ProteomeXchange Consortium via the PRIDE partner repository under the dataset identifier PXD075558^50^. Glycomics data was uploaded to Zenodo 10.5281/zenodo.18595849. Source data are provided with this paper.
