# Supplementary material for: Glycosylated extracellular matrix drives immune suppression by modulating macrophage-T cell crosstalk in triple-negative breast cancer
Source: Nat Commun. 2026 Jun 16;17:5008. doi: 10.1038/s41467-026-73467-5 (PMC13273098; doi:10.1038/s41467-026-73467-5)
Supplement: Supplementary file 1 — Supplementary Information [file 41467_2026_73467_MOESM1_ESM.pdf]

Glycosylated extracellular matrix drives immune suppression by modulating macrophage-T cell crosstalk in triple-negative breast cancer

Tarantola, L[1,a], Tyler, EJ[1,a], Liu, Y[1,a], Maniati, E[1], Thornton, KA[1], Martín-Otal, C[1], Hanna, D[1], Kumar, R[1], Gauthier, V[1], Hirani, P[1], Burger Ramos, M[1], Roth, NJ[1], Bragg, J[1], Puttock, EH[1], McDermott, J[1], Rajeeve, V[1], Cutillas, P[1], Maiques, O[1], Soulier, A[2], Correa de Sampaio, P[2], Jones, JL[1], Davies, DM[3], Maher, J[3], Haslam, SM[4], Läubli, H[5] & Pearce, OMT*[1].

[1]Queen Mary University of London, Barts Cancer Institute, John Vane Science Centre, London EC1M 6BQ, UK.

[2]Neobe Therapeutics, Salisbury House, Station Road, Cambridge CB1 2LA, UK

[3]Leucid Bio, Guy's Hospital, Great Maze Pond, London SE1 9RT, UK.

[4]Department of Life Sciences, Imperial College London, London, UK

[5]Department of Biomedicine and Division of Medical Oncology, University Hospital Basel, Hebelstrasse 20, 4031, Basel, Switzerland

* Corresponding author: Pearce, O.M.T. (o.pearce@qmul.ac.uk)

[a] These authors contributed equally: Tarantola, L.; Tyler, E.J.; Liu, Y.

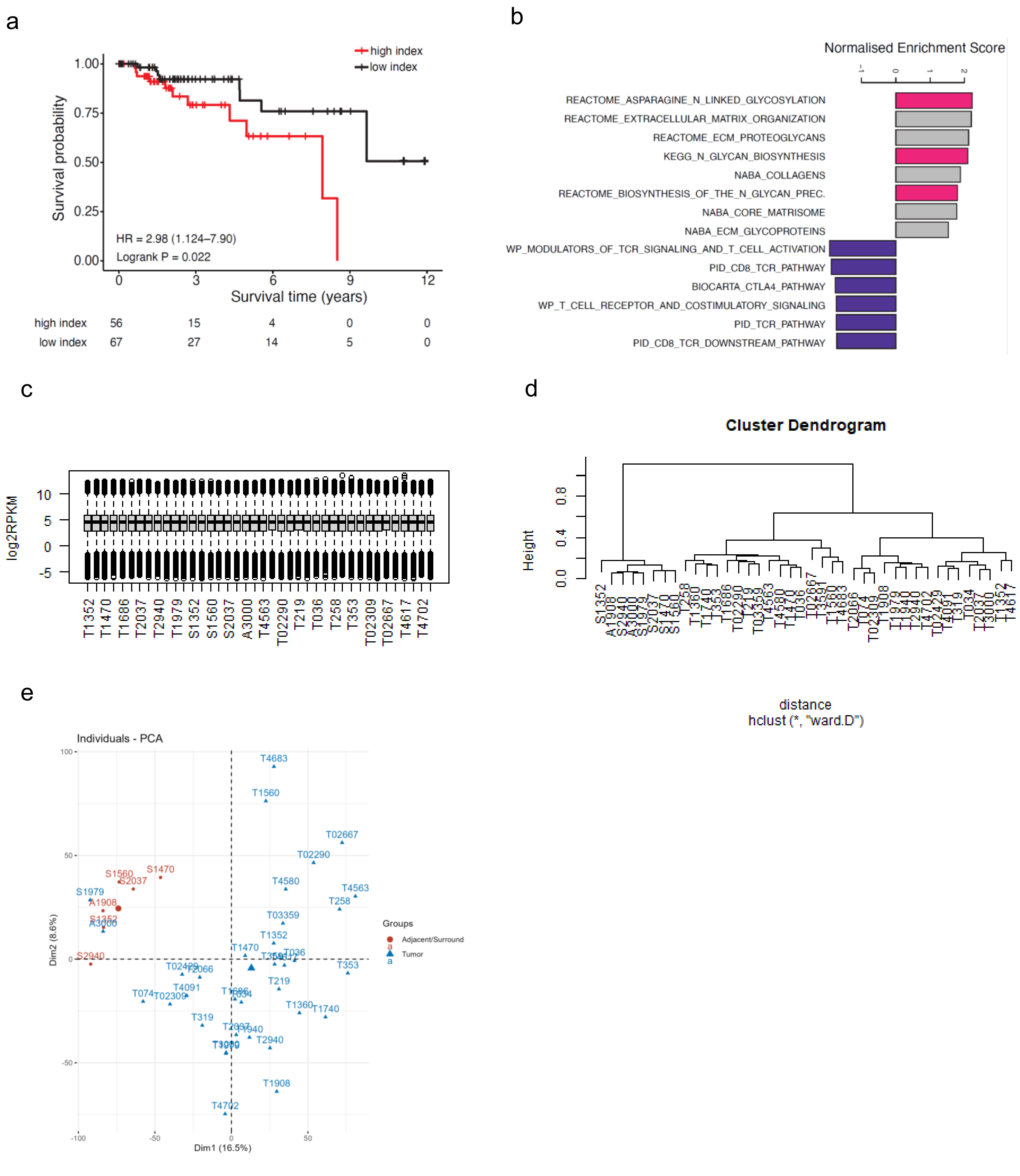

Supplementary Fig. 1: Characterisation and transcriptomic analysis of TNBC patient tissues. a, Kaplan-Meier survival curve for matrix index (MI) high (56 patients) vs MI low (67 patients) from TCGA invasive breast cancer dataset. **b,** Differentially expressed genes between MI high and MI low patient samples are enriched for genes upregulated in ECM remodelling (grey bars) and post-translational glycosylation (pink bars) and genes downregulated in immunity (blue bars). **c,** Exploratory boxplot for sample quality. Boxplots illustrate median (centre of the box) with the upper (Q3: 75th percentile) and lower (Q1: 25th percentile) quartiles (ends of the box); the whiskers correspond to Q3 + 1.5 x Interquartile Range (IQR) to Q1 – 1.5 x IQR; dots beyond the whiskers show potential outliers. N=. **d,** Unsupervised cluster analysis using all genes. **e,** Principal Component Analysis (PCA) using all genes.

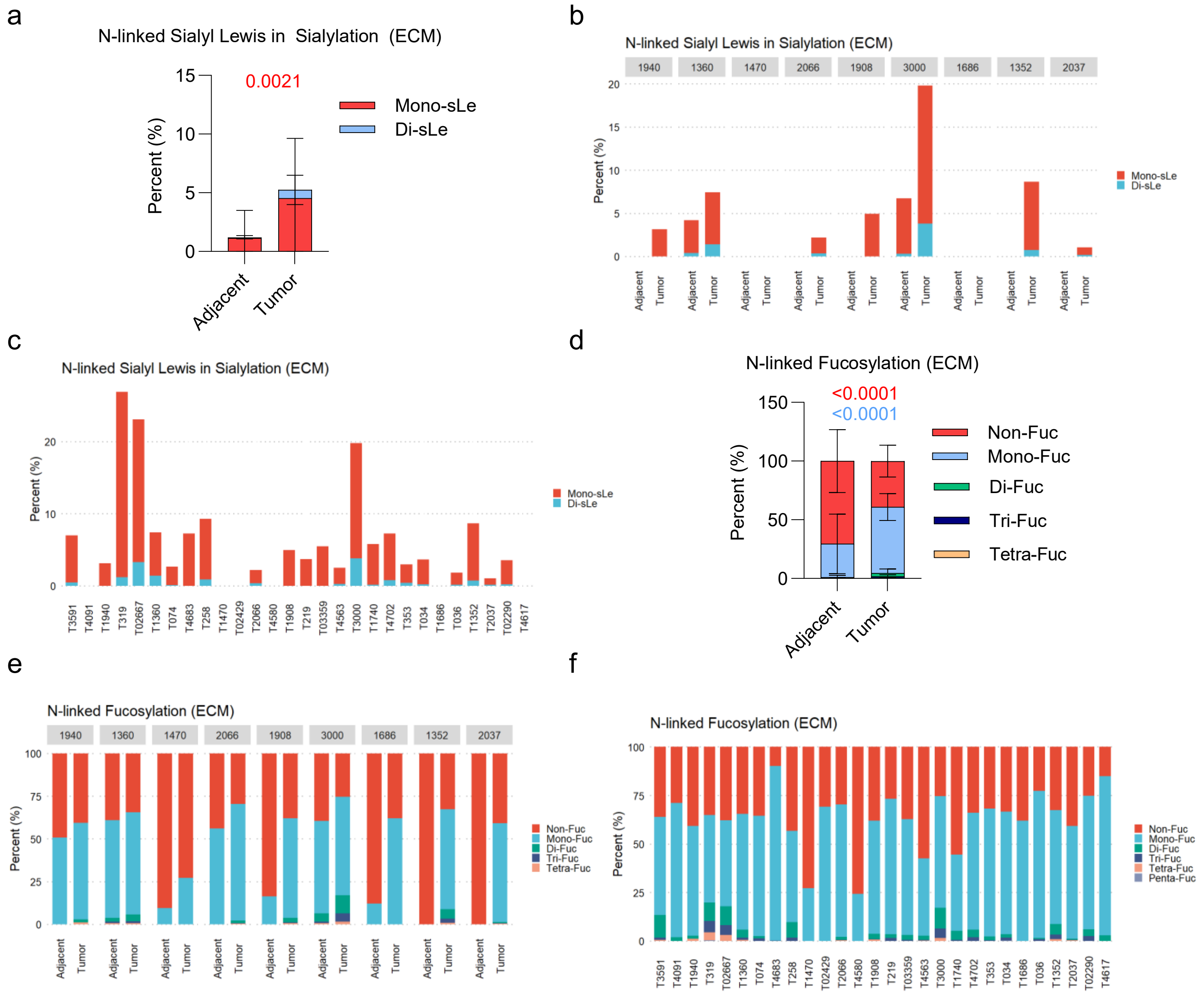

Supplementary Fig. 2: ECM N-linked glycomics analysis. ECM N-linked glycans assessed for **a-c,** Sialyl Lewis groups (Mono-sialyl lewis (sLe), red; Di-sLe, light blue) , **d-f,** Fucosylation (Non-fucosylation (Fuc), red; Mono-Fuc, light blue; Di-Fuc, green; Tri-Fuc, dark blue; Tetra-Fuc, peach). **a, d,** average adjacent/surround and tumor samples, Mean with SD. Two-way Repeated Measures ANOVA with Šídák's multiple comparisons test, N=9 each. N=9 each. **b, e,** paired adjacent/surround and tumor samples, N=9 each. **c, f,** all tumor samples ranked by MI. N=28.

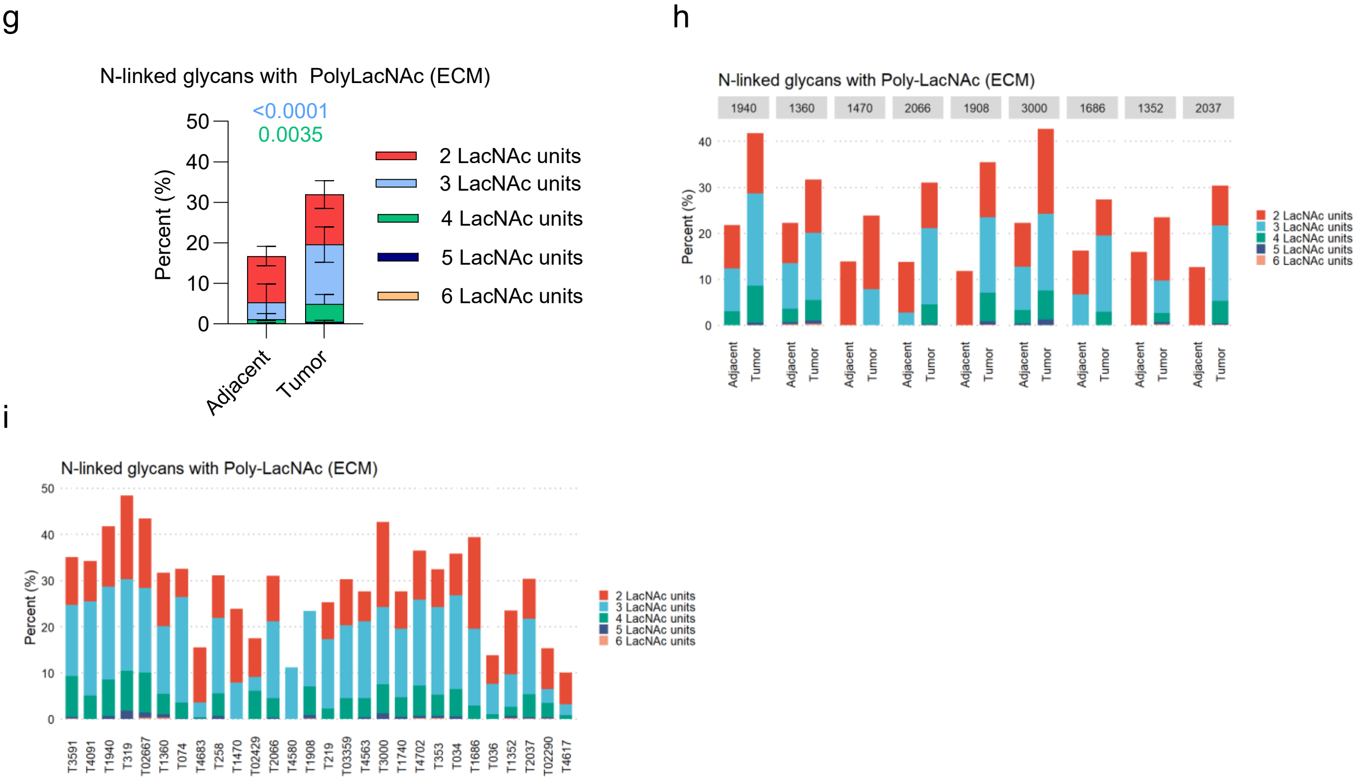

Supplementary Fig. 2: ECM N-linked glycomics analysis. ECM N-linked glycans assessed for **g-i,** Poly-LacNAc (2 LacNAc units, red; 3 LacNAc units, light blue; 4 LacNAc units, green; 5 LacNAc units, dark blue; 6 LacNAc units, peach) structures. **g,** average adjacent/surround and tumor samples, Mean with SD. Two-way Repeated Measures ANOVA with Šídák's multiple comparisons test, N=9 each. N=9 each. **h,** paired adjacent/surround and tumor samples, N=9 each. **i,** all tumor samples ranked by MI. N=28.

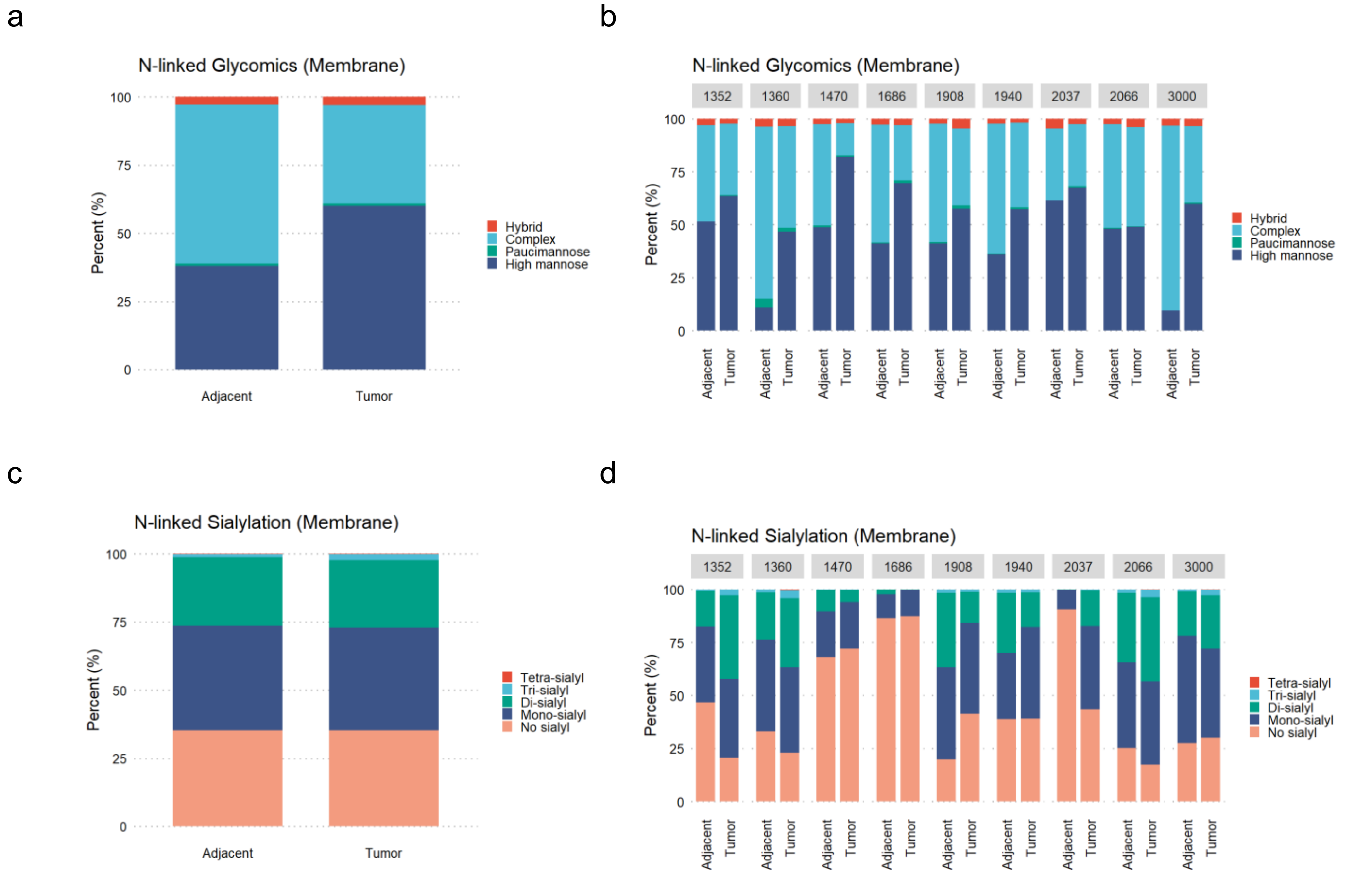

Supplementary Fig. 3: Membrane N-linked glycomics analysis. a-b, Membrane N-linked glycans assigned to four main groups (Complex, dark blue; Hybrid, green; High-mannose, light blue; Paucimannose, red). Stacked barchart displaying the proportion of each family in the N-glycome for **a,** mean adjacent/surround and tumor samples and **b,** paired adjacent/surround samples and tumor samples. N=9 adjacent/surround tissues, N=9 tumor tissues. **c-d,** Membrane N-linked complex glycans broken down into five groups (Non-Sialyl, peach; Mono-Sialyl, dark blue; Di-Sialyl, green; Tri-Sialyl, light blue; Tetra-sialyl, red). Stacked barchart displaying the proportion of each family in the N-glycome **c,** mean adjacent/surround and tumor samples and **d,** paired adjacent/surround samples and tumor samples. N=9 adjacent/surround tissues, N=9 tumor tissues.

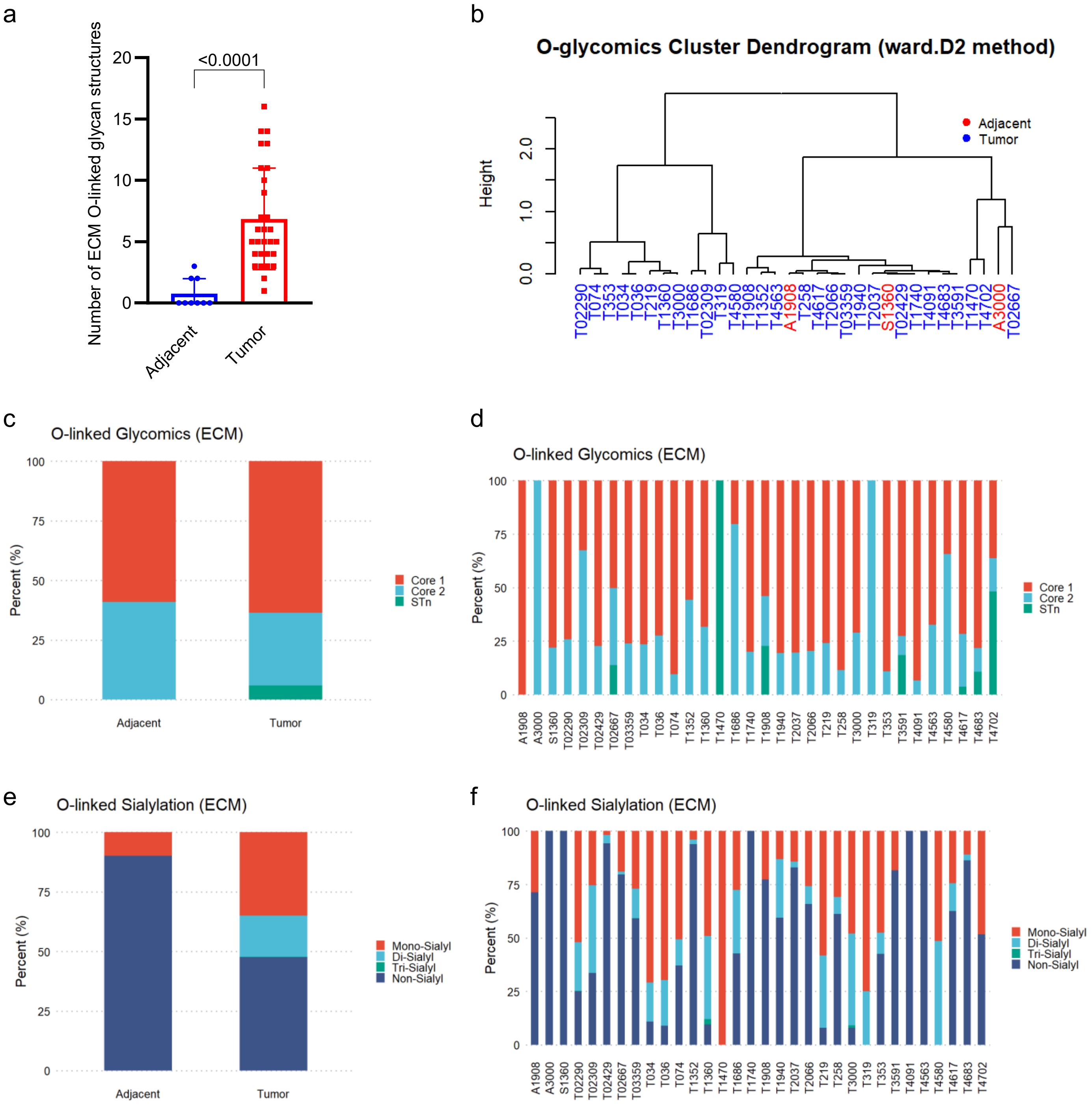

Supplementary Fig. 4: ECM O-linked glycomics analysis. a, Number of ECM O-linked glycan structures detected. Mean with SD. Mann Whitney test. N=9 for adjacent/surround ('adjacent'), N=29 for tumor tissues. **b,** Unsupervised cluster dendrogram using ECM O-linked glycan structures from adjacent/surround and tumor tissues. **c-d,** ECM O-linked glycans assigned to three main groups (Core 1, red; Core 2, light blue; STn, green). Stacked barchart displaying the proportion of each family in the O-glycome for **c,** mean adjacent/surround and tumor samples and **d,** all adjacent/surround samples and tumor samples. N=3 adjacent/surround tissues, N=29 tumor tissues. **e-f,** ECM O-linked complex glycans broken down into five groups (Non-Sialyl, dark blue; Mono-Sialyl, red; Di-Sialyl, light blue; Tri-Sialyl, green). Stacked barchart displaying the proportion of each family in the O-glycome **e,** mean adjacent/surround and tumor samples and **f,** all adjacent/surround samples and tumor samples. N=3 adjacent/surround tissues, N=29 tumor tissues.

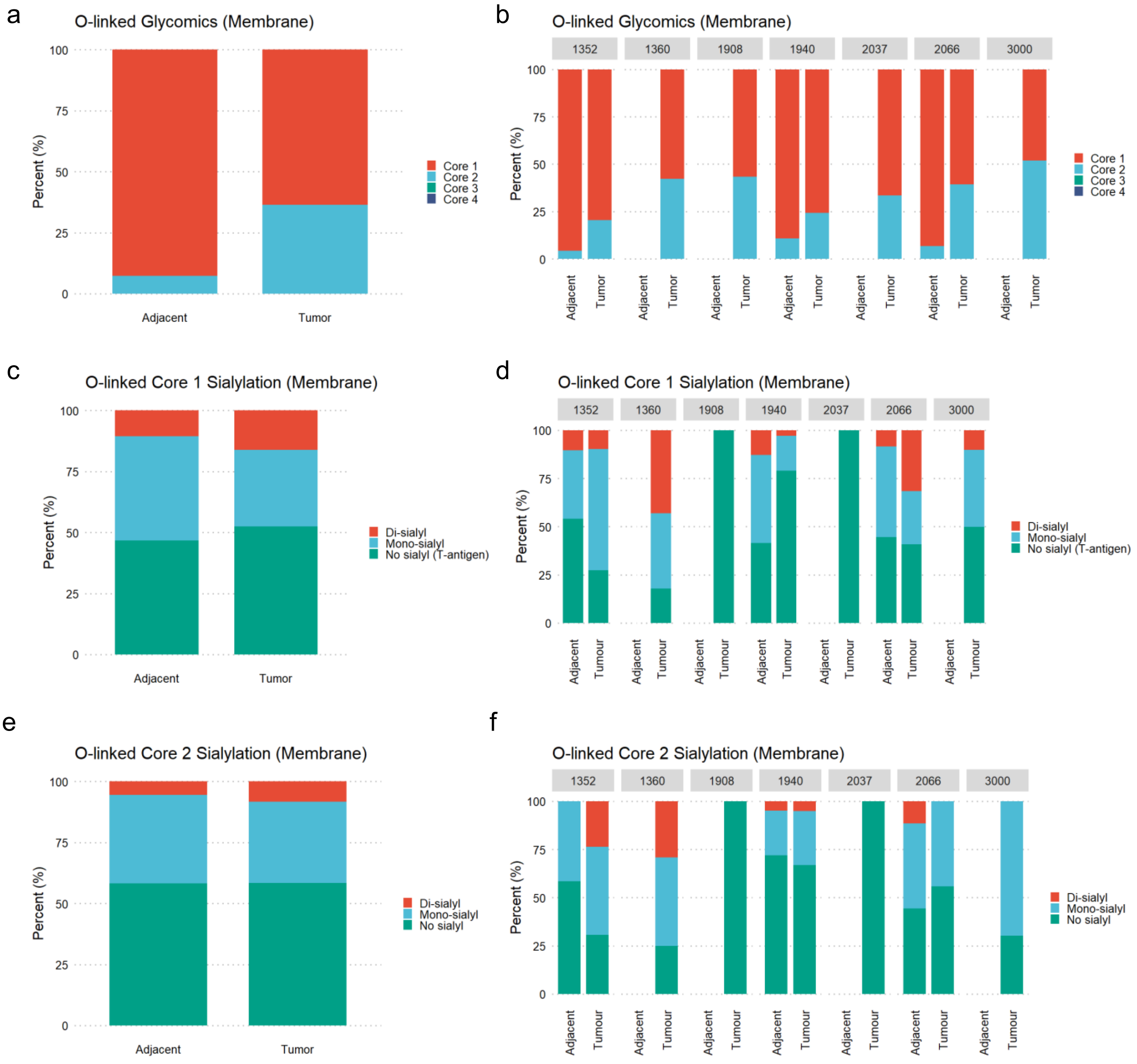

Supplementary Fig. 5: Membrane O-linked glycomics analysis. a-b, Membrane O-linked glycans assigned to four main groups (Core 1, red; Core 2, light blue; Core 3, green; Core 4, dark blue). Stacked barchart displaying the proportion of each family in the O-glycome for **a,** mean adjacent/surround and tumor samples and **b,** paired adjacent/surround samples and tumor samples. N=3 adjacent/surround tissues, N=7 tumor tissues. **c-d,** Membrane O-linked Core 1 glycans broken down into three groups (Di-sialyl, red; Mono-sialyl, blue; No Sialyl (T-antigen), green). Stacked barchart displaying the proportion of each family in the O-glycome **c,** mean adjacent/surround and tumor samples and **d,** paired adjacent/surround samples and tumor samples. N=3 adjacent/surround tissues, N=7 tumor tissues. **e-f,** Membrane O-linked Core 2 glycans broken down into three groups (Di-sialyl, red; Mono-sialyl, blue; No sialyl, green). Stacked barchart displaying the proportion of each family in the O-glycome **e,** mean adjacent/surround and tumor samples and **f,** paired adjacent/surround samples and tumor samples. N=3 adjacent/surround tissues, N=7 tumor tissues.

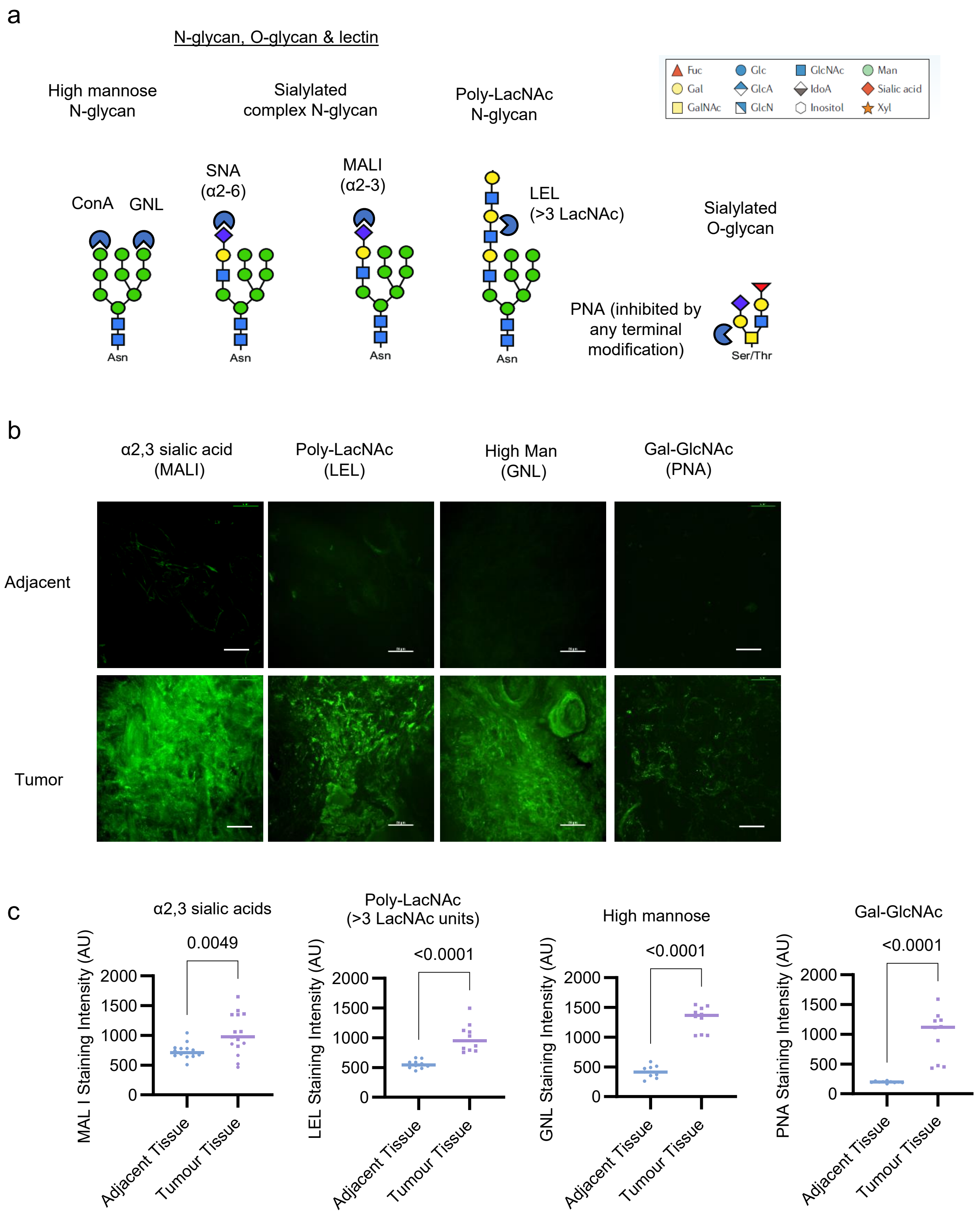

Supplementary Fig. 6: Lectin staining for N-linked and O-linked glycan structures in tumor and adjacent decellularized TNBC patient tissues. a, Schematic representing N-linked and O-linked glycan structures and their respective lectin binding partners. **b,** Representative images of matched adjacent and tumor TNBC patient tissue stained with Maackia Amurensis Lectin I (MALI), Fluorescein (FL-1311-2, 10µg/mL), Lycopersicon Esculentum (tomato) Lectin (LEL) Dylight 488 (DL-1174-1, 10µg/mL), Galanthus Nivalis Lectin (GNL) Fluorescein (FL-1241-2, 10µg/mL), and PNA From Arachis hypogaea (peanut), AlexaFluor 488 (L21409, 10µg/mL). Scale bar = 50µm. **c,** Quantitation of lectin staining intensity using IMARIS image analysis software. Median. Unpaired t test. N=2 matched patient tissues each, 5 FOV per tissue.

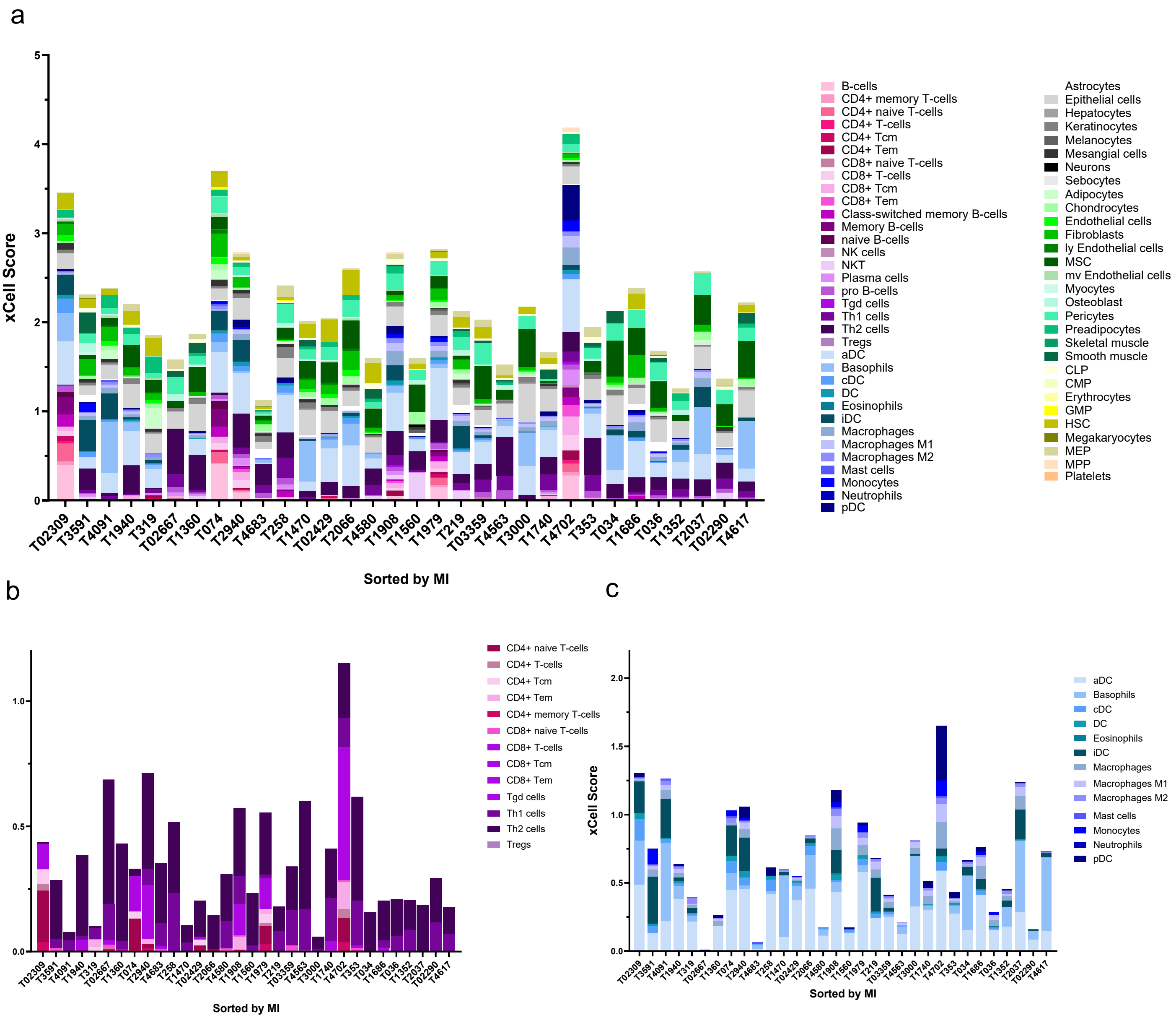

Supplementary Fig. 7: xCell deconvolution of TNBC tumor tissues. a, Stacked barchart of 64 xCell signatures for each patient tumor tissue. N=32. **b,** Stacked barchart of xCell lymphoid compartment signatures. N=32. **c,** Stacked barchart of xCell myeloid compartment signatures. N=32.

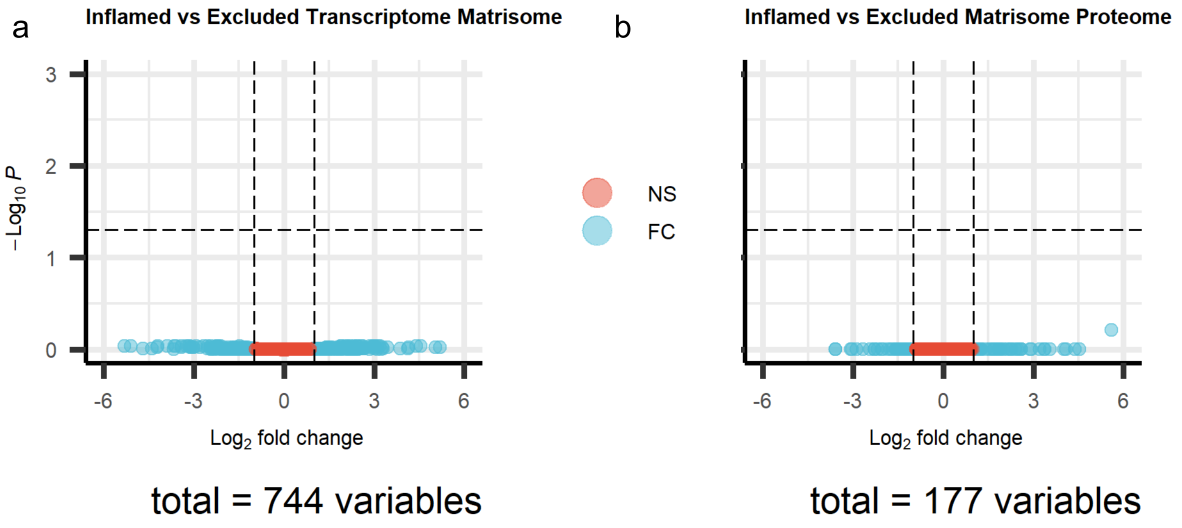

Supplementary Fig. 8: No changes in matrisome transcriptome or proteome between inflamed and excluded samples. a, Volcano plot of differentially regulated matrisome genes between inflamed and excluded tumor tissues. N=4 for excluded, N=3 for inflamed. **b,** Volcano plot of differentially regulated matrisome proteins between inflamed and excluded tumor tissues. N=4 for excluded, N=5 for inflamed.

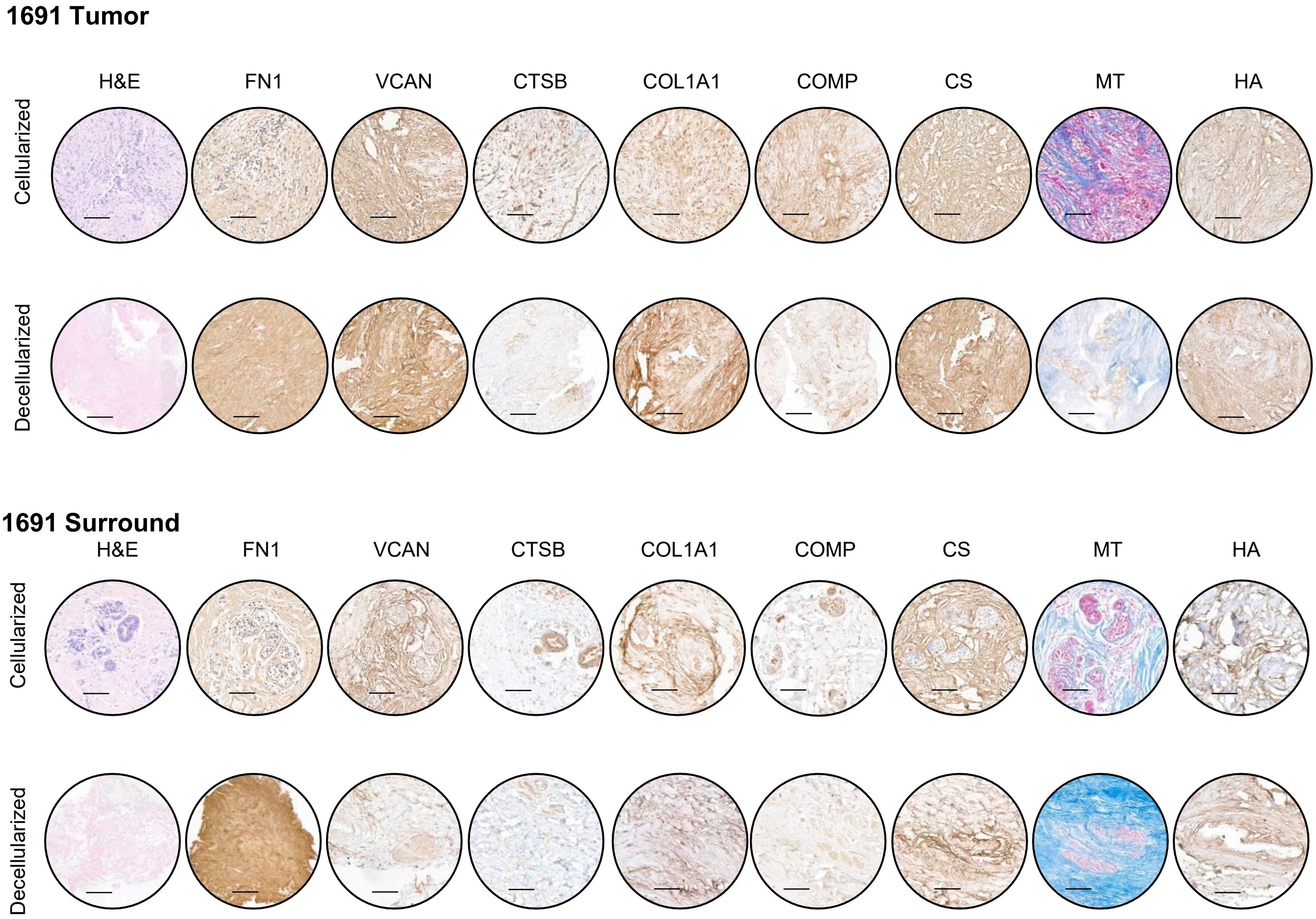

Supplementary Fig. 9:. Representative sections of IHC staining for matrix molecules in tumor and surround cellularized and decellularized TNBC patient tissues. Stains included anti-FN1 (ab23750, 1:500), anti-VCAN (HPA004726, 1:200), anti-CTSB (ab58802, 1:400), anti-COL1A1 (HPA011795, 1:300), anti-COMP (ab11056, 1:80), anti-CS (ab11570, 1:600), Masson's Trichrome (MT), and anti-HA (385911, 1:100). Scale bar = 100μm.

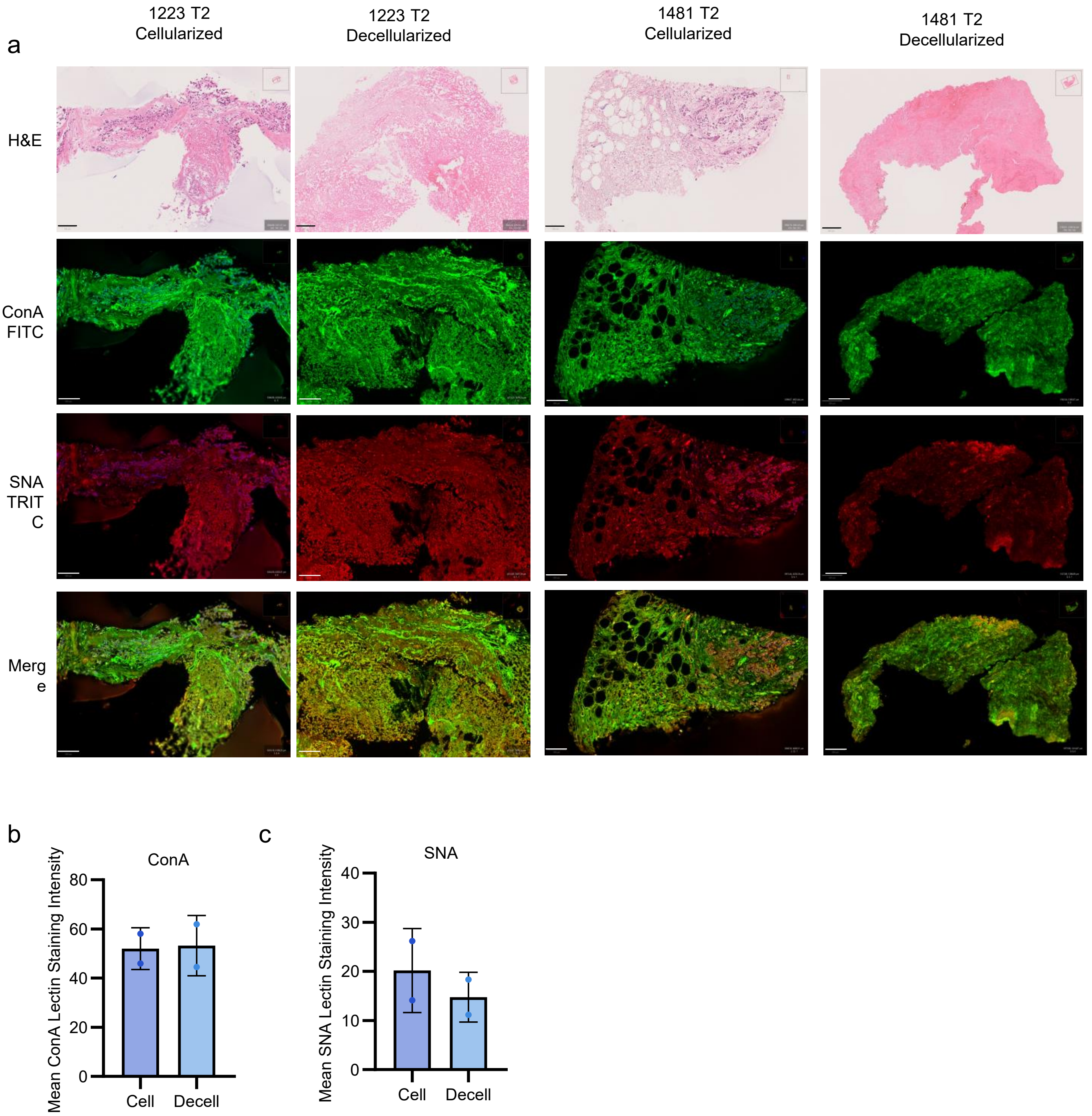

Supplementary Fig. 10: Lectin staining confirms that decellularization does not affect N-linked and sialylated glycan structures in tumor and adjacent TNBC patient tissues. a, Representative images of cellularized or decellularized adjacent or tumor tissue. H&E staining in the top row, staining of a consecutive section with Concavalin A (ConA) lectin conjugated to AF488 for all N-linked glycans in the second row (green) and Sambucus nigra (SNA) lectin conjugated to Cy3 for sialic acids in the third row (red). Scale bar = 100µm. **b-c,** Analysis of **b,** ConA-AF488 and **c,** SNA-Cy3 lectin staining intensity using QuPath. N=2 matched tissue donors for each group, paired T test.

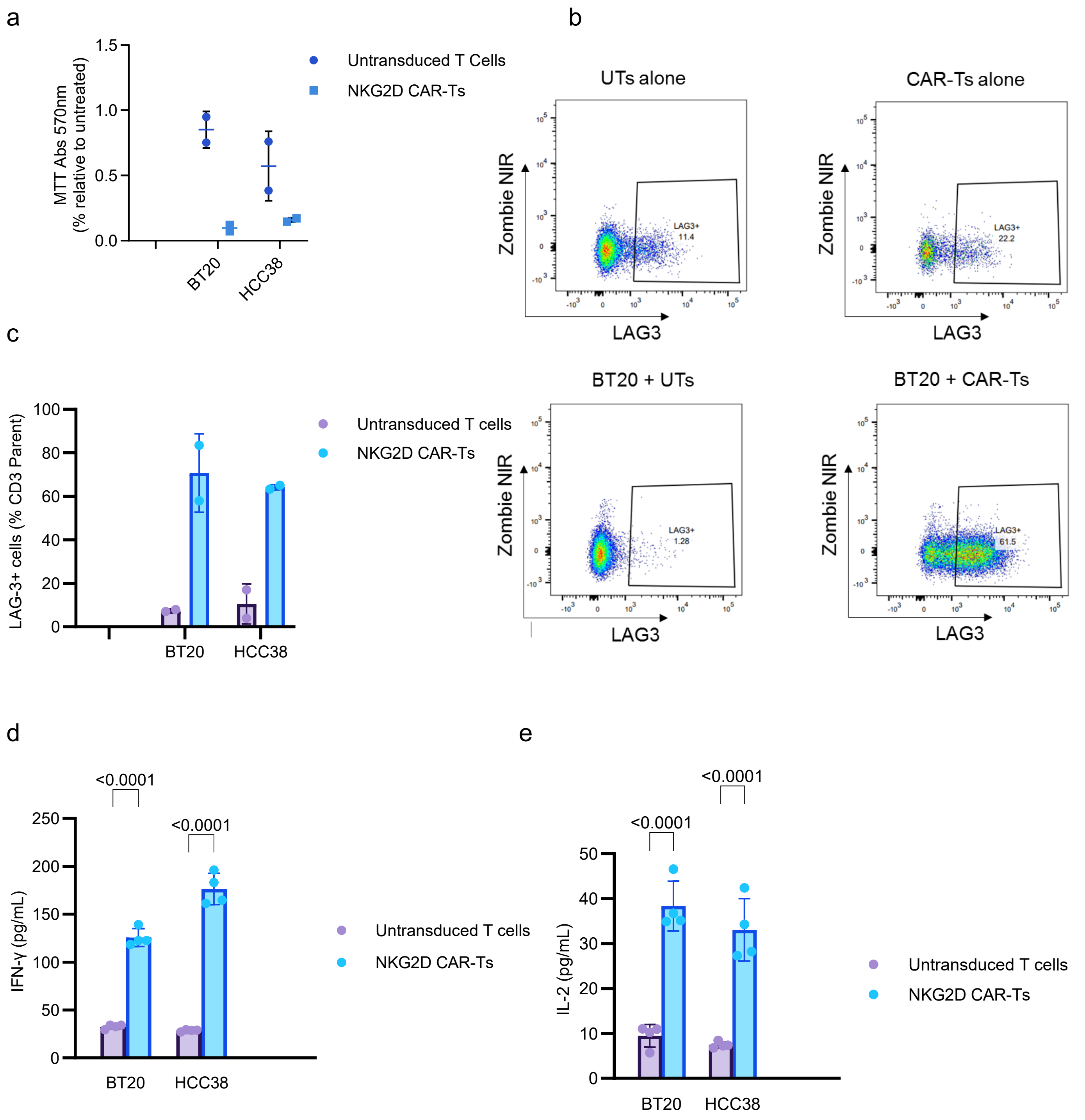

Supplementary Fig. 11: CAR-T targeting of TNBC cell lines in 2D. a, MTT assay with three TNBC cell lines, MDA-MB-468, BT20 and HCC38 co-cultured with untransduced T cells or CAR-Ts targeting NKG2D. Mean with SD. Two-way ANOVA with Šídák's multiple comparisons test. N=2. **b,** Representative flow cytometry plots. **c,** Bar plots of flow cytometry expression patterns of LAG3 expression, shown as percentage of CD3+ parent cells. Mean with SD. Two-way ANOVA with Šídák's multiple comparisons test. N=2. **d-e,** ELISA was performed to detect secretion of **d,** IFN-γ and **e,** IL-2 by NKG2D-targeting CAR-Ts. Mean with SD. Two-way ANOVA with Šídák's multiple comparisons test. N=4.

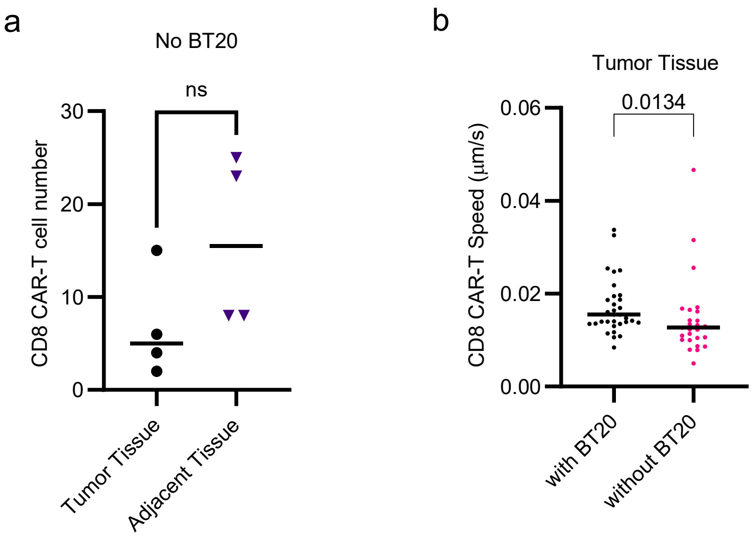

Supplementary Fig. 12: Decellularized tissue timelapse video analysis. a-b, Timelapse confocal microscopy of NKG2D-targeting CAR-Ts in decellularized tissues which were not seeded with BT20 TNBC cells. **a,** Mean CD8 NKG2D-targeting CAR-T cell number per field of view (FOV). Line at median. Unpaired t test. N=4. **b,** Mean speed of CD8+ CAR-Ts. Line at median. Mann Whitney test. N=4.

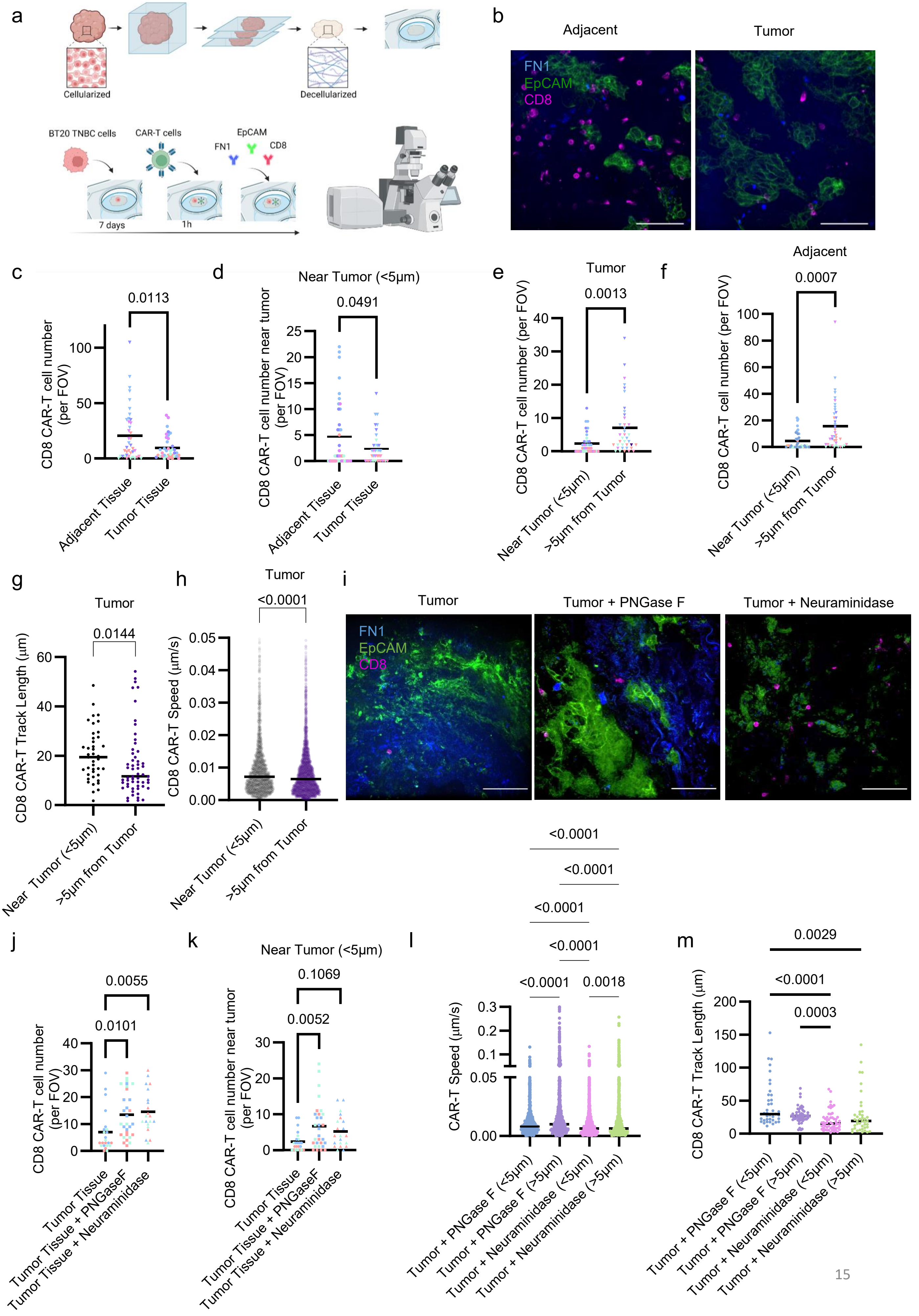
a
Cellularized
Decellularized
BT20 TNBC cells
CAR-T cells
EpCAM
FN1
CD8
7 days
1h
b
Adjacent
Tumor
FN1
EpCAM
CD8
c
CD8 CAR-T cell number (per FOV)
0.0113
Adjacent Tissue
Tumor Tissue
d
Near Tumor (<5μm)
CD8 CAR-T cell number near tumor (per FOV)
0.0491
Adjacent Tissue
Tumor Tissue
e
Tumor
CD8 CAR-T cell number (per FOV)
0.0013
Near Tumor (<5μm)
>5μm from Tumor
f
Adjacent
CD8 CAR-T cell number (per FOV)
0.0007
Near Tumor (<5μm)
>5μm from Tumor
g
Tumor
CD8 CAR-T Track Length (μm)
0.0144
Near Tumor (<5μm)
>5μm from Tumor
h
Tumor
CD8 CAR-T Speed (μm/s)
<0.0001
Near Tumor (<5μm)
>5μm from Tumor
i
Tumor
Tumor + PNGase F
Tumor + Neuraminidase
FN1
EpCAM
CD8
j
CD8 CAR-T cell number (per FOV)
0.0055
0.0101
Tumor Tissue
Tumor Tissue + PNGaseF
Tumor Tissue + Neuraminidase
k
Near Tumor (<5μm)
CD8 CAR-T cell number near tumor (per FOV)
0.1069
0.0052
Tumor Tissue
Tumor Tissue + PNGaseF
Tumor Tissue + Neuraminidase
l
CAR-T Speed (μm/s)
<0.0001
<0.0001
<0.0001
<0.0001
<0.0001
0.0018
Tumor + PNGase F (<5μm)
Tumor + PNGase F (>5μm)
Tumor + Neuraminidase (<5μm)
Tumor + Neuraminidase (>5μm)
m
CD8 CAR-T Track Length (μm)
0.0029
<0.0001
0.0003
Tumor + PNGase F (<5μm)
Tumor + PNGase F (>5μm)
Tumor + Neuraminidase (<5μm)
Tumor + Neuraminidase (>5μm)

Supplementary Fig. 13: Decellularized TNBC tissue maintains an immune excluded TME in vitro and modifications to ECM-glycans can convert the TME to an immune inflamed phenotype. a, Schematic of fluorescence-based live confocal imaging assay using decellularized tissue seeded with BT20 TNBC cells and NKG2D-targeting CAR-T co-culture. Tissues stained with anti-human CD326 (EpCAM) AlexaFluor (AF) 488 (53-8326-42, 1:10) anti-human CD8-AF647 (344725, 1:10)) and anti-human fibronectin conjugated to Dylight 405 (F3648, 1:10). Created in BioRender. Tyler, E. (2026) https://BioRender.com/f72b95i. **b,** Representative images of adjacent and tumor decellularized tissue recellularized with BT20 TNBC cells (anti-EpCAM-AF488, green) and CAR-T cells (anti-CD8-AF647, purple) with the extracellular matrix stained with anti-FN1-AF405, blue. Scale bar = 100µm. **c-d,** Count of **c,** CD8 CAR-T cell number in each field of view (FOV) and **d,** CD8 CAR-T cell number within 5µm of a tumor cell ('near tumor') and **e-f,** CD8 CAR-T cell number near (>5µm) and far (>5µm) from a tumor cell in **e,** tumor and **f,** adjacent decellularized tissue recellularized with BT20 cells. N=6 tissue donors for each group, 5-10 FOV per tissue. Unpaired T-test. **g,** CD8+ CAR-T cell speed (µm/s) near (<5µm) and far (>5µm) from tumor cells in tumor tissue. **h,** CD8+ CAR-T cell track length (µm) near (<5µm) and far (>5µm) from tumor cells in tumor tissue. **e-h,** Line at median. Mann-Whitney test. N=4, with 2 technical replicates each. **i,** Representative images of tumor control tissue and tumor tissue treated with either PNGase F or neuraminidase enzyme treatment, and recellularized with BT20 TNBC cells (anti-EpCAM-AF488, green), and CAR-T cells (anti-CD8-AF647, purple). Extracellular matrix stained with anti-FN1-AF405 (blue). Scale bar = 100µm. **j-m,** Count of **j,** CD8 CAR-T cell number in each FOV and **k,** CD8 CAR-T cell number within 5µm of a tumor cell ('near tumor'). N=3 tissue donors, 6-10 FOV per tissue. One-way ANOVA with Dunnett's multiple comparison's test. **l,** CD8+ CAR-T cell speed (µm/s) near tumor cells. **m,** CD8+ CAR-T cell track length (µm) near tumor cells. **j-m,** Line at median. Kruskal-Wallis test with Dunn's multiple comparisons test. N=3, with 2 technical replicates each.

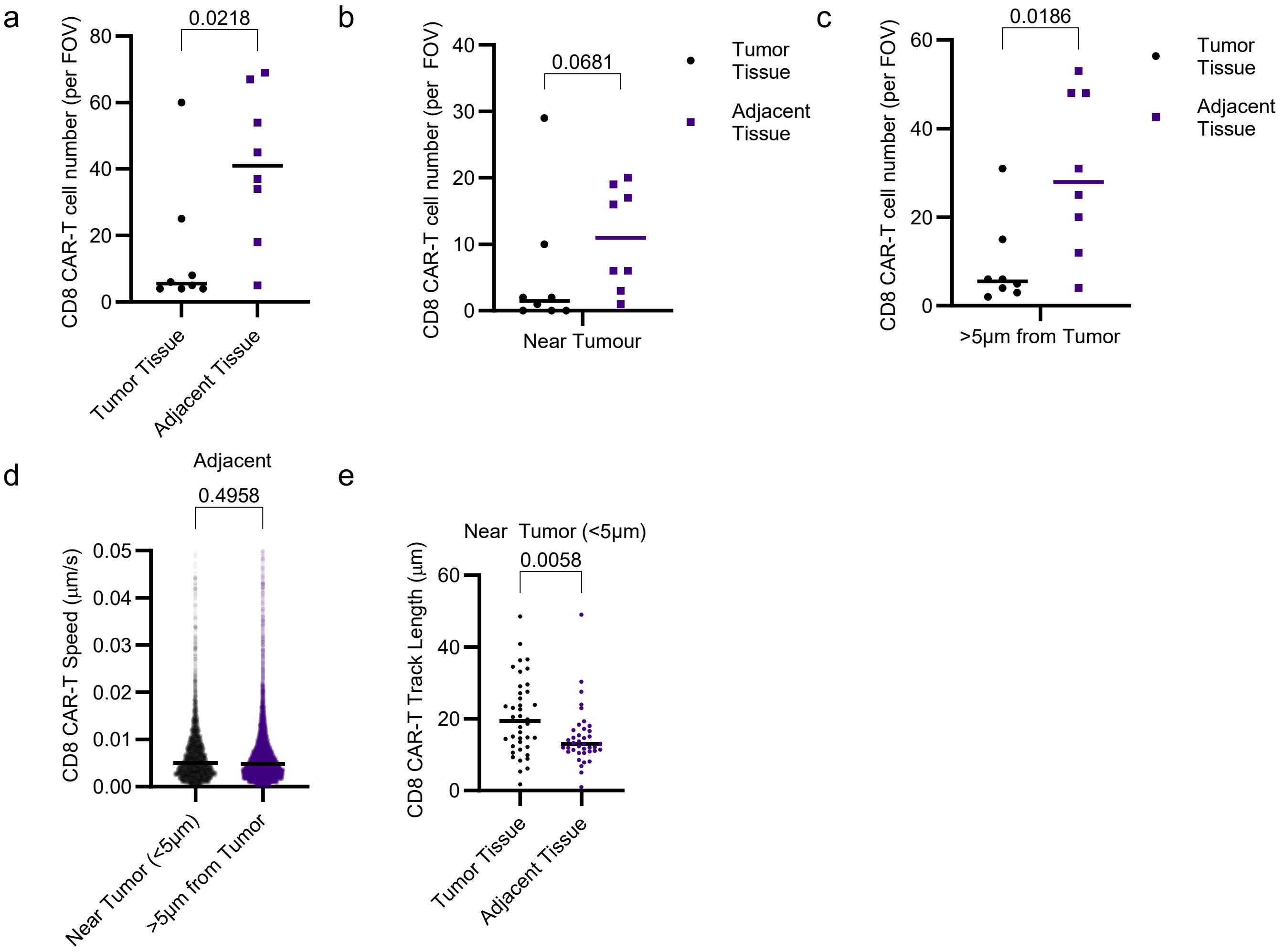

Supplementary Fig. 14:. Decellularized tissue timelapse video analysis. Fluorescence-based live confocal timelapse imaging assay using decellularized tissue seeded with BT20 TNBC cells and NKG2D-targeting CAR-T co-culture. Tissues stained with anti-human CD326 (EpCAM) AlexaFluor (AF) 488 (53-8326-42, 1:10) anti-human CD8-AF647 (344725, 1:10)) and anti-human fibronectin conjugated to Dylight 405 (F3648, 1:10). Video analysis performed using IMARIS analysis software. **a,** CD8+ CAR-T cell number per field of view (FOV) in tumor and adjacent tissue. **b,** CD8+ CAR-T cell number per field of view (FOV) near EpCAM+ tumor cells (<5µm) in tumor and adjacent tissue. **c,**CD8+ CAR-T cell number per field of view (FOV) >5µm from tumor cells in tumor and adjacent tissue. **d,** CD8+ CAR-T cell speed (µm/s) >5µm from tumor cells and **e,** CD8+ CAR-T cell track length (µm) >5µm from tumor cells in adjacent tissue. **a-e,** Line at median. Mann-Whitney test. N=4, with 2 technical replicates each.

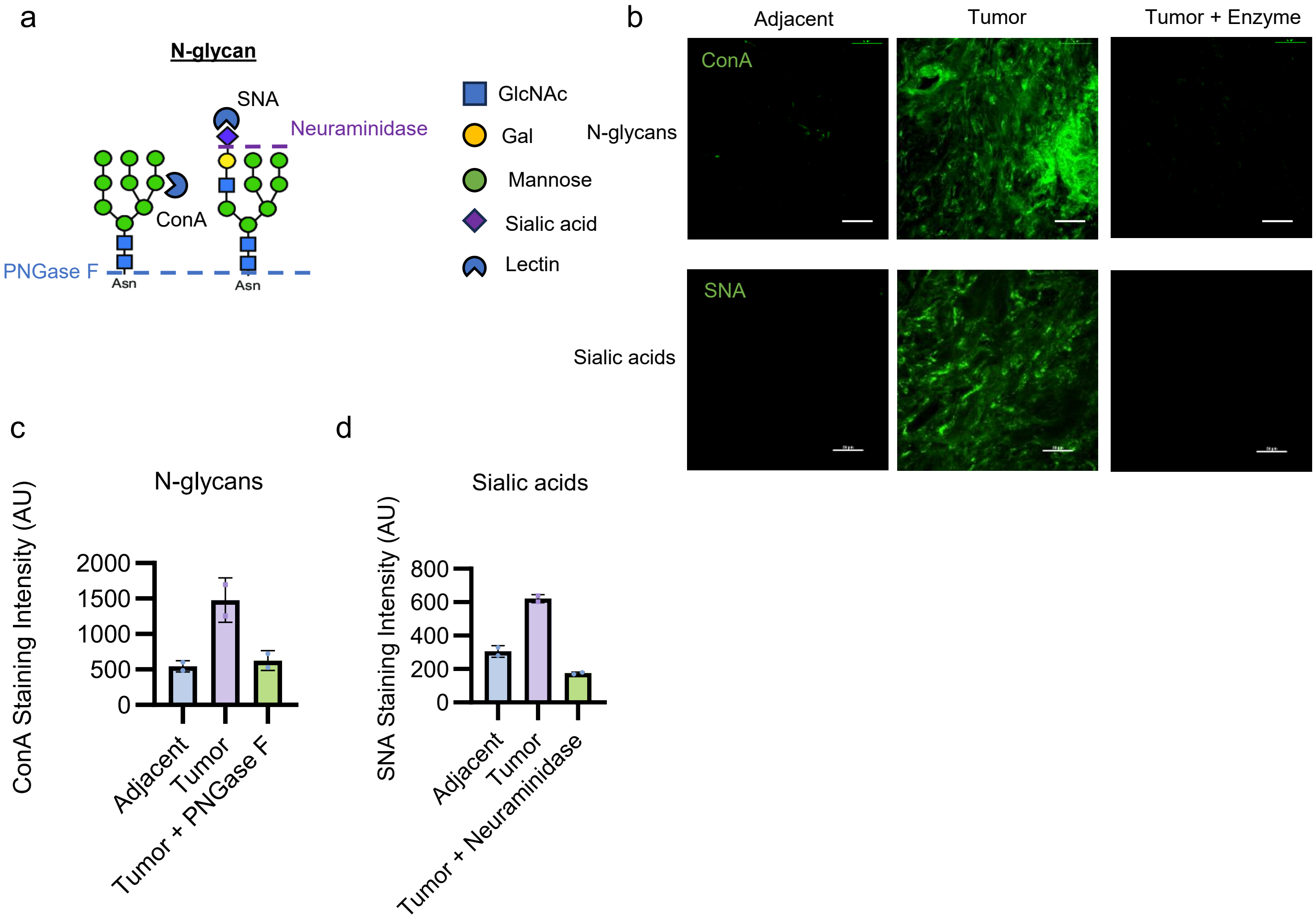

Supplementary Fig. 15: Lectin staining for N-linked and sialylated glycan structures in tumor and adjacent TNBC patient tissues and their cleavage using PNGase F and neuraminidase. a, Schematic of N-linked glycan enzymatic modifications and their respective lectin binding partners for staining. **b,** Representative images of adjacent, tumor, and tumor treated with PNGase F or neuraminidase. Staining with Concavalin A (ConA) lectin conjugated to AF488 for all N-linked glycans in the top row (green) and Sambucus nigra (SNA) lectin conjugated to AF488 for sialic acids in the bottom row (green). Scale bar = 100μm. **c-d,** Analysis of **c,** ConA-AF488 and **d,** SNA-AF488 lectin staining intensity using IMARIS image analysis software. N=2 matched tissue donors for each group, 5 FOV per tissue, One-way ANOVA with Tukey's post-hoc test**.**

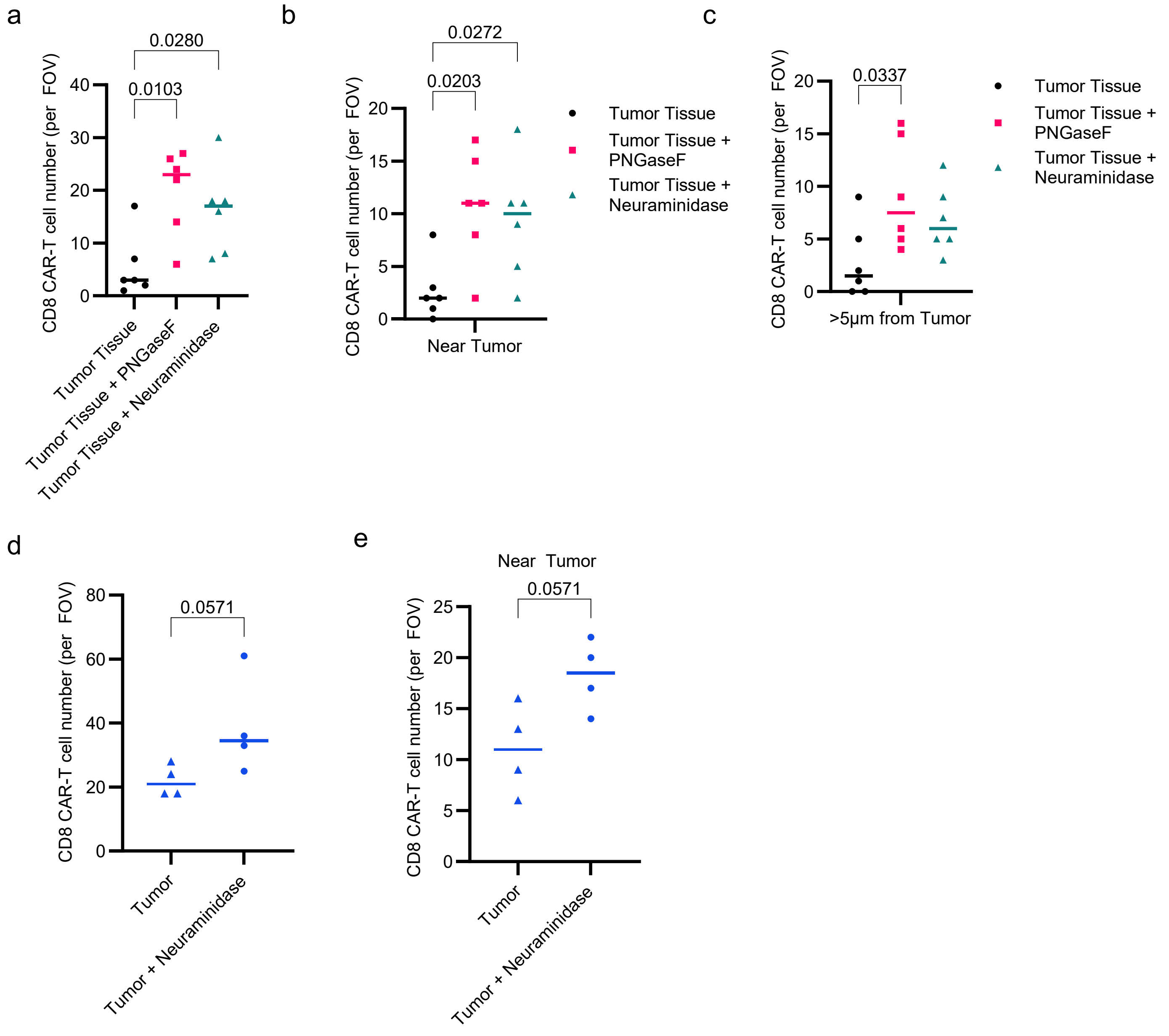

Supplementary Fig. 16: Decellularized tissue timelapse video analysis. Fluorescence-based live confocal timelapse imaging assay using decellularized tumor tissue treated with either PNGase F or neuraminidase enzyme treatment, seeded with BT20 TNBC cells and NKG2D-targeting CAR-T co-culture. Tissues stained with anti-human CD326 (EpCAM) AlexaFluor (AF) 488 (53-8326-42, 1:10) anti-human CD8-AF647 (344725, 1:10)) and anti-human fibronectin conjugated to Dylight 405 (F3648, 1:10). Video analysis performed using IMARIS analysis software. **a,** CD8+ CAR-T cell number per field of view (FOV). **b,** CD8+ CAR-T cell number per field of view (FOV) near EpCAM+ tumor cells (<5µm). **c,** CD8+ CAR-T cell number per field of view (FOV) >5µm from tumor cells. **a-c,** Line at median. One-way ANOVA with Holm-Šídák's multiple comparisons test. N=3, with 2 technical replicates each. **d-e,** MUC1-targeting CAR-T added to BT20-seeded decellularized tumor tissue. **d,** Mean CD8 MUC1-targeting CAR-T cell number per FOV. Line at median. Mann Whitney test. N=4. **e,** Mean CD8 MUC1-targeting CAR-T cell number near tumor (<5µm) per FOV. Line at median. Mann Whitney test. N=4.

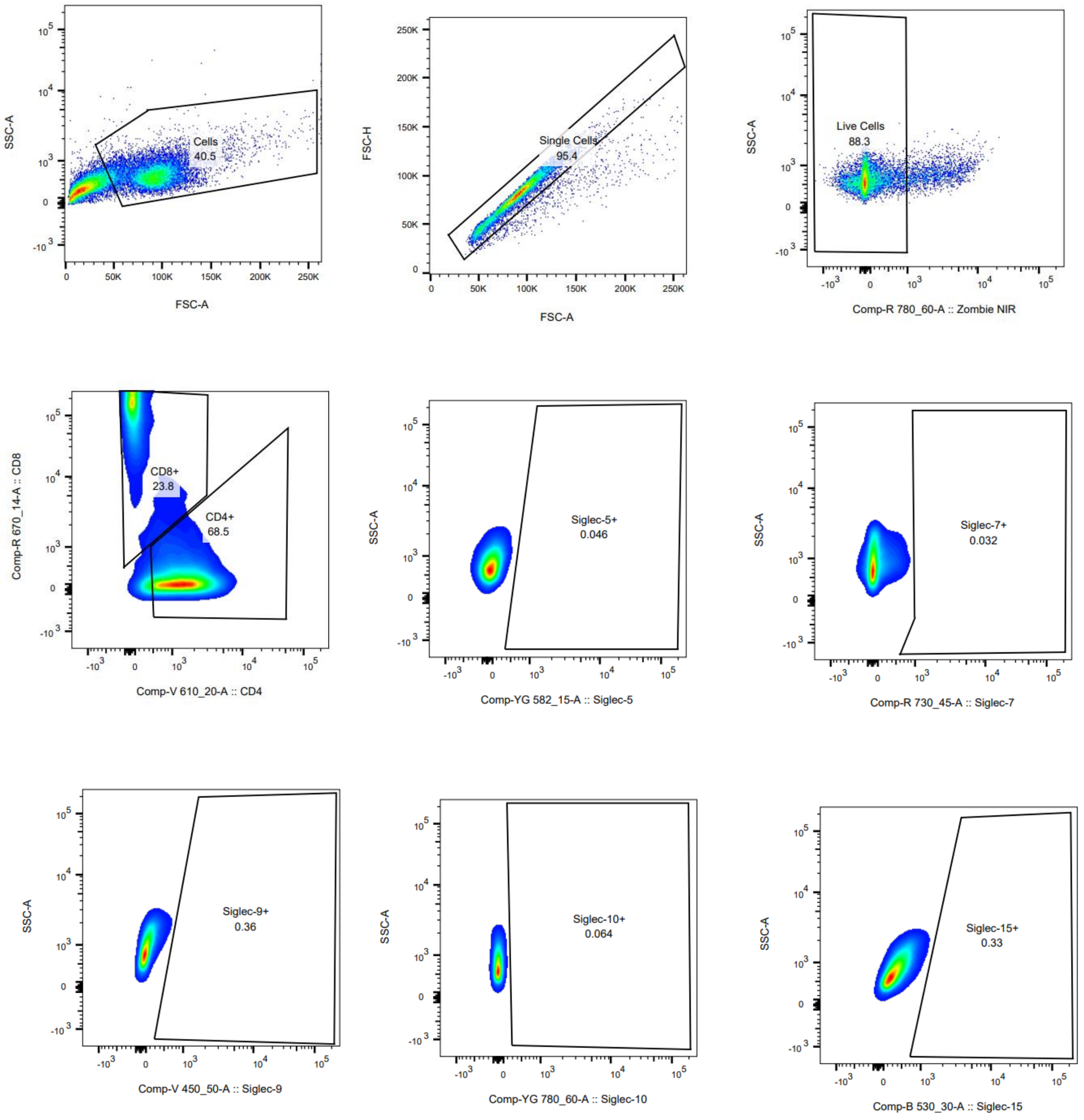

Supplementary Fig. 17: Flow gating strategy and fluorescence minus one (FMO) controls for CAR-T cell Siglec flow cytometry panel. CD4+ and CD8+ CAR-T cells assessed for Siglec expression using flow cytometry after five days. Cells were either cultured alone or co-cultured for five hours with BT20-seeded decellularized tumor tissue or tumor tissue treated with neuraminidase then transferred to a tissue culture plate for five days. N=3.

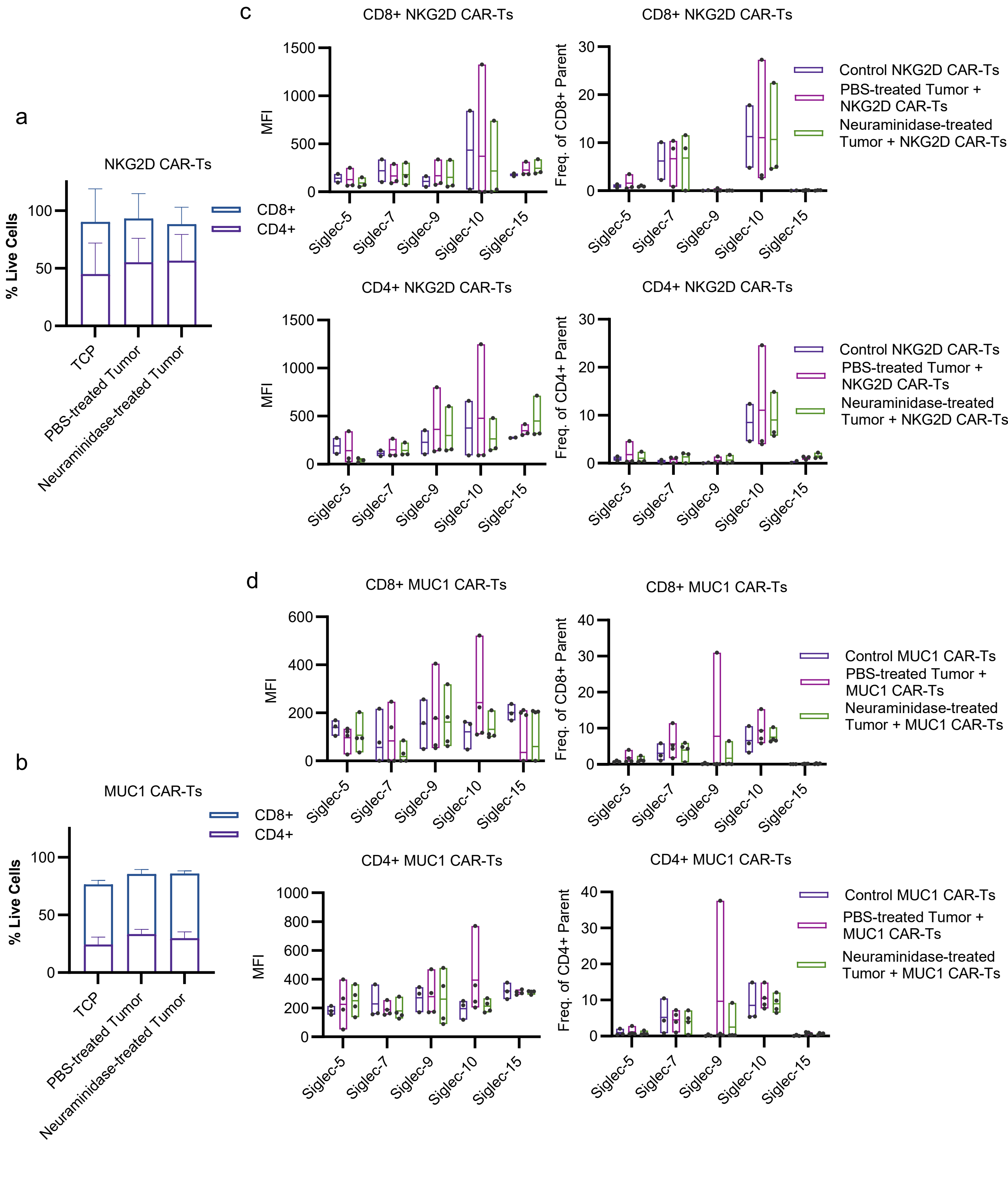

Supplementary Fig. 18: Neuraminidase treatment does not affect Siglec expression on CAR-Ts. a-b, Bar plots of flow cytometry expression patterns of CD8 and CD4 expression, shown as percentage of live cell population. **a,** NKG2D-targeting or **b,** MUC-1-targeting CAR-Ts cultured on tissue culture plastic (TCP), PBS-treated TNBC decellularized tumor tissue or neuraminidase-treated tumor tissue after five days. Mean with SD. Two-way ANOVA with Šídák's multiple comparisons test. N=3. **c-d,** Box plots of flow cytometry expression for a panel of Siglec receptors (Siglec-5, Siglec-7, Siglec-9, and Siglec-10) for **c,** NKG2D-targeting or **d,** MUC-1 targeting CAR-Ts. Line at median. Two-way ANOVA with Tukey's multiple comparisons test. N=2-4.

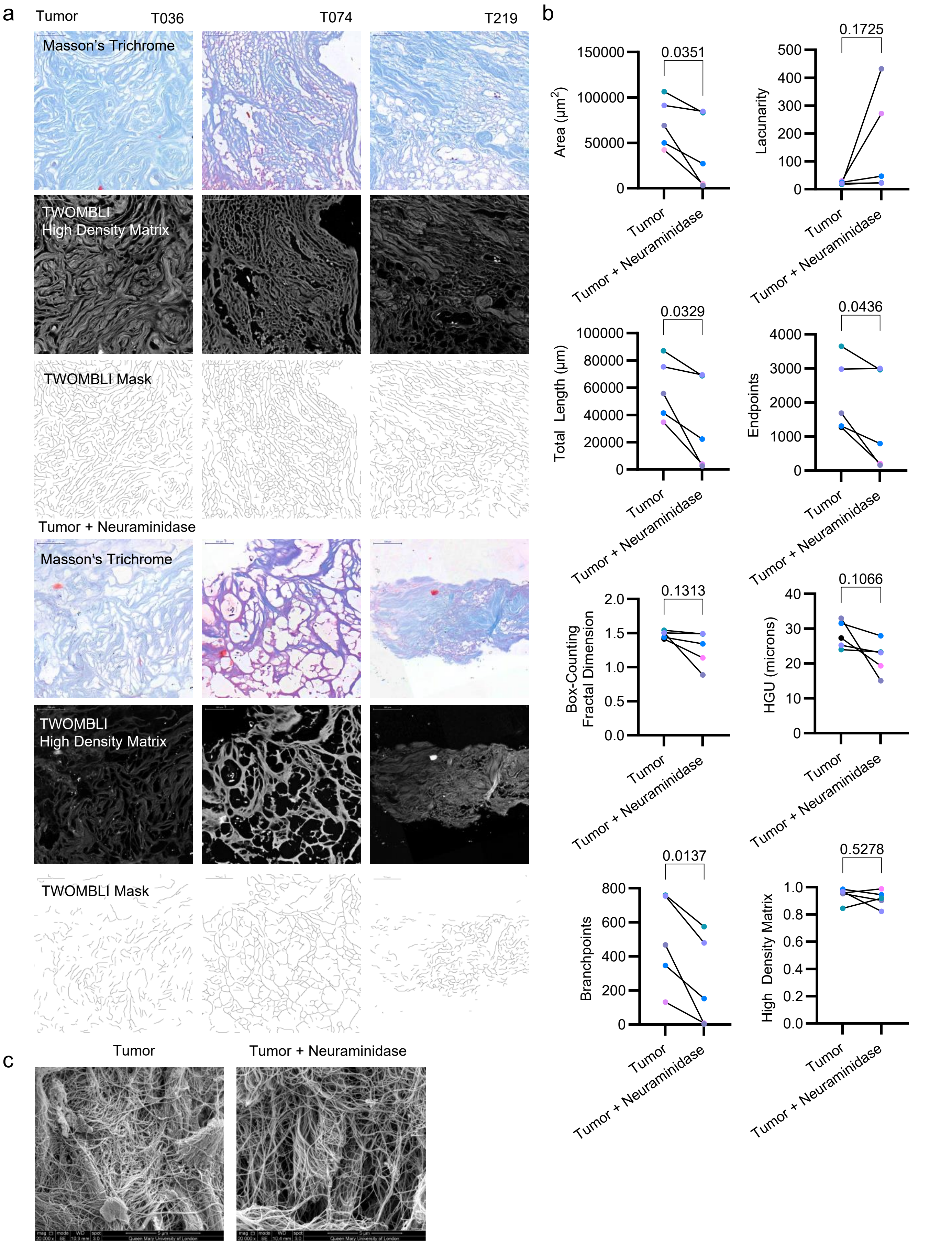

Supplementary Fig. 19: Glycan cleavage enzymes alter tissue architecture. a, Representative images of Masson's Trichrome staining & TWOMBLI analysis performed on matched tumor tissue treated with PBS or neuraminidase. Scale bar = 100μm. **b,** TWOMBLI analysis. Two-tailed paired t test. N=5 matched tumor & neuraminidase-treated tumor tissues. **c,** Representative scanning electron microscopy (SEM) images on matched tumor tissue treated with PBS or neuraminidase. Scale bar = 5μm.

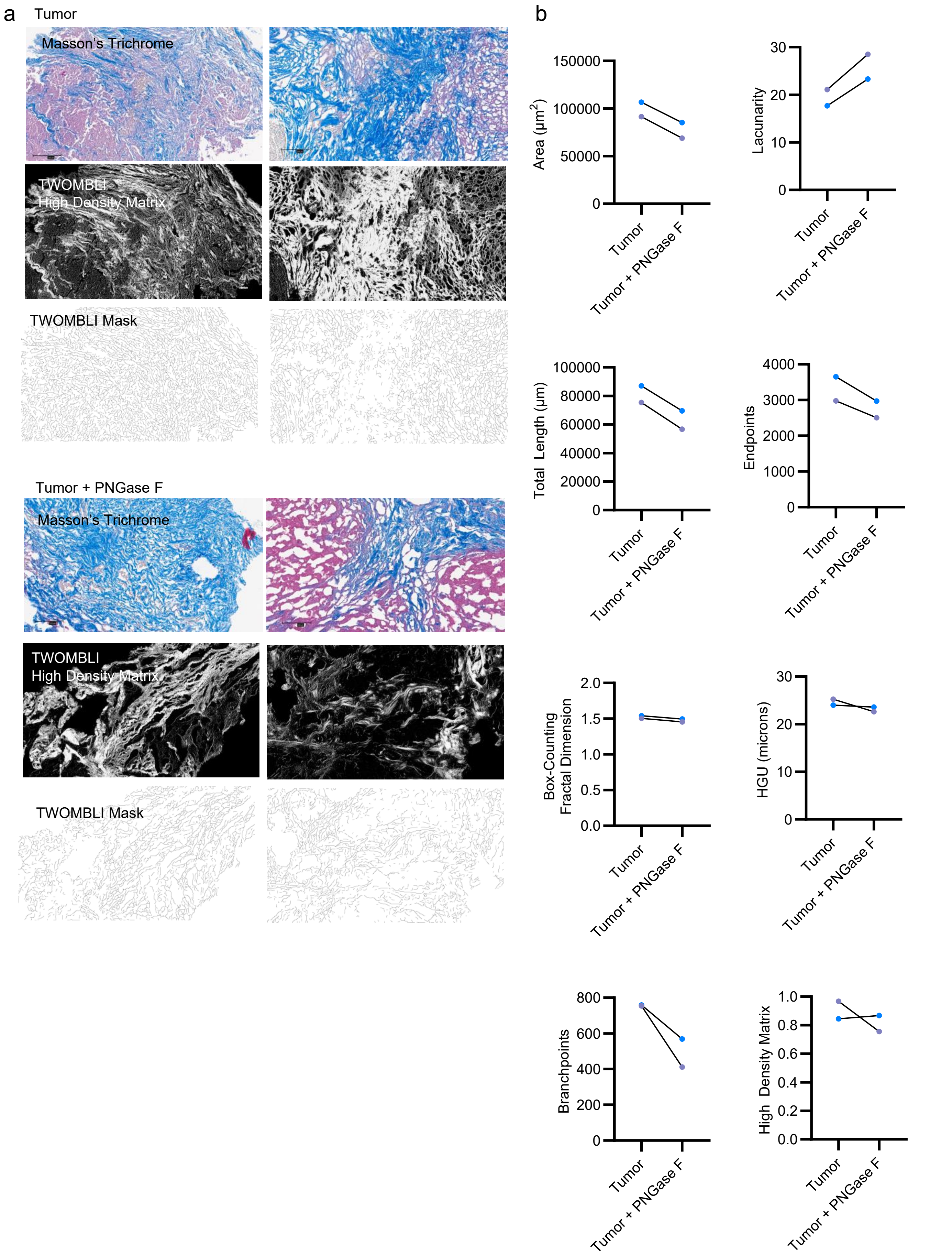

Supplementary Fig. 20: Glycan cleavage enzymes alter tissue architecture. a, Representative images of Masson's Trichrome staining & TWOMBLI analysis performed on matched tumor tissue treated with PBS or PNGase F. Scale bar = 100µm. **b,** TWOMBLI analysis. Two-tailed paired t test. N=2 matched tumor & PNGase F-treated tumor tissues.

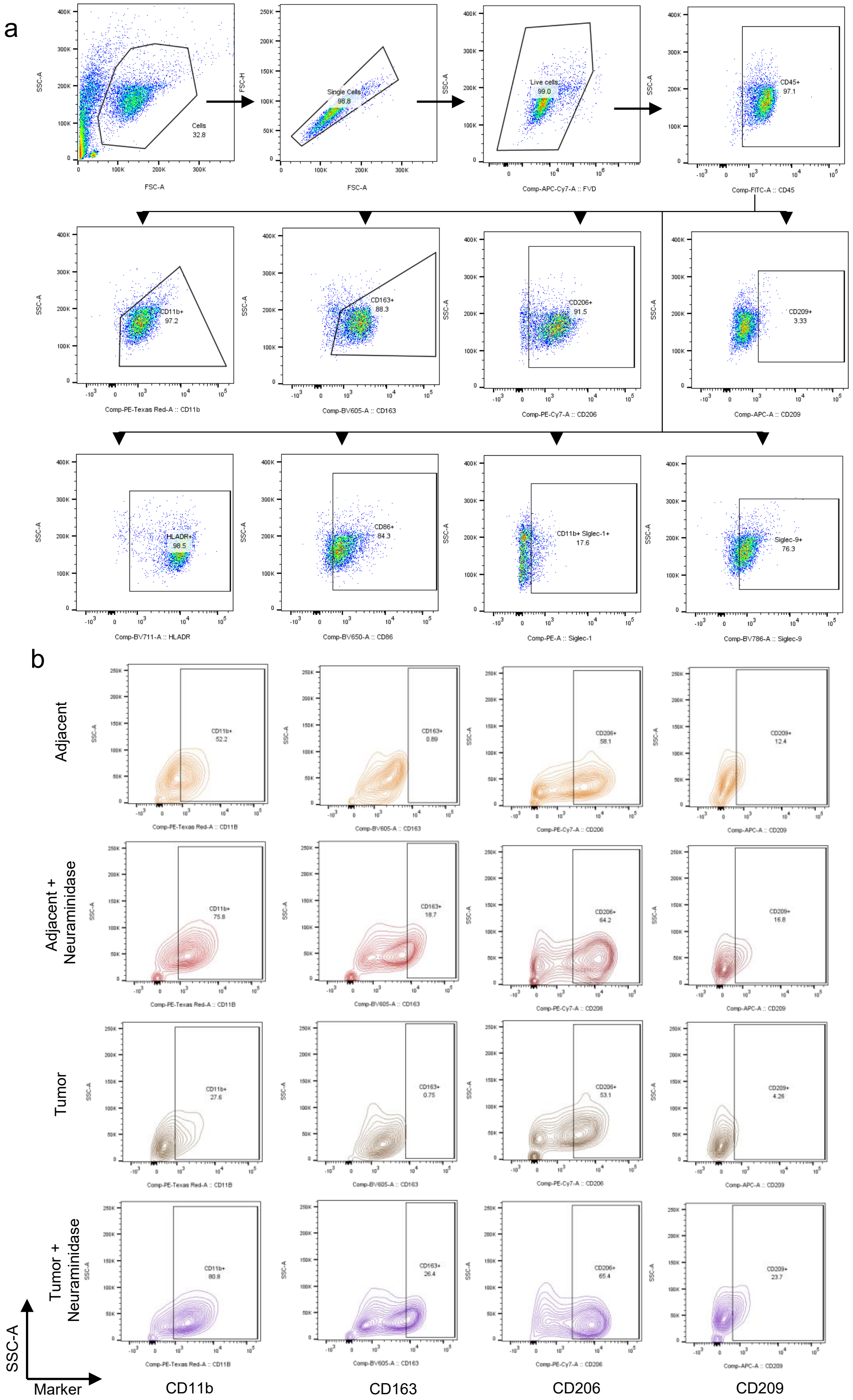

Supplementary Fig. 21. a, Flow gating strategy for macrophage flow cytometry assay. CD45+ macrophages and downstream phenotypic markers as assessed using flow cytometry after 14 days culture on adjacent or tumor tissue with or without neuraminidase treatment (Figure 6). N=3. **b**, Representative contour plots of CD11b+, CD163+, CD206+ and CD209+ macrophages cultured on adjacent or tumor tissues, with or without neuraminidase treatment. N=6 (N=6 adjacent, N=6 tumor, N=6 blood cones).

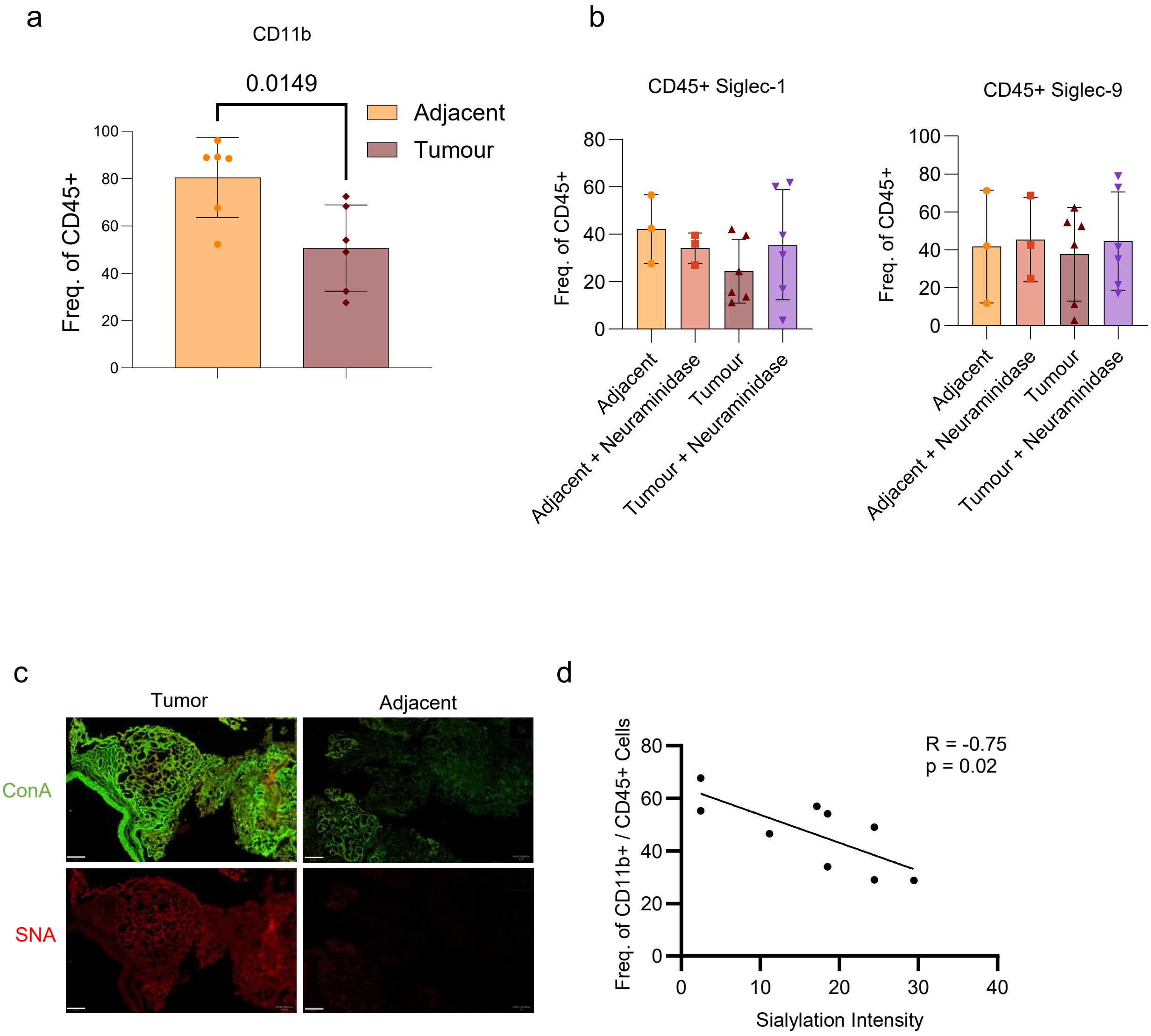

Supplementary Fig. 22. Macrophages educated on patient tissue express Siglec-1 and Siglec-9 and tissue sialylation correlated with CD11b+ macrophage population educated on matched tissues. a, Barchart of flow cytometry expression patterns of CD11b shown as percentage of positive cells from the CD45+ population. Line at mean with standard deviation error bars. Mixed-effects analysis with Tukey's multiple comparisons test. N=6 (N=6 adjacent, N=6 tumor, N=6 blood cones). **b,** Barchart of flow cytometry expression patterns of Siglec-1 and Siglec-9 shown as percentage positive cells from the CD45+ population. Line at mean with standard deviation error bars. Mixed-effects analysis with Tukey's multiple comparisons test. N=3 (N=2 adjacent, N=6 tumor, N=3 blood cones). **c,** Representative images of tumor and adjacent decellularized tissue stained with Concavalin A (ConA) lectin (green) for N-linked glycans, and Sambucus Nigra (SNA) lectin (red) for sialic acids. Scale bar = 100μm. **d,** Pearson's r values for correlation between SNA sialylation intensity of tissue and percentage positive cells from the CD45+ population educated on the tissue. N=9 tissues.

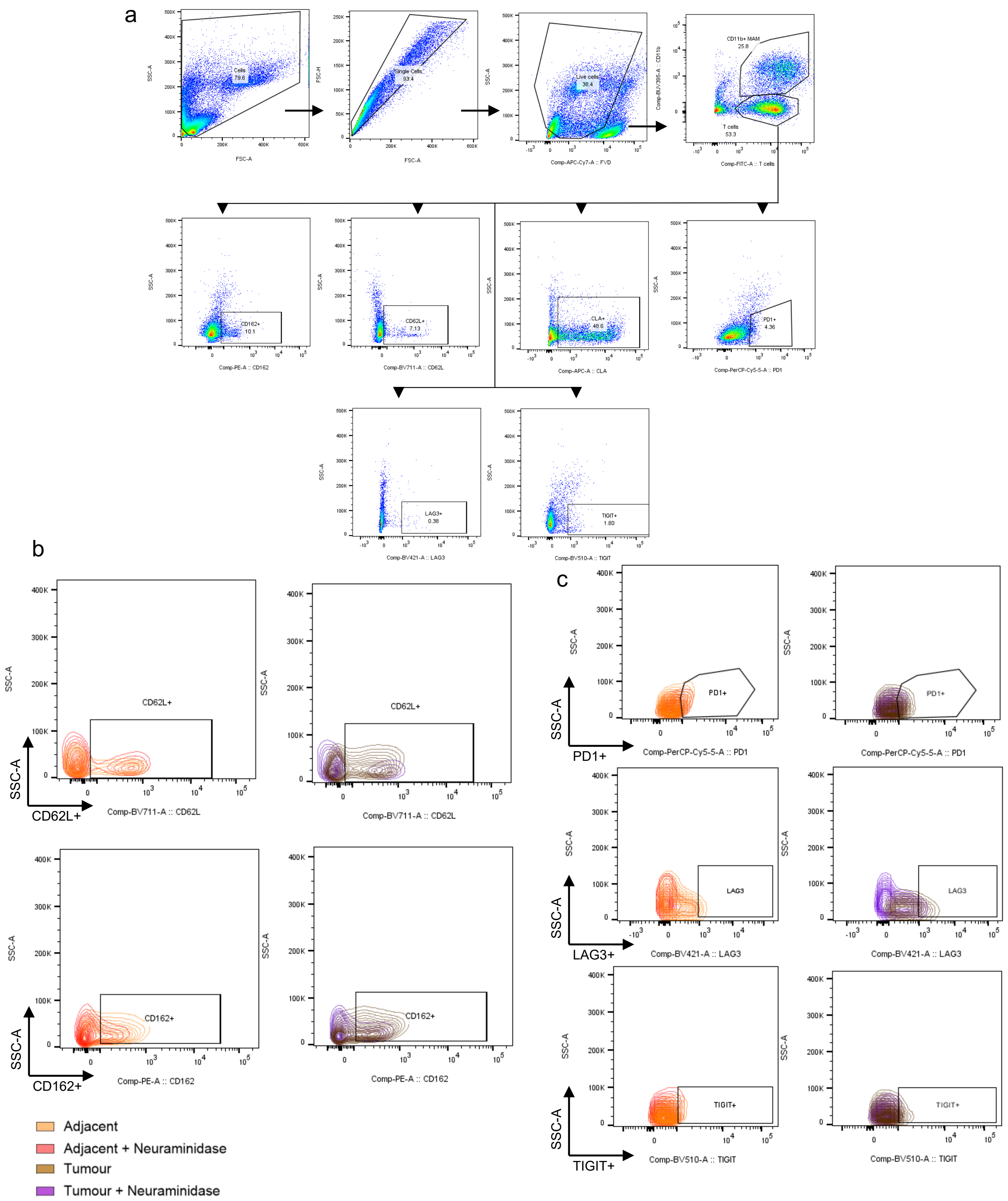

Supplementary Fig. 23. Flow gating strategy for T cell flow cytometry assay. a, T cells and downstream phenotypic markers as assessed using flow cytometry after 2 days in co-culture with MAMs (Figure 7). N=3. **b-c**, Representative contour plots of CD62L+, CD162+, PD1+, LAG3+, TIGIT+ T cells cultured on adjacent or tumor tissues with MAMs and cancer cell media, with or without neuraminidase treatment. N=3 (N=3 adjacent, N=3 tumor, N=3 blood cones).

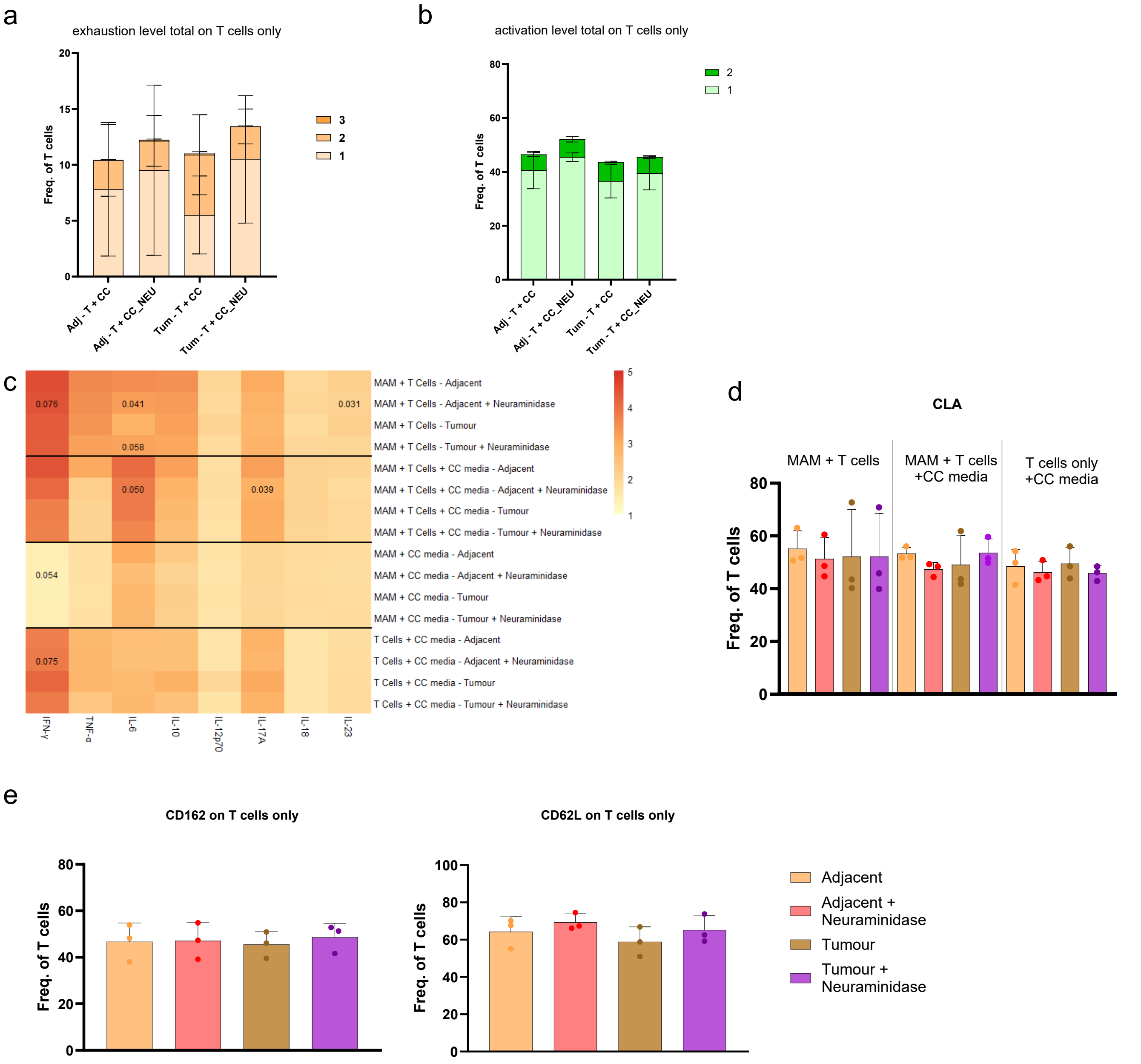

Supplementary Fig. 24. MAMs influence T cell phenotype in a glycan-dependent manner. Stacked bar charts of combinatorial gating for **a,** exhaustion markers (PD1, LAG3, TIM3) or **b,** activation markers (ICOS & CD137) shown as percentage positive cells expressing one marker, two markers or three markers from the T cell population. Line at mean with standard deviation error bars. Mixed-effects analysis with Tukey's multiple comparisons test. N=3 (N=3 adjacent, N=3 tumor, N=3 blood cones). **c,** Heatmap showing log10-transformed concentrations (pg/mL) of 8 cytokines (IFN-γ, TNF-α, IL-6, IL-10, IL-12p70, IL-17A, IL-18 and IL-23) measured in culture supernatants from macrophage (MAM) and T cell co-cultures (or monocultures) grown on decellularized adjacent or tumor ECM scaffolds, in the absence or presence of cancer cell–conditioned medium (CC). ECM scaffolds were either untreated or treated with neuraminidase (Neu) prior to cell seeding. N=3 (N=3 adjacent, N=3 tumor, N=3 blood cones). Overlaid numbers indicate paired t-test p-values for comparisons between untreated and neuraminidase-treated ECM within the same tissue and CC condition. **d-e,** Barchart of flow cytometry expression patterns of CLA, CD62L and CD162 on T cells shown as percentage of positive cells from T cell population. Line at mean with standard deviation error bars. Mixed-effects analysis with Tukey's multiple comparisons test. N=3 (N=3 adjacent, N=3 tumor, N=3 blood cones).

Marker	Secondary	Concentration	Catalog
panCK	Rabbit	1:1000	Z0622
CD8	Mouse	1:500	M7103
ConA-AF488	-	1:5	C11252
SNA-Cy3	-	1:100	CL-1303-1

Supplementary Table 1: Optimized antibodies for IHC.
