# Supplementary material for: Glycosylated extracellular matrix drives immune suppression by modulating macrophage-T cell crosstalk in triple-negative breast cancer
Source: Nat Commun. 2026 Jun 16;17:5008. doi: 10.1038/s41467-026-73467-5 (PMC13273098; doi:10.1038/s41467-026-73467-5)
Supplement: Supplementary file 3 — Reporting Summary [file 41467_2026_73467_MOESM3_ESM.pdf]

nature portfolio

Corresponding author(s): Oliver Pearce

Last updated by author(s): Apr 23, 2026

Reporting Summary

Nature Portfolio wishes to improve the reproducibility of the work that we publish. This form provides structure for consistency and transparency in reporting. For further information on Nature Portfolio policies, see our Editorial Policies and the Editorial Policy Checklist.

Statistics

For all statistical analyses, confirm that the following items are present in the figure legend, table legend, main text, or Methods section.

n/a	Confirmed	
[ ]	[x]	The exact sample size (*n*) for each experimental group/condition, given as a discrete number and unit of measurement
[ ]	[x]	A statement on whether measurements were taken from distinct samples or whether the same sample was measured repeatedly
[ ]	[x]	The statistical test(s) used AND whether they are one- or two-sided<br>*Only common tests should be described solely by name; describe more complex techniques in the Methods section.*
[x]	[ ]	A description of all covariates tested
[x]	[ ]	A description of any assumptions or corrections, such as tests of normality and adjustment for multiple comparisons
[ ]	[x]	A full description of the statistical parameters including central tendency (e.g. means) or other basic estimates (e.g. regression coefficient) AND variation (e.g. standard deviation) or associated estimates of uncertainty (e.g. confidence intervals)
[ ]	[x]	For null hypothesis testing, the test statistic (e.g. *F*, *t*, *r*) with confidence intervals, effect sizes, degrees of freedom and *P* value noted<br>*Give P values as exact values whenever suitable.*
[x]	[ ]	For Bayesian analysis, information on the choice of priors and Markov chain Monte Carlo settings
[x]	[ ]	For hierarchical and complex designs, identification of the appropriate level for tests and full reporting of outcomes
[ ]	[x]	Estimates of effect sizes (e.g. Cohen's *d*, Pearson's *r*), indicating how they were calculated

Our web collection on statistics for biologists contains articles on many of the points above.

Software and code

Policy information about availability of computer code

Data collection	RNA sequencing was performed at Oxford Genomics. Peptides were separated by nanoflow ultra-high pressure liquid chromatography (UPLC, NanoAcquity, Waters) and analyzed by mass spectrometry using a LTQ-Orbitrap XL mass spectrometer (Thermo Fisher Scientific). Glycan profiling was done on AB Sciex 4800 MALDI-TOF/TOF mass spectrometer.
Data analysis	All statistical analyses were performed using either GraphPad Prism software version 8.3.0 for Windows, GraphPad Software, San Diego, California, USA, www.graphpad.com or the statistical programming language RStudio (2022.02.3+492 "Prairie Trillium" Release) and R (version 4.1.1) using the following software plugins: Hmisc for correlation analysis, gplots for correlation scatter plots, ggplot2 for bar charts, pheatmap for heatmaps, dendextend for dendrograms and ggpubr for editing figures to publication standard. QuPath 0.5.11 or Definiens™ software was used for image analysis of the TNBC tissues. Imaris 9.1 (Oxford Instruments, Bitplane) was used for analysis of CAR-T movement and location within the tissue slice.

For manuscripts utilizing custom algorithms or software that are central to the research but not yet described in published literature, software must be made available to editors and reviewers. We strongly encourage code deposition in a community repository (e.g. GitHub). See the Nature Portfolio guidelines for submitting code & software for further information.

Data

Policy information about availability of data

All manuscripts must include a data availability statement. This statement should provide the following information, where applicable:

- Accession codes, unique identifiers, or web links for publicly available datasets
- A description of any restrictions on data availability
- For clinical datasets or third party data, please ensure that the statement adheres to our policy

The RNAseq data have been deposited in NCBI's Gene Expression Omnibus under the accession number GSE318780. The mass spectrometry proteomics data have been deposited to the ProteomeXchange Consortium via the PRIDE partner repository under the dataset identifier PXD075558. Glycomics data was uploaded to Zenodo 10.5281/zenodo.18595849.

Research involving human participants, their data, or biological material

Policy information about studies with human participants or human data. See also policy information about sex, gender (identity/presentation), and sexual orientation and race, ethnicity and racism.

Reporting on sex and gender	only tissue samples from females were used in this work.
Reporting on race, ethnicity, or other socially relevant groupings	race, ethnicity or other socially relevant groupings were not reported in our work.
Population characteristics	patient age is recorded within supplemental table 6
Recruitment	n/a study based on existing samples.
Ethics oversight	All human female TNBC tissue was obtained from the Breast Cancer Now (BCN) tissue bank (REC 21/EE/0072). Each patient gave written informed consent. The work was conducted in accordance with the Declaration of Helsinki and International Ethical Guidelines for Biomedical Research Involving Human Subjects (CIOMS).

Note that full information on the approval of the study protocol must also be provided in the manuscript.

Field-specific reporting

Please select the one below that is the best fit for your research. If you are not sure, read the appropriate sections before making your selection.

☒ Life sciences ☐ Behavioural & social sciences ☐ Ecological, evolutionary & environmental sciences

For a reference copy of the document with all sections, see nature.com/documents/nr-reporting-summary-flat.pdf

Life sciences study design

All studies must disclose on these points even when the disclosure is negative.

Sample size	*Describe how sample size was determined, detailing any statistical methods used to predetermine sample size OR if no sample-size calculation was performed, describe how sample sizes were chosen and provide a rationale for why these sample sizes are sufficient.*
Data exclusions	*Describe any data exclusions. If no data were excluded from the analyses, state so OR if data were excluded, describe the exclusions and the rationale behind them, indicating whether exclusion criteria were pre-established.*
Replication	*Describe the measures taken to verify the reproducibility of the experimental findings. If all attempts at replication were successful, confirm this OR if there are any findings that were not replicated or cannot be reproduced, note this and describe why.*
Randomization	*Describe how samples/organisms/participants were allocated into experimental groups. If allocation was not random, describe how covariates were controlled OR if this is not relevant to your study, explain why.*
Blinding	*Describe whether the investigators were blinded to group allocation during data collection and/or analysis. If blinding was not possible, describe why OR explain why blinding was not relevant to your study.*

Reporting for specific materials, systems and methods

We require information from authors about some types of materials, experimental systems and methods used in many studies. Here, indicate whether each material, system or method listed is relevant to your study. If you are not sure if a list item applies to your research, read the appropriate section before selecting a response.

Materials & experimental systems

n/a	Involved in the study	
	x	Antibodies
	x	Eukaryotic cell lines
x		Palaeontology and archaeology
x		Animals and other organisms
x		Clinical data
x		Dual use research of concern
x		Plants

Methods

n/a	Involved in the study	
x		ChIP-seq
	x	Flow cytometry
x		MRI-based neuroimaging

Antibodies

Antibodies used

The following antibodies were used for immunohistochemical analyses: panCK (1:1000, Dako: Z0622), CD8 Mouse (1:500, Dako: M7103), fibronectin (FN1) (1:500, Abcam: ab23750), versican (VCAN) (1:200, ATLAS: HPA004726), collagen 1A1 (COL1A1) (1:300, ATLAS: HPA011795), chondroitin sulphate (CS) (1:600, Abcam: ab11570), cathepsin B (CTSB (1:400, Abcam: ab58802), cartilage oligomeric matrix protein (COMP) (1:80, Abcam: ab11056). The following antibodies were used for CAR-T flow cytometry analyses: anti-human CD233 (LAG-3) (BV421, 1:100, Cat: 303415, Biolegend), CD4 (BV605, 1:250, Cat:, Biolegend), CD8 (AF647, 1:100, Cat: 344725, Biolegend), Siglec-5 (PE, 1:100, Cat: 352003, Biolegend), Siglec-7 (AF700, 1:100, Cat: 339209, Biolegend), Siglec-9 (BV421, 1:100, Cat: 743363, BD Biosciences), Siglec-10 (PE Cy7, 1:100, Cat: 347607, Biolegend), Siglec-15 (AF488, 1:100, Cat: FAB9227G, R&D Systems). The following antibodies were used for monocyte-derived macrophage flow cytometry analyses: CD45 (FITC, 1:100, Cat: 368508, Biolegend), CD36 (BV421, 1:100, Cat: 336229, Biolegend), CD163 (BV605, 1:100, Cat: 333616, Biolegend), CD86 (BV650, 1:100, Cat: 105035, Biolegend), HLADR (BV711, 1:100, Cat: 307643, Biolegend), Siglec-9 (BV786, 1:100, Cat: 743366, BD Bioscience), CD209 (APC, 1:100, Cat: 330107, Biolegend), SIRpa (AF700, 1:100, Cat: 323816, Biolegend), Siglec-1 (PE, 1:100, Cat: 346003, Biolegend), CD11b (AF594, 1:100, Cat: 301340, Biolegend), CD206 (PE-Cy7, 1:100, Cat: 321123, Biolegend). The following antibodies were used for PBMC T cell and monocyte-derived macrophage flow cytometry analyses: PD1 (PerCP, 1:100, Cat: 329937, Biolegend), CD206 (AF700, 1:100, Cat: 321132, Biolegend), CLA (AF647, 1:100, Cat: 321310, Biolegend), TIM3 (BUV805, 1:100, Cat: 368-3109-42, ThermoFisher), CD8 (BUV563, 1:100, Cat: 612915, BD Bioscience), CD11b (SparkUV 387, 1:100, Cat: 301366, Biolegend), ICOS (BV785, 1:100, Cat: 313533, Biolegend), CD62L (BV711, 1:100, Cat: 304860, Biolegend), Siglec-9 (BV650, 1:100, Cat: 743366, BD Bioscience), CD163 (BV605, 1:100, Cat: 333616, Biolegend), TIGIT (BV510, 1:100, Cat: 372737, Biolegend), LAG3 (BV421, 1:100, Cat: 369314, Biolegend), CD209 (PE-Cy7, 1:100, Cat: 330114, Biolegend), CD162 (Pe, 1:100, Cat: 328806, Biolegend).

Validation

All antibodies were validated for their use in the application and species according to manufacturer's websites. IHC antibodies were validated as compared to an isotype control. Flow antibodies were validated by titration.

Eukaryotic cell lines

Policy information about cell lines and Sex and Gender in Research

Cell line source(s)

BT20 and HCC38 (female triple negative breast cancer cell lines)

Authentication

All cell lines were authenticated by short tandem repeat profiling using the authentication service of the American Type Culture Collection (FTA Sample Collection kits).

Mycoplasma contamination

All cell lines were routinely tested for mycoplasma contamination (Lonza)

Commonly misidentified lines
(See ICLAC register)

Name any commonly misidentified cell lines used in the study and provide a rationale for their use.

Plants

Seed stocks

Report on the source of all seed stocks or other plant material used. If applicable, state the seed stock centre and catalogue number. If plant specimens were collected from the field, describe the collection location, date and sampling procedures.

Novel plant genotypes

Describe the methods by which all novel plant genotypes were produced. This includes those generated by transgenic approaches, gene editing, chemical/radiation-based mutagenesis and hybridization. For transgenic lines, describe the transformation method, the number of independent lines analyzed and the generation upon which experiments were performed. For gene-edited lines, describe the editor used, the endogenous sequence targeted for editing, the targeting guide RNA sequence (if applicable) and how the editor was applied.

Authentication

Describe any authentication procedures for each seed stock used or novel genotype generated. Describe any experiments used to assess the effect of a mutation and, where applicable, how potential secondary effects (e.g. second site T-DNA insertions, mosiacism, off-target gene editing) were examined.

Flow Cytometry

Plots

Confirm that:

- [x] The axis labels state the marker and fluorochrome used (e.g. CD4-FITC).
- [x] The axis scales are clearly visible. Include numbers along axes only for bottom left plot of group (a 'group' is an analysis of identical markers).
- [x] All plots are contour plots with outliers or pseudocolor plots.
- [x] A numerical value for number of cells or percentage (with statistics) is provided.

Methodology

Sample preparation	*At the end of the 14 days, tissues were dissociated with 1 mg/ml Liberase and T cells and macrophages collected for flow cytometry staining.*
Instrument	Samples were acquired on either a LSR Fortessa II (BD Bioscience) or a BD Symphony A3 1 (BD Bioscience).
Software	Data was analysed using FlowJo v10.8.1 (BD FlowJo LLC).
Cell population abundance	*Cell population abudance: 50,000 cells per sample*
Gating strategy	*For Macrophages only experiments: Cells > Single cells > Live cells > CD45+ > follow up markers (CD11b, CD206, CD163, CD209, Siglec-1, Siglec-9).*<br>*For MAM+T cells: Cells > Single cells> Live cells> FITC tracked T cells> follow up markers (CD162, CD62L, CLA, TIGIT, PD1, LAG3, ICOS, CD137)*

- [x] Tick this box to confirm that a figure exemplifying the gating strategy is provided in the Supplementary Information.
