# Supplementary material for: Glycosylated extracellular matrix drives immune suppression by modulating macrophage-T cell crosstalk in triple-negative breast cancer
Source: Nat Commun. 2026 Jun 16;17:5008. doi: 10.1038/s41467-026-73467-5 (PMC13273098; doi:10.1038/s41467-026-73467-5)
Supplement: Supplementary file 4 — Description of Additional Supplementary Files [file 41467_2026_73467_MOESM4_ESM.pdf]

Supplementary Data Legends:

- **Supplementary Data 1:** RNA-seq dataset of TNBC tissues. (gene expression)
- **Supplementary Data 2:** ECM-enriched proteomics dataset of tumor and adjacent tissues. (proteomics dataset)
- **Supplementary Data 3:** N-linked glycomics profiling of tumor and adjacent tissues.
- **Supplementary Data 4:** Differentially expressed ECM-related genes identified between tumor and adjacent tissues.
- **Supplementary Data 5:** Differentially abundant ECM proteins identified between tumor and adjacent tissues.
- **Supplementary Data 6:** Patients characteristics
