# Supplementary material for: Glycosylated extracellular matrix drives immune suppression by modulating macrophage-T cell crosstalk in triple-negative breast cancer
Source: Nat Commun. 2026 Jun 16;17:5008. doi: 10.1038/s41467-026-73467-5 (PMC13273098; doi:10.1038/s41467-026-73467-5)
Supplement: Supplementary file 2 — Transparent Peer Review file [file 41467_2026_73467_MOESM2_ESM.pdf]

nature portfolio

Peer Review File

Glycosylated extracellular matrix drives immune suppression by modulating macrophage-T cell crosstalk in triple-negative breast cancer

Corresponding Author: Dr Oliver Pearce

This file contains all reviewer reports in order by version, followed by all author rebuttals in order by version.

Version 0:

Reviewer comments:

Reviewer #1

(Remarks to the Author)
In this manuscript, the authors described a novel role for extracellular matrix (ECM) glycosylation in promoting immune suppression in triple negative breast cancer (TNBC).
The authors provided a detailed characterisation of decellularised ECM from TNBC tumours and adjacent tissues, at the mRNA, protein and glycosylation levels. This indicated significant changes both at the protein levels, as well as changing the the glycosilation of ECM components. In addition, they showed that tumour ECM prevented T cell infiltration, was linked to epithelial to mesenchymal transition (EMT) in tumour cells and affected macrophage differentiation.
Enzymatic removal of glycosylation promoted anti-tumour immunity, suggesting this might represent a novel therapeutic strategy.

This study is rigorous and well written, however some aspects appear quite preliminary and further work is need to strengthen the conclusions and provide more mechanistic information.

1. The decellularisation protocol is described in detail. The authors show in figure 4d that this results in a significant increase in adjacent ECM stiffness (but not tumour). Why is this? Is this affecting the cellular responses to this matrix?

2. The authors perfomed live/dead staining of cancer cells seeded on collagen, tumour or adjacent decellularised ECM in both figure 4f,g and figure 5a,b. The confluency of the cells appears very different at day 1 across the conditions tested in figure 4 (and the quantification is only N=2). HCC38 seemed to be progressively lost from all the ECM conditions in figure 4, but not in figure 5, while there seem to be a lot more dead cells in figure 5 compared to figure 4. Can the authors clarify these differences or provide additional repeats to strengthen these observations?

3. The EMT data feel very preliminary (some are N=1). There appears to be cell-line dependent changes in gene expression. Some changes highlighted in the text (such as the increase in collagen I expression in cancer cells), are not statistically significant when comparing tumour versus adjacent ECM. The phenotype observed seems more partial EMT. I think the authors should characterise in more detail the expression of different EMT markers, including both epithelial and mesenchymal ones, both at the mRNA and protein levels. Once a more detailed characterisation is provided, the effect of glycosylation removal should be tested in both cell lines.

4. When assessing T cell infiltration in vitro (figure 6), why only BT-20 cells were used? The quantifications in c-f are a bit difficult to follow. Why were the data not combined in a single graph showing cells near and far from tumours, in both tumour and adjacent ECM?
In the representative images shown, it seems that treatment with PNGase F resulted in much bigger tumour islets, compared to the other conditions. Since the authors showed that changes in the T cell movement/infiltration required the presence of cancer cells, here it is crucial to determine how glycosylation removal affects the formation of cancer cell islets.

Could changes in T cells infiltration be linked to EMT in cancer cells? EMT has been shown to reduce T cell infiltration in lung cancer (PMID: 29440769), therefore it'd be interesting to investigate whether the same is happening here.

5. The live cell imaging results are not very convincing, as the graphs show minimal differences in speed (despite the extremely high statistical significance)

6. Removal of glycosylation resulted in profound changes to the overall ECM architecture, as expected as glycosylation is required for the maintenance of ECM networks. It will be important to determine whether the changes observed here are due to physical alterations of the ECM, or changes in cell/ECM interactions, which are also modulated by glycosylation levels.

7. Macrophage differentiation: only CD45+ cell frequency is reported to be significantly different between tumour and adjacent ECM, while all the other markers were not changed. I think if is essential to better define the differences between tumour and adjacent ECM, before determining the role of glycosylation. In the data provide in the manuscript, the removal of glycosylation mostly has the same effect on tumour and adjacent ECM - why is this? Could this be driven by changes in overall ECM architecture, rather than being specific for ECM glycosylation in tumours?

Reviewer #2

(Remarks to the Author)
The manuscript by Tyler, Liu and Tarantola et al., entitled “Glycosylated extracellular matrix drives immune suppression by controlling T-cell movement, macrophage phenotype, and macrophage–T-cell crosstalk in triple-negative breast cancer,” provides relevant and novel findings linking alterations in ECM glycosylation, particularly N-glycan sialylation, to immune exclusion. This aligns with and expands beyond previous work on ECM/matrisome signatures and proteoglycans, further supporting that changes in ECM components critically influence T-cell trafficking and macrophage programming. Building on previous findings, the authors started by exploiting a TCGA matrix-index analysis to demonstrate links between alterations in ECM components linked to glycosylation and immune exclusion. They further explore their own patient samples employing bulk RNA-seq, ECM-enriched proteomics, MALDI-TOF glycomics, lectin histology, and human decellularized-tissue models re-cellularized with TNBC cells. They show that tumor ECM compared with matched adjacent/surround tissue display increased sialylation, fucosylation and poly-LacNAc on N-glycans. Among these features, elevated sialylation appears to better stratify immune-excluded tumors. Enzymatic removal of N-glycans and/or terminal sialic acids increased $CD8^+$ CAR-T infiltration and motility, reduced EMT-associated programs in tumor cells, and reprogramed macrophages toward a more activated phenotype. This reprogramming has been shown to modulate macrophage–T-cell crosstalk. This behavior was not observed for adjacent/surround ECM, supporting a cancer-dependent effect. While mechanistic aspect are not fully resolved and in vivo validation remains to be accomplished, the data supports that targeting and rewiring ECM glycosylation may provide a path to convert immune-excluded TNBC into a more inflamed state. Overall, the methodological approach is appropriate and generally adequate to support these conclusions. The manuscript is well organized and easy to follow. The observations are novel and meaningful to better understand the intricate crosstalk between the ECM and the immune system, with implications for cancer progression.

The findings are relevant and may hold translational implications. However, some concerns persist, as follows:

1. The TCGA analysis is based on all invasive breast cancers. On the other hand, experimental work is focused on TNBC. To strengthen clinical relevance, the MI–survival and MI–pathway enrichment could be addressed, if possible, specifically within TNBC cases. This would better align with the mechanistic focus of the study.

2. When addressing patient samples, the manuscript introduces an inflamed/excluded/desert stratification of tumors based on T-cell infiltration. However, this classification was not fully and consistently integrated into the subsequent analyses. The narrative would be significantly strengthened if the authors could explicitly connect these immune classes to the ECM glycosylation features and to the MI framework introduced in the beginning of the work across the manuscript. Linking these features to functional implications would significantly broaden the translational character of the findings, providing grounds for future decision-making.

3. Glycomics data was obtained for ECM but also membrane fractions and whole cells. However, it is not always clear how well these fractions are separated and the level of contamination between compartments, which may influence the observed profiles. Therefore, the authors should clarify the fractionation workflow, purity checks, and control strategy.

4. The glycan data is reported as relative percentages of permethylated glycans identified by MALDI-TOF. Because percentages are interdependent (if one glycan goes up, others go down), ANOVA analysis may not be adequate for data interpretation and capturing real differences. Also, data normalization of complementary quantitative methods, for instance LC-MS/MS with standards, would provide more unequivocal information.

5. Glycomics datasets are imbalanced, with large tumor cohorts contrasting with few adjacent controls. The limited representation of adjacent tissue diminuishes the robustness of subsequent comparisons. Furthermore, it raises some concerns that the reported differences could be driven by individual variability. Expanding the number of adjacent ECM samples would significantly strengthen the conclusions.

6. Following MS-assisted glycomics, the manuscript presents a broad lectin survey of ECM glycosylation (MAL-II, VVA, UEA-I, and others) that supports glycome remodeling. However, in the functional assays the focus narrows almost exclusively to SNA (α2,6-sialylation) and ConA (mannose-binding). Other apparently relevant lectins are not revisited. There also seems to be a shift from MAL-II (α2,3-sialylation) in the initial survey to SNA (α2,6-sialylation) in functional studies. This raises doubts about the actual sialylation changes which are most relevant. It would be important to explain why α2,6-sialylation was prioritized, especially after initially emphasizing α2,3 sialylation. Furthermore, it would be important to

address if a broader lectin signals also change under functional conditions.

7. Decellularize ECM are repopulated with cancer cell models to address the functional impact of the matrix at multiple levels. Please clarify whether the two used TNBC lines (BT20 and HCC38) differ in baseline glycosylation and/or ECM-remodeling profiles and if their differences are relevant for the observed outcomes. This may provide a more comprehensive picture on the observed crosstalk.

8. To me it was rather difficult to identify which TNBC line was used for each assay. As it reads, most CAR-T infiltration and enzyme-rescue experiments appear to rely on BT20, whereas HCC38 is mainly presented for ECM phenotyping. If this is the case, it would be important to replicate key findings with both lines to support generalizability across TNBC models. It should also be made explicit that macrophage education experiments are performed directly on patient ECM without tumor cells, which carries distinct implications for interpreting cell–ECM crosstalk. Notably, performing these assays in the presence of cancer cells, even though not mandatory, would strength the current observations.

9. The manuscript introduces MDA-MB-468 cells for the 2D CAR-T cytotoxicity and cytokine release assays without providing context. Please clarify the rationale for including this additional line, and why the cell line was not considered earlier.

10. The authors show that Siglec-7 and Siglec-10 levels on T cells do not change after ECM desialylation. Based on this observation the authors argue against Siglec signaling. However, what actually changes in these assays is the availability of sialylated ligands in the ECM. The fact that receptor expression stays constant does not necessarily mean the receptors are not involved. In fact, if there are fewer ligands to engage with, the inhibitory signaling should go down, which fits quite well with the increased T-cell infiltration seen after neuraminidase treatment. Nevertheless, to really pin down the mechanism, the author could directly test ligand–receptor interactions and dependence. For example, checking Siglec-Fc binding to ECM before and after desialylation, blocking Siglecs with antibodies or sialic acid competitors, or knocking out siglecs in T cells and macrophages (which is rather hard to do) would provide a much clearer answer. Also, knowing exactly the nature of the sialic acids involved could provide a more educated notion about the siglecs or other immune ligands involved. This reinforces the need for more informative glycomics.

11. Building on the previous comment, the manuscript repeatedly refers to “ECM hyperglycosylation” as a barrier to immune infiltration. Notably the authors mention increased sialylation and fucosylation of N-glycans. However, the nature of the fucosylation, particularly core vs outer branching, and the type of sialylation (α2,3 vs α2,6) are not fully disclosed. This distinction matters, since different Siglec receptors exhibit divergent binding preferences. To strengthen the mechanistic claim, the authors should clarify which sialylation type is predominantly enriched in tumor ECM and, if possible, align this with Siglec-binding specificities. Exploiting both MAL and SNA lectin and linkage specific sialidases could perhaps help clarify this aspect.

12. The manuscript also reports increased fucosylation of N-glycans, which is an interesting finding. However, the specific nature of this change remains unresolved, and this distinction could have very different implications at multiple levels. I understand that the MALDI-based workflow provides only compositional information without resolving linkage or positional details, and I acknowledge that the authors have been careful not to overinterpret their data. Nevertheless, only UEA-I was included in the initial lectin panel, which does not address functionally relevant core or Lewis-type fucosylation. Complementary use of more informative probes or MS/MS-based structural characterization would substantially strengthen the mechanistic interpretation.

13. In the final set of experiments, the authors analyze typical T-cell activation/exhaustion and macrophage lineage/polarization markers after culture on tumor versus adjacent ECM with a with sialidase. While these experiments are very informative, the absence of cytokine profiling limits interpretation of the functional consequences. Assessing them would provide a more complete picture of immune modulation and strengthen the mechanistic link to immune exclusion.

14. While not strictly mandatory for the current scope, it would be interesting to address whether tumor ECM exposure also remodels the glycosylation of the immune cells, thereby shaping function.

15. Regarding the adopted models scope envisaging translational relevance. The central findings rely on an elegant ex vivo platform using decellularized human tumor ECM recellularized with TNBC cell lines and CAR-T surrogates. I recognize the considerable challenges of providing in vivo validation in immunocompetent models. Nevertheless, the translational value of the manuscript would be substantially strengthened if key results were reproduced in well-characterized human tumor/ECM slices with autologous TILs under the glycosidases treatment.

Reviewer #3

(Remarks to the Author)
In the present manuscript Tyler et al. investigate the contribution of the glycosylated ECM on T cell infiltration and macrophage phenotype. They present a full characterization of the ECM glycosylation patterns, showing that tumor ECM carries more sialylated structures, resembling the desert phenotype TNBC tumors. This is an elegant study, adding yet another layer to the intricate role glycosylation plays in the tumor microenvironment. I do have a few issues listed below that the researcher should address to further strengthen their results.

Major comments:
- In figure 6 the authors only use the BT20 cell line to assess T cell infiltration and the induction of a desert phenotype. These results need to be confirmed using another cell line. Are similar results obtained in the same model but now using the HCC38 cells?

- For the T cell infiltration experiments the authors fully focus on the sialic acid-Siglec axis, completely ignoring other sialic acid-dependent receptors and adhesion molecules, most importantly the selectins. What is the expression of selectins on the CAR-T cells and are the selectins involved in the phenotype?

- I understand why the researchers omit the tumor cells and focus only on the macrophages in the experiments depicted in Figs 7 & 8. However, in patients the tumor cells are always present, influencing the macrophages through direct interactions or soluble mediators. Are the effects on macrophage phenotype and subsequent T cells activation still observed when tumor supernatants are mixed in in the co-culture.

- I am not convinced that just by having less CD11b+ macrophages you can draw conclusions about the suppressive nature of these cells. I therefore find the conclusion presented in lines 287-290 a substantial overstatement and I recommend to rephrase these lines.

- Along the same line, the increase in CD163+ macrophages on the neuraminidase treated ECM is explained as beneficial (lines 299-300). Without tumor cells (see above) one cannot conclude this. Actually, in most tumor types CD163 is considered a TAM marker associated with immune tolerance. In the Puttock paper by the same authors (Nat Comm 2023) they perform similar experiments with ECM from high and low disease patients. In this study CD163 is associated with high disease and a more suppressive T cell phenotype. How do the authors explain their results also in the context of other tumors and their previous paper?

- In fig. 7b the authors conclude that similar effects are not observed in adjacent ECM, however the same trend is clearly visible. It seems to me a numbers issue. Can the authors include 3 more donors and still draw that same conclusion?

- Supplementary figure 9 is not mentioned in the main text (maybe Suppl. Fig 10 reference in line 194, should be the suppl fig 9?).

Minor comments:
- Why did the authors co-culture the CAR-T cells first for 1 hour on the ECM before staining the Siglecs? One hour is not enough time for new proteins to come to the cell surface, so this seems like a redundant step to me. Did the authors observe differences in staining the CAR-T directly or after this 1 hour on the ECM?

- I would opt to move suppl figures 11C&D to the main figures, as these contain a crucial validation of the ECM components.

- Supplementary methods, line 277: PNA does not stain terminal β-galactose as stated, PNA is really highly specific for the Galβ1-3GalNAc linkage found in O-glycans. This needs correcting.

- Suppl. Fig 6b: PNA recognizes Gal-GalNAc, not Gal-GlcNAc

- Also some of the lectin images in the suppl. Figures need correcting:
Suppl. Fig 6A/11A: ConA does not interact with terminal mannoses, but actually binds the internal mannose core (also present in complex N-glycans).
Suppl. Fig 6A: SNA and MAL-I cannot bind the same sialic acid on an N-glycan, as this sialic acid is either α2-3 or α2-6 linked.
Both of these are confusing (and incorrect) and should be changed.

- The colors of figures 1g and 2e are difficult to distinguish, so I would suggest to alter the colors to make these figures more readable.

Version 1:

Reviewer comments:

Reviewer #1

(Remarks to the Author)
I feel the revised manuscript is substantially improved, more robust and focused. All my comments have been addressed and I support its publication.

Reviewer #2

(Remarks to the Author)
The revised manuscript by Tyler, Liu and Tarantola et al. presents a comprehensive and well-executed study addressing the role of ECM glycosylation in shaping the immune microenvironment of TNBC. By combining multi-omics profiling with

functional decellularized tissue models, the authors provide compelling evidence that ECM glycan remodeling, particularly increased sialylation, contributes to T cell exclusion, macrophage programming, and macrophage-T cell crosstalk.

The revised version of the manuscript represents a clear improvement over the original submission. The authors have reasonably addressed my concerns and strengthened the study through the inclusion of additional data, improved clarity in data presentation, and a more focused narrative. Overall, I consider the work to be novel, methodologically robust, and of clear relevance to the fields of tumor immunology and glycobiology, with potential translational implications.

That said, a few minor points should be addressed to further strengthen the mechanistic interpretation and overall clarity of the study:

1. Functional data convincingly show that enzymatic removal of glycans enhances T cell infiltration and alters macrophage phenotypes. However, the extent to which these effects are driven by glycan-mediated signaling versus changes in ECM architecture remains to be fully clarified. As glycosidase treatment is likely to impact matrix organization and physical properties, the resulting structural alterations may substantially contribute to the observed phenotypes. Given that this issue is central to the mechanistic interpretation of the study, it would be important to more explicitly acknowledge and discuss this limitation.

2. The classification of tumors into inflamed, excluded, and desert phenotypes provides a valuable framework. However, its integration with ECM glycosylation features and matrix-related signatures across the study remains somewhat limited. I appreciate that the authors have reasonably highlighted constraints related to model systems and intratumoral heterogeneity. Still, a clearer discussion of these limitations and their implications for translational interpretation would further improve the overall coherence of the manuscript.

In summary, this is a strong and timely study that provides important insight into how ECM glycosylation contributes to immune regulation in TNBC. The remaining points relate primarily to interpretation and framing.

Reviewer #3

(Remarks to the Author)
I thank the authors for their elaborative effort and the additonal experiments performed according to my suggestions. In my opinion this has significantly improved the manuscript.

Open Access This Peer Review File is licensed under a Creative Commons Attribution 4.0 International License, which permits use, sharing, adaptation, distribution and reproduction in any medium or format, as long as you give appropriate credit to the original author(s) and the source, provide a link to the Creative Commons license, and indicate if changes were made.
In cases where reviewers are anonymous, credit should be given to 'Anonymous Referee' and the source.
The images or other third party material in this Peer Review File are included in the article’s Creative Commons license, unless indicated otherwise in a credit line to the material. If material is not included in the article’s Creative Commons license and your intended use is not permitted by statutory regulation or exceeds the permitted use, you will need to obtain

permission directly from the copyright holder.
To view a copy of this license, visit https://creativecommons.org/licenses/by/4.0/

General Response to all reviewers

Thank you for your time and critical review of the manuscript. We have taken onboard your comments and now present a significantly revised manuscript. In particular we would like to highlight the removal of Figure 5 in the first version of the manuscript which presented our findings on ECM altering tumour cell phenotype. On reflection we felt that this figure was separate from the main messages of the paper and instead have added new data that further supports the key findings, as well as providing mechanistic insight. With these new data present the work provides further insight into the role of ECM regulating T-cell immunity, Macrophage immunity, and T-cell – macrophage crosstalk. Below is a point-by-point response to your questions/comments.

REVIEWER COMMENTS

Reviewer #1 (Remarks to the Author):

In this manuscript, the authors described a novel role for extracellular matrix (ECM) glycosylation in promoting immune suppression in triple negative breast cancer (TNBC).
The authors provided a detailed characterisation of decellularised ECM from TNBC tumours and adjacent tissues, at the mRNA, protein and glycosylation levels. This indicated significant changes both at the protein levels, as well as changing the the glycosilation of ECM components. In addition, they showed that tumour ECM prevented T cell infiltration, was linked to epithelial to mesenchymal transition (EMT) in tumour cells and affected macrophage differentiation.
Enzymatic removal of glycosylation promoted anti-tumour immunity, suggesting this might represent a novel therapeutic strategy.

This study is rigorous and well written, however some aspects appear quite preliminary and further work is need to strengthen the conclusions and provide more mechanistic information.

1. The decellularisation protocol is described in detail. The authors show in figure 4d that this results in a significant increase in adjacent ECM stiffness (but not tumour). Why is this? Is this affecting the cellular responses to this matrix?

Response. We have added additional samples to include more data points for this graph (Updated Fig.1). By doing this we noticed that changes in stiffness between cellularized and decellularized adjacent tissue is not significant. There is still a trend toward increased stiffness, but this might be explained as adjacent tissue contains lots of adipocytes and by removing fatty areas can induce a small increase in stiffness. These data are referred to in page 5 (line 197-198) and the new graph is shown in Fig.4d.

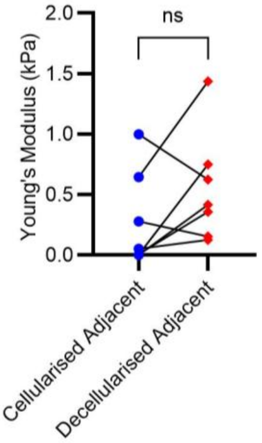

Updated Fig.1 - Tissue stiffness of cellularized and decellularized tumor and adjacent tissues using Instron 3342 with a 10N load cell. Paired t test. N=5 each

2. The authors perfomed live/dead staining of cancer cells seeded on collagen, tumour or adjacent decellularised ECM in both figure 4f,g and figure 5a,b. The confluency of the cells appears very different at day 1 across the conditions tested in figure 4 (and the quantification is only N=2). HCC38 seemed to be progressively lost from all the ECM conditions in figure 4, but not in figure 5, while there seem to be a lot more dead cells in figure 5 compared to figure 4. Can the authors clarify these differences or provide additional repeats to strengthen these observations?

Response. Cell confluency may differ between the conditions because cells seeded within collagen gels remain embedded in the matrix, whereas cells seeded directly onto the tissue can sometimes migrate away into the well. Our interpretation of this graph is that cell viability is not affected when cells are cultured on tissues compared with collagen gels. To improve robustness we have repeated the experiment (N = 3,) which showed the same results : viability is unchanged on tissues compared to collagen gels (Updated Fig.2). These data are referred to in page 5 (line 204-205) and the new graph is shown in Fig.4g.

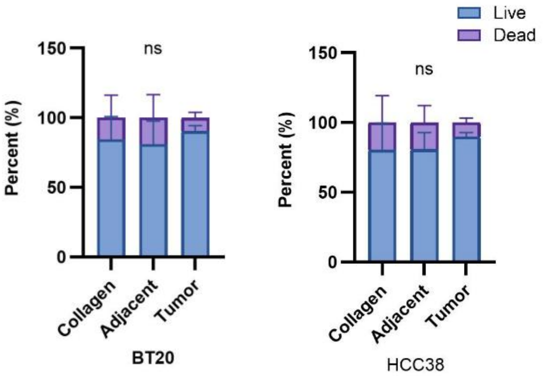

Updated Fig.2 - Image analysis of Day 7 BT20 and HCC38 cell viability between cells seeded on collagen-coated plastic, adjacent tissue, and tumor tissue. Data are presented as mean

3. The EMT data feel very preliminary (some are N=1). There appears to be cell-line dependent changes in gene expression. Some changes highlighted in the text (such as the increase in collagen I expression in cancer cells), are not statistically significant when comparing tumour versus adjacent ECM. The phenotype observed seems more partial EMT. I think the authors should characterise in more detail the expression of different EMT markers, including both epithelial and mesenchymal ones, both at the mRNA and protein levels. Once a more detailed characterisation is provided, the effect of glycosylation removal should be tested in both cell lines.

Response. In the revised manuscript we have removed this figure because on reflection it does not fit with the core message of the manuscript. This figure provided information on how ECM glycosylation associated with tumour cell phenotype, however the core focus of the paper is on how ECM glycosylation regulates immune cell phenotype. Our hope is that this revision focuses the paper and along with the new data added provides a more insightful presentation of ECM regulated macrophage and T-cell immunity.

4. When assessing T cell infiltration in vitro (figure 6), why only BT-20 cells were used? The quantifications in c-f are a bit difficult to follow. Why were the data not combined in a single graph showing cells near and far from tumours, in both tumour and adjacent ECM?
In the representative images shown, it seems that treatment with PNGase F resulted in much bigger tumour islets, compared to the other conditions. Since the authors showed that changes in the T cell movement/infiltration required the presence of cancer cells, here it is crucial to determine how glycosylation removal affects the formation of cancer cell islets.

Could changes in T cells infiltration be linked to EMT in cancer cells? EMT has been shown to reduce T cell infiltration in lung cancer (PMID: 29440769), therefore it'd be interesting to investigate whether the same is happening here.

Response. We agree that it would be interesting to see what the effect on T cells is when co-cultured on top of PNGase F and Neuraminidase treated tissue in presence of a different cancer cell line. For this reason, we followed the reviewer suggestion and repeated the whole experiment with HCC38, another TNBC cell line, which is a bit more mesenchymal-like, compared to BT20. Additionally, we decided for this experiment to use PBMCs-isolated T cells, and subsequently activated, to better recapitulate the human setting.

Similarly to the BT20 experiment already performed for the original version of the manuscript (now moved to Supplementary Fig.13), we demonstrated that when comparing T cell infiltration between tumor-derived ECM and adjacent tissue, T cell infiltration was significantly reduced in tumor-derived ECM (Updated Fig. 3a). Furthermore, fewer T cells penetrated the tumor nest (≤5 μm from a cancer cell) in tumor ECM compared to adjacent tissue (Updated Fig. 3b), resembling an immune-excluded phenotype. In both tumor and adjacent tissues, T cells preferentially accumulated in the stroma (Fig.6b). We have also combined the graphs showing T cell number near and far from tumor in adjacent and tumor tissue in one graph (Updated Fig.3b).

Analysis of T cell motility revealed that T cells located near a cancer cell (<5μm) have an increased speed and displacement in adjacent tissues compared to tumor tissues (Updated Fig. 3c,d). Moreover, within tumor ECM, T cells that are located near tumor islets exhibited a decreased speed and displacement compared to those in the surrounding ECM (Updated Fig. 3e,f).

We then wanted to test whether enzymatic removal of N-glycans or sialic acids significantly enhanced T cell infiltration into tumor ECM (Updated Fig. 3g) and increased the proportion of T cells penetrating the tumor nest (Updated Fig. 3g,h). Interestingly, T cells in PNGase F or neuraminidase-treated tissues, compared to untreated tumor tissue, exhibited greater and improved speed and displacement when in proximity of a cancer cell (<5µm), indicating that T cells are more easily able to move and reach the tumor islets through the ECM in enzyme-treated tissues (Updated Fig. 3j,k).
These data are referred to in page 5-7 (line 206-253) and the new graph is shown in Fig.5.

Although we have removed the EMT figure, our preliminary data indicated that EMT-related gene expression and matrix gene expression were significantly reduced in HCC38 cells cultured on decellularized tumor tissue pre-treated with PNGase F and neuraminidase compared to untreated tumor ECM. In the new data presented we find evidence that a selectin mediated mechanism may explain the improved T-cell infiltatration with glycosidase treated tissues.

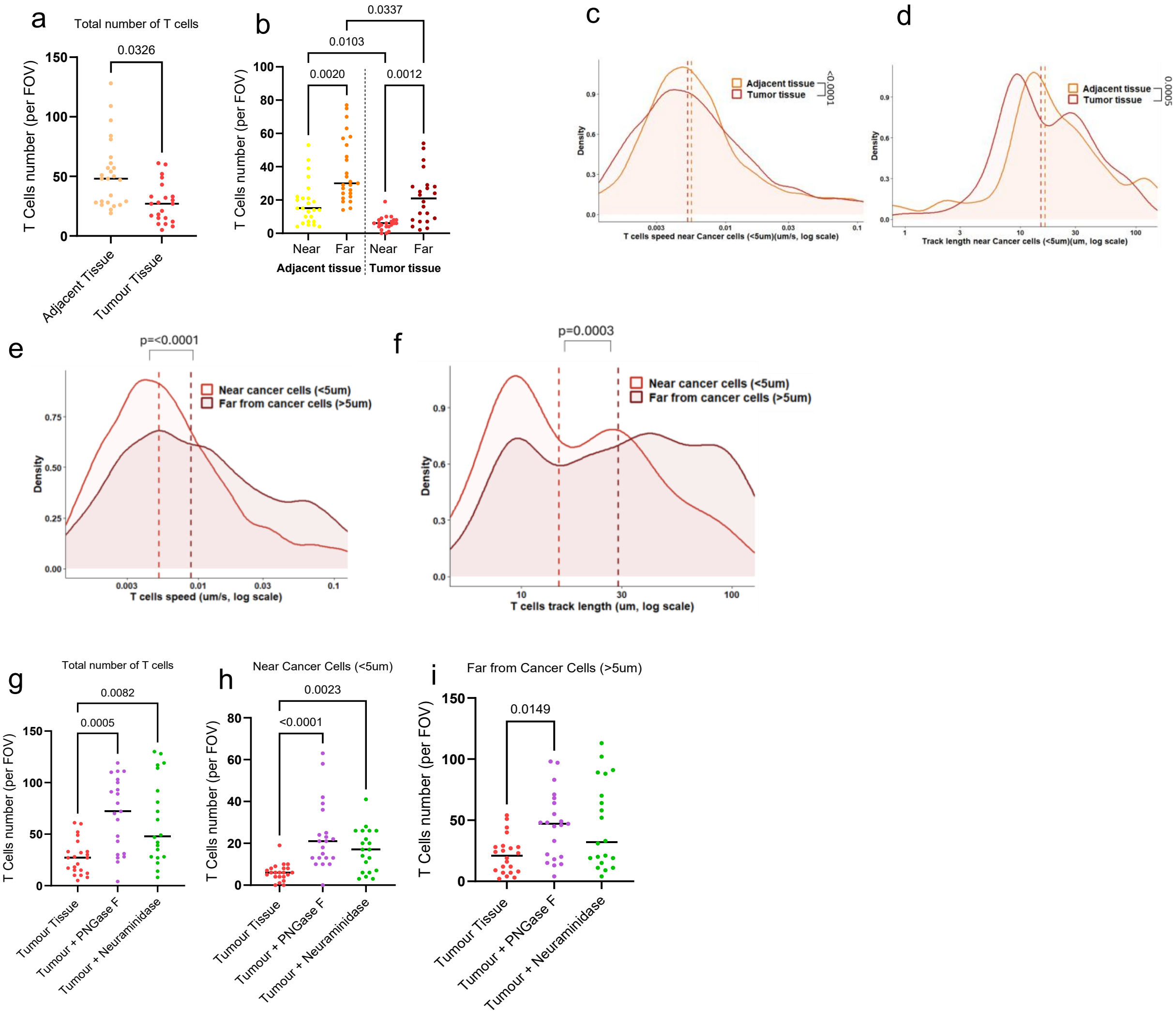

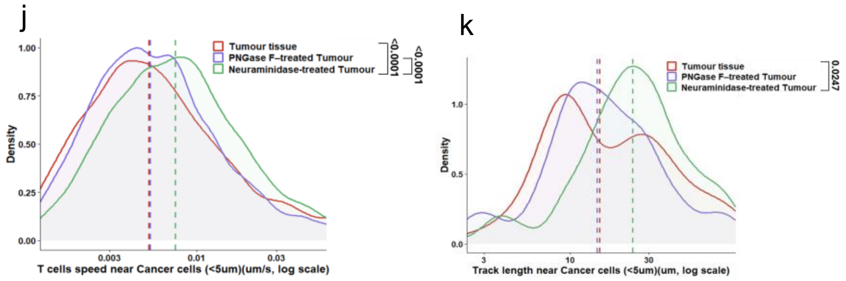

Updated Fig.3 – a-b, Count of **a,** total T cell number in each field of view (FOV) and **b,** T cell number near (<5μm) and far (>5μm) from a cancer cell in tumor and adjacent tissues recellularized with HCC38 cells. N=3 tissue donors for each group, 5-10 FOV per tissue. Unpaired T-test. **c,** Density distributions of T cell speed near (<5 μm) cancer cells on tumor tissue (red) and adjacent tissue (orange). **d,** Density distributions of T cell track length near (<5 μm) cancer cells on tumor tissue (red) and adjacent tissue (orange). **c-d,** Dashed lines indicate medians. Data presented in log scale. Kruskal-Wallis test with Dunn's multiple comparisons test. N=3, with 2 technical replicates each. **e,** T cell speed (μm/s) near (<5μm) and far (>5μm) from cancer cells in tumor tissue. **f,** T cell track length (μm) near (<5μm) and far (>5μm) from cancer cells in tumor tissue. **e-f,** Line at median. Data presented in log scale. Mann-Whitney test. N=3, with 2 technical replicates each. **g-i,** Count of **g,** total T cell number in each FOV, **h,** T cell number within 5μm of a tumor cell ('near cancer cells') and **i,** T cell number more than 5μm from a cancer cell ('far from cancer cells'). N=3 tissue donors, 6-10 FOV per tissue. One-way ANOVA with Dunnett's multiple comparison's test. **g-i,** Line at median. **j,** Density distributions of T cell speed near (<5μm) cancer cells on tumor tissue (red), tumour + PNGase F treated tissue (violet) or tumour + neuraminidase treated tissue (green). **k,** Density distributions of T cell track length near (<5μm) cancer cells on tumor tissue (red), tumor + PNGase F treated tissue (violet) or tumor + neuraminidase treated tissue (green). **J-k,** Dashed lines indicate medians. Data presented in log scale. Kruskal-Wallis test with Dunn's multiple comparisons test. N=3, with 2 technical replicates each.values +/- standard deviation (SD). Two-way ANOVA with Sidak's multiple comparisons test. N=3.

5. The live cell imaging results are not very convincing, as the graphs show minimal differences in speed (despite the extremely high statistical significance)

Response. We understand the concern regarding these data. We have plotted the speed and track length of each individual T cell in a different plot, hopefully showing more convincingly that there is an shift in T cell speed and displacement. We have also confirmed our initial observation with a second cell line. Please, refer to the previous question, Updated Fig. 3c-f and Updated Fig 3j,k.

6. Removal of glycosylation resulted in profound changes to the overall ECM architecture, as expected as glycosylation is required for the maintenance of ECM networks. It will be important to determine whether the changes observed here are due to physical alterations of the ECM, or changes in cell/ECM interactions, which are also modulated by glycosylation levels.

Response. Removal of glycosylation was performed only on tissues and not on cancer cells. The decellularised tissue is treated with a glycosidase prior to seeding with cells. For this

reason, the effect that we show is due solely to changes of the level of glycosylation of the ECM and subsequential change in ECM structure, that then might have an impact on cells as well. Specifically, whether the changes of ECM glycosylation have an impact on cancer cells will be better investigated in future work. In the revised manuscript we are focusing on how ECM-glycosylation changes effect T cells and macrophages.

7. Macrophage differentiation: only CD45+ cell frequency is reported to be significantly different between tumour and adjacent ECM, while all the other markers were not changed. I think if is essential to better define the differences between tumour and adjacent ECM, before determining the role of glycosylation. In the data provide in the manuscript, the removal of glycosylation mostly has the same effect on tumour and adjacent ECM - why is this? Could this be driven by changes in overall ECM architecture, rather than being specific for ECM glycosylation in tumours?

Response. From our analysis we found a positive correlation between increased mannose and N-linked glycans in tumour tissues with T cells, dendritic cells and B cell signatures, suggesting a relationship between ECM glycome and adaptive immune infiltrates (line 171-175). In contrast, we did not find strong evidence for differences in macrophage abundance.

The main change between adjacent and tumor tissue was the downregulation of CD11b expression on tumor tissue (Updated Fig.4). Given that CD11b has been implicated in regulating myeloid cell polarization and functional states, this shift suggests that tumor ECM exposure may be associated with altered macrophage differentiation or immunomodulatory programs.

These data are referred to in page 7 (line 295-299) and the new graph is shown in Supplementary Fig.22a.

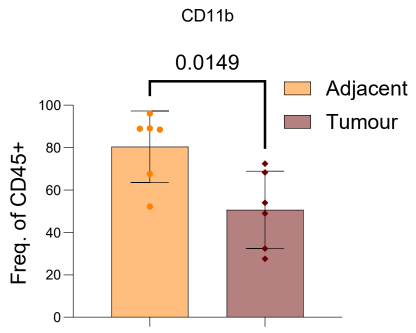

Supplementary Fig.4 Barchart of flow cytometry expression patterns of CD11b shown as percentage of positive cells from the CD45+ population. Line at mean with standard deviation error bars. Mixed-effects analysis with Tukey's multiple comparisons test. N=6 (N=6 adjacent, N=6 tumor, N=6 blood cones).

Although glycosylation is also present, albeit at lower levels, in adjacent tissues, these adjacent tissues may already be undergoing structural and molecular changes due to their proximity to the tumour. For this reason, we included an additional control using healthy tissues obtained from cancer-free donors.
The goal of this experiment was to determine whether removal of sialic acid alters macrophage phenotype. We treated tumor ECM with neuraminidase which significantly increased the proportion of CD11b+, CD163+ and CD209+ macrophages compared to untreated tumor tissues. This effect was not observed on neuraminidase-treated adjacent or healthy ECM,

implicating only sialic acids displayed on tumor ECM as modulators of macrophage phenotype (Updated Fig.5a,d). Combinatorial flow analysis of immunomodulatory markers (CD163, CD206, CD209) revealed that the proportion of macrophages co-expressing two activation markers was significantly increased on neuraminidase-treated tumor tissue (Fig. 6c-d), suggesting that ECM-sialic acids play a role in immunomodulatory changes of macrophage phenotype in the TME (Updated Fig.5e-g). These data are referred to in page 7-8 (line 284-316) and part of these new graphs are shown in Fig.6.

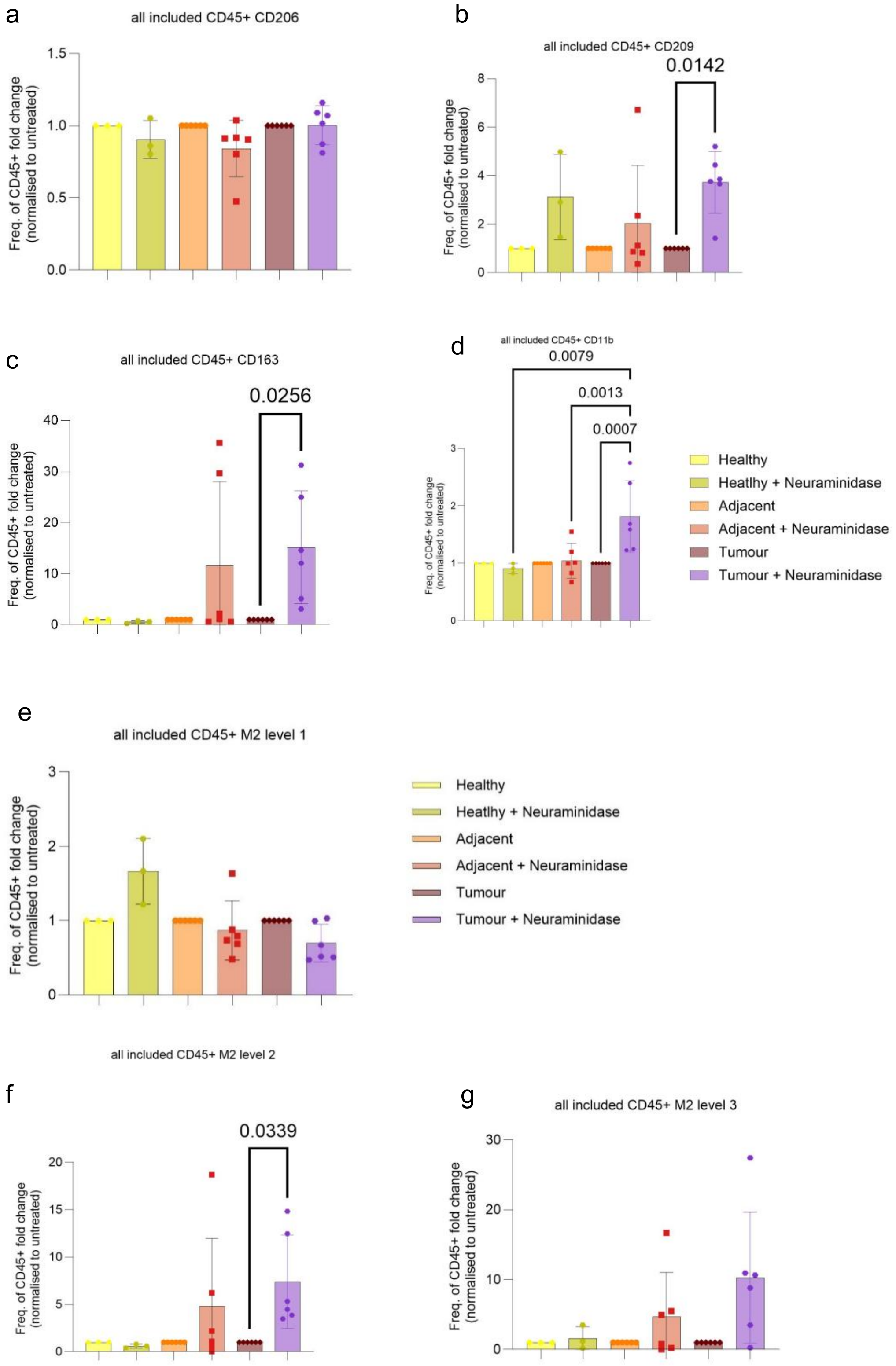
a
all included CD45+ CD206
Freq. of CD45+ fold change (normalised to untreated)
1.5
1.0
0.5
0.0
b
all included CD45+ CD209
0.0142
Freq. of CD45+ fold change (normalised to untreated)
8
6
4
2
0
c
all included CD45+ CD163
0.0256
Freq. of CD45+ fold change (normalised to untreated)
40
30
20
10
0
d
all included CD45+ CD11b
0.0079
0.0013
0.0007
Freq. of CD45+ fold change (normalised to untreated)
3
2
1
0
Healthy
Heatlhy + Neuraminidase
Adjacent
Adjacent + Neuraminidase
Tumour
Tumour + Neuraminidase
e
all included CD45+ M2 level 1
Freq. of CD45+ fold change (normalised to untreated)
3
2
1
0
Healthy
Heatlhy + Neuraminidase
Adjacent
Adjacent + Neuraminidase
Tumour
Tumour + Neuraminidase
all included CD45+ M2 level 2
f
0.0339
Freq. of CD45+ fold change (normalised to untreated)
20
15
10
5
0
g
all included CD45+ M2 level 3
Freq. of CD45+ fold change (normalised to untreated)
30
20
10
0

Supplementary Fig.5 a-d, Barchart of flow cytometry expression patterns of CD11b, CD163, CD206, CD209, shown as fold change normalized to untreated tissue of positive cells from the CD45+ population. Line at mean with standard deviation error bars. Mixed-effects analysis with Tukey's multiple comparisons test. N=6 (N=3, healthy, N=6 adjacent, N=6 tumor, N=6 blood cones). **e-g**, Barchart of combinatorial gating for activation markers (CD163, CD206, & CD209) shown as fold change normalized to untreated tissue of positive cells expressing one marker (activation level 1), two markers (activation level 2), or three markers (activation level 3) from the CD45+ population. Line at mean with standard deviation error bars. Mixed-effects analysis with Tukey's multiple comparisons test. N=6 (N=6 adjacent, N=6 tumor, N=3 blood cones).

Reviewer #2 (Remarks to the Author):

The manuscript by Tyler, Liu and Tarantola et al., entitled “Glycosylated extracellular matrix drives immune suppression by controlling T-cell movement, macrophage phenotype, and macrophage–T-cell crosstalk in triple-negative breast cancer,” provides relevant and novel findings linking alterations in ECM glycosylation, particularly N-glycan sialylation, to immune exclusion. This aligns with and expands beyond previous work on ECM/matrisome signatures and proteoglycans, further supporting that changes in ECM components critically influence T-cell trafficking and macrophage programming. Building on previous findings, the authors started by exploiting a TCGA matrix-index analysis to demonstrate links between alterations in ECM components linked to glycosylation and immune exclusion. They further explore their own patient samples employing bulk RNA-seq, ECM-enriched proteomics, MALDI-TOF glycomics, lectin histology, and human decellularized-tissue models re-cellularized with TNBC cells. They show that tumor ECM compared with matched adjacent/surround tissue display increased sialylation, fucosylation and poly-LacNAc on N-glycans. Among these features, elevated sialylation appears to better stratify immune-excluded tumors. Enzymatic removal of N-glycans and/or terminal sialic acids increased $CD8^+$ CAR-T infiltration and motility, reduced EMT-associated programs in tumor cells, and reprogramed macrophages toward a more activated phenotype. This reprogramming has been shown to modulate macrophage–T-cell crosstalk. This behavior was not observed for adjacent/surround ECM, supporting a cancer-dependent effect. While mechanistic aspect are not fully resolved and in vivo validation remains to be accomplished, the data supports that targeting and rewiring ECM glycosylation may provide a path to convert immune-excluded TNBC into a more inflamed state. Overall, the methodological approach is appropriate and generally adequate to support these conclusions. The manuscript is well organized and easy to follow. The observations are novel and meaningful to better understand the intricate crosstalk between the ECM and the immune system, with implications for cancer progression.

The findings are relevant and may hold translational implications. However, some concerns persist, as follows:

1. The TCGA analysis is based on all invasive breast cancers. On the other hand, experimental work is focused on TNBC. To strengthen clinical relevance, the MI–survival and MI–pathway enrichment could be addressed, if possible, specifically within TNBC cases. This would better align with the mechanistic focus of the study.

Response. We have now run the analysis of MI-survival and MI-pathway enrichment within TNBC cases. The analysis shows the same result where high MI correlates with poor prognosis. Also in this case, high MI tissues were enriched for genes associated with N-linked glycosylation and suppression of adaptive immunity (Updated Fig.6), supporting the hypothesis that glycans displayed on ECM proteins confer immunoregulatory properties of TNBC ECM. These data are referred to in page 3 (line 116-121) and these new graphs are shown in Supplementary Fig.1a-b.

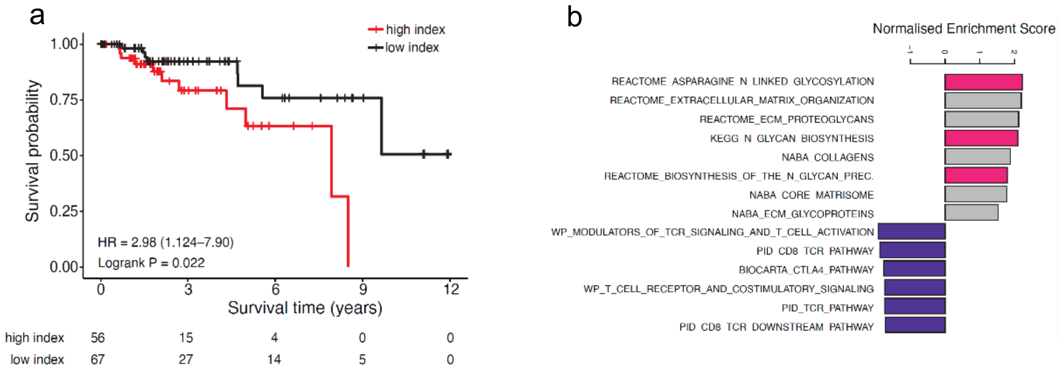

Updated Fig.6 - a, Kaplan-Meier survival curve for matrix index (MI) high (56 patients) vs MI low (67 patients) from TCGA invasive breast cancer dataset. **b,** Differentially expressed genes between MI high and MI low patient samples are enriched for genes upregulated in ECM remodelling (grey bars) and post-translational glycosylation (pink bars) and genes downregulated in immunity (blue bars).

2. When addressing patient samples, the manuscript introduces an inflamed/excluded/desert stratification of tumors based on T-cell infiltration. However, this classification was not fully and consistently integrated into the subsequent analyses. The narrative would be significantly strengthened if the authors could explicitly connect these immune classes to the ECM glycosylation features and to the MI framework introduced in the beginning of the work across the manuscript. Linking these features to functional implications would significantly broaden the translational character of the findings, providing grounds for future decision-making.

Response. We agree this would be very nice, and we have explored this possibility. However, we have concluded that the way we currently use the decellularized tissue model makes assigning a phenotype challenging. We think this is because of our previous findings (Hirani et al.), where immune phenotype analysis revealed substantial intra-tissue heterogeneity, with 69% of tissue sections exhibiting two or more immune phenotypes. This heterogeneity was observed across all three immune phenotypes; notably, all excluded tissues contained multiple phenotypes. Because our decellularized tissue model samples only a small disc from the original tissue, it is inherently challenging to capture a representative profile of the entire tumor. Consequently, localized immune heterogeneity may not be fully reflected in our analyses. We hope to overcome this limitation through further development of the model in the future.

3. Glycomics data was obtained for ECM but also membrane fractions and whole cells. However, it is not always clear how well these fractions are separated and the level of

contamination between compartments, which may influence the observed profiles. Therefore, the authors should clarify the fractionation workflow, purity checks, and control strategy.

Response. Before isolation and purification of glycans from membrane and ECM proteins, we firstly isolated different compartmental proteins from patients tissue: Cytoplasmic, Nuclear, Membrane, Cytoskeleton and ECM Proteins, using CNMCS Compartmental Protein Extraction Kit (Cat no: C6012-25, US Biological) previously described (PMID: 26273955; PMID: 22159717) and also shown in Supplementary Fig. 1, which can enrich ECM proteins from tissues, digest into peptides for Mass Spectrometry Analysis (PMID: 22159717; PMID: 29196464), and avoid glycans mixtures among different protein fractions. This part of the protocol is now added in the supplementary methods.

4. The glycan data is reported as relative percentages of permethylated glycans identified by MALDI-TOF. Because percentages are interdependent (if one glycan goes up, others go down), ANOVA analysis may not be adequate for data interpretation and capturing real differences. Also, data normalization of complementary quantitative methods, for instance LC-MS/MS with standards, would provide more unequivocal information.

Response. We appreciate the reviewer's comment and agree in principle that orthogonal quantitative approaches, such as LC-MS/MS with appropriate standards, could provide additional quantitative confidence. However, the scope and practical constraints of this study limit the feasibility of such analyses. This work is an ambitious, multi-modal investigation that integrates several experimental techniques and relies on rare, clinically derived patient tissues that are extremely difficult to obtain. As a result, both sample number and available material were highly limited, necessitating the use of a single MS-based glycomic approach.

While we acknowledge that relative abundance data are compositional in nature, reporting permethylated glycan profiles by MALDI-TOF MS remains a well-established and widely accepted strategy in glycomics for comparative analyses across biological conditions. Moreover, although absolute quantification using glycan standards would indeed be desirable, the availability of comprehensive quantitative standards for the wide diversity of glycans analyzed here is currently very limited. Unlike proteomics (where isotope-labeled peptide standards are relatively straightforward to obtain) glycan synthesis and standardization remain a significant technical challenge in the field.

We therefore believe that the analytical approach used represents a pragmatic and appropriate balance between technical rigor and feasibility, given the complexity of the samples and the exploratory nature of the study.

5. Glycomics datasets are imbalanced, with large tumor cohorts contrasting with few adjacent controls. The limited representation of adjacent tissue diminishes the robustness of subsequent comparisons. Furthermore, it raises some concerns that the reported differences could be driven by individual variability. Expanding the number of adjacent ECM samples would significantly strengthen the conclusions.

Response. We focused our analyses on matched tumor and adjacent tissues, which helps to minimize inter-patient variability. Our objective was to investigate the relationship between extracellular matrix composition, glycosylation patterns, and immune infiltration; including a larger number of adjacent tissues would therefore not be informative, as these regions typically exhibit minimal immune infiltration. Moreover, as noted above, the availability of patient-derived

tissues represents a significant practical limitation, further constraining sample numbers.

6. Following MS-assisted glycomics, the manuscript presents a broad lectin survey of ECM glycosylation (MAL-II, VVA, UEA-I, and others) that supports glycome remodeling. However, in the functional assays the focus narrows almost exclusively to SNA (α2,6-sialylation) and ConA (mannose-binding). Other apparently relevant lectins are not revisited. There also seems to be a shift from MAL-II (α2,3-sialylation) in the initial survey to SNA (α2,6-sialylation) in functional studies. This raises doubts about the actual sialylation changes which are most relevant. It would be important to explain why α2,6-sialylation was prioritized, especially after initially emphasizing α2,3 sialylation. Furthermore, it would be important to address if a broader lectin signals also change under functional conditions.

Response. We intentionally used a prolonged (24 h) incubation of neuraminidase at a high concentration (0.5 U) to facilitate cleavage across a broad spectrum of sialic acid linkages, thus dissecting the individual contributions of each linkage type was beyond the original scope of this study. We absolutely agree with a future expansion of the study to explore sialic acid linkage and more glycosidases and immune cell types as a follow-on study.

As suggested by the reviewer, we have used SNA lectin binding as a marker of α 2,6-linked sialylation and MAL I as a marker for α 2,3-linked sialylation. α 2,6-linked sialylation is significantly increased in tumour tissue compared to adjacent whereas α 2,3-linked sialylation is not significantly changed between adjacent and tumour. We have also stained following neuraminidase treatment on tumour tissue and found that α 2,6 levels were significantly decreased but α 2,3 were unchanged (Update Fig7), we plan to explore this in-depth in future work.

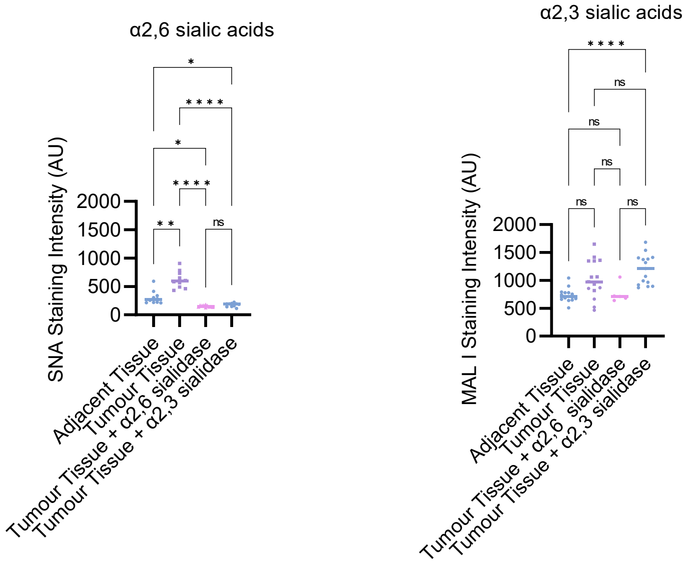

Updated Fig.7 - Analysis of SNA and MAL I lectin staining intensity using IMARIS image analysis software. N=2 matched tissue donors for each group, 5 FOV per tissue, One-way ANOVA with Tukey's post-hoc test**.**

7. Decellularize ECM are repopulated with cancer cell models to address the functional impact

of the matrix at multiple levels. Please clarify whether the two used TNBC lines (BT20 and HCC38) differ in baseline glycosylation and/or ECM-remodeling profiles and if their differences are relevant for the observed outcomes. This may provide a more comprehensive picture on the observed crosstalk.

Response. The primary objective of this study was to determine the impact of sialic acid removal from the ECM and to assess how this modulates immune cell behavior and phenotype. We acknowledge that the inclusion of data examining changes in cancer cells may have diverted focus from the immune-centered aims of the manuscript and potentially caused confusion for the reader. For this reason, we have removed Figure 5 and the ECM data of TNBC cell lines,.

We agree that investigating the effects of sialic acid removal directly on cancer cells is an important and interesting question; however, this falls beyond the scope of the current study and will be addressed in future work.

8. To me it was rather difficult to identify which TNBC line was used for each assay. As it reads, most CAR-T infiltration and enzyme-rescue experiments appear to rely on BT20, whereas HCC38 is mainly presented for ECM phenotyping. If this is the case, it would be important to replicate key findings with both lines to support generalizability across TNBC models. It should also be made explicit that macrophage education experiments are performed directly on patient ECM without tumor cells, which carries distinct implications for interpreting cell–ECM crosstalk. Notably, performing these assays in the presence of cancer cells, even though not mandatory, would strength the current observations.

Response. We understand the confusion on that part of the manuscript. As mentioned before, we decided to remove the EMT data from the revised manuscript, which, as highlighted from the reviewer, was very preliminary and only conducted in one cell line, and therefore something we would like to develop further for a separate future manuscript. Regarding the data for T cell motility in the ECM, we agree that it would be interesting to see what the effect of T cells is when co-cultured on top of PNGase F and Neuraminidase treated tissue in presence of a different cancer cell line. For this reason, we followed the reviewer suggestion and repeated the whole experiment with HCC38, another TNBC cell line, which is a bit more mesenchymal-like, compared to BT20. Additionally, we decided for this experiment to use PBMCs-isolated T cells, and subsequently activated, to better recapitulate the human setting.

Similarly to the BT20 experiment already performed for the original version of the manuscript (now moved to Supplementary Fig.13) , we demonstrated that comparing T cells infiltration between tumor-derived ECM and adjacent tissue, T cells infiltration was significantly reduced in tumor-derived ECM (Updated Fig. 3a). Furthermore, fewer T cells penetrated the tumor nest (≤5 μm from a cancer cell) in tumor ECM compared to adjacent tissue (Updated Fig. 3b), resembling an immune-excluded phenotype. In both tumor and adjacent tissues, T cells preferentially accumulated in the stroma (Fig.6b). We have also considered the reviewer comment of combining the graphs showing T cell number near and far from tumor in adjacent and tumor tissue in one graph (Updated Fig.3b).

Analysis of T cell motility revealed that T cells located near a cancer cell (<5μm) have an increased speed and displacement in adjacent tissues compared to tumor tissues (Updated Fig. 3c,d). Moreover, within tumor ECM, T cells that are located near tumor islets exhibited a

decreased speed and displacement compared to those in the surrounding ECM (Updated Fig. 3e,f).

We then wanted to test whether enzymatic removal of N-glycans or sialic acids significantly enhanced T cell infiltration into tumor ECM (Updated Fig. 3g) and increased the proportion of T cells penetrating the tumor nest (Updated Fig. 3g,h). Interestingly, T cells in PNGase F or neuraminidase-treated tissues, compared to untreated tumor tissue, exhibited greater and improved speed and displacement when in proximity of a cancer cell (<5μm), indicating that T cells are more easily able to move and reach the tumor islets through the ECM in enzyme-treated tissues (Updated Fig. 3j,k).
These data are referred to in page 5-7 (line 206-253) and the new graph is shown in Fig.5.

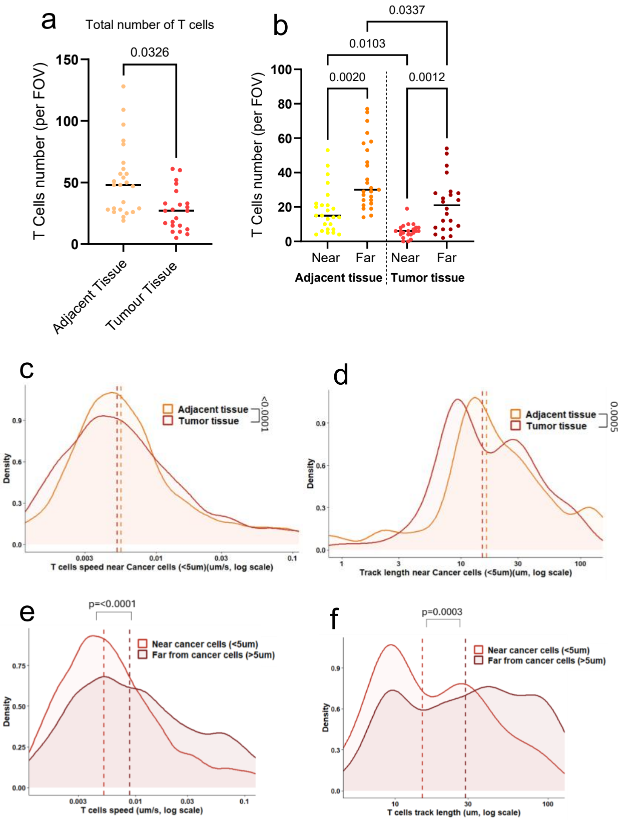

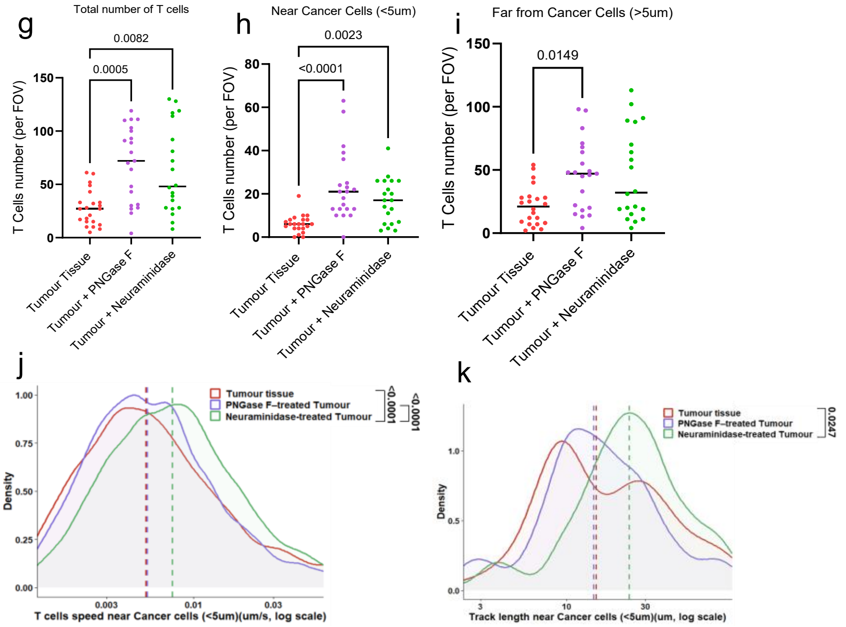

Updated Fig.3 – a-b, Count of **a,** total T cell number in each field of view (FOV) and **b,** T cell number near (<5µm) and far (>5µm) from a cancer cell in tumor and adjacent tissues recellularized with HCC38 cells. N=3 tissue donors for each group, 5-10 FOV per tissue. Unpaired T-test. **c,** Density distributions of T cell speed near (<5 µm) cancer cells on tumor tissue (red) and adjacent tissue (orange). **d,** Density distributions of T cell track length near (<5 µm) cancer cells on tumor tissue (red) and adjacent tissue (orange). **c-d,** Dashed lines indicate medians. Data presented in log scale. Kruskal-Wallis test with Dunn's multiple comparisons test. N=3, with 2 technical replicates each. **e,** T cell speed (µm/s) near (<5µm) and far (>5µm) from cancer cells in tumor tissue. **f,** T cell track length (µm) near (<5µm) and far (>5µm) from cancer cells in tumor tissue. **e-f,** Line at median. Data presented in log scale. Mann-Whitney test. N=3, with 2 technical replicates each. **g-i,** Count of **g,** total T cell number in each FOV, **h,** T cell number within 5µm of a tumor cell ('near cancer cells') and **i,** T cell number more than 5µm from a cancer cell ('far from cancer cells'). N=3 tissue donors, 6-10 FOV per tissue. One-way ANOVA with Dunnett's multiple comparison's test. **g-i,** Line at median. **j,** Density distributions of T cell speed near (<5µm) cancer cells on tumor tissue (red), tumour + PNGase F treated tissue (violet) or tumour + neuraminidase treated tissue (green). **k,** Density distributions of T cell track length near (<5µm) cancer cells on tumor tissue (red), tumor + PNGase F treated tissue (violet) or tumor + neuraminidase treated tissue (green). **J-k,** Dashed lines indicate medians. Data presented in log scale. Kruskal-Wallis test with Dunn's multiple comparisons test. N=3, with 2 technical replicates each.values +/- standard deviation (SD). Two-way ANOVA with Sidak's multiple comparisons test. N=3.

9. The manuscript introduces MDA-MB-468 cells for the 2D CAR-T cytotoxicity and cytokine release assays without providing context. Please clarify the rationale for including this additional line, and why the cell line was not considered earlier.

Response. We understand the reviewer's concern regarding the introduction of an additional cancer cell line (MDA-MB-468). These experiments were initially performed as CAR-T validation studies to confirm targeting of TNBC cell lines. MDA-MB-468 exhibited behavior very similar to

HCC38, which is why subsequent experiments were conducted exclusively using HCC38. However, we acknowledge that inclusion of MDA-MB-468 may be confusing in the context of the manuscript. To improve clarity and focus, we have removed all mention of MDA-MB-468 from the revised version.

10. The authors show that Siglec-7 and Siglec-10 levels on T cells do not change after ECM desialylation. Based on this observation the authors argue against Siglec signaling. However, what actually changes in these assays is the availability of sialylated ligands in the ECM. The fact that receptor expression stays constant does not necessarily mean the receptors are not involved. In fact, if there are fewer ligands to engage with, the inhibitory signaling should go down, which fits quite well with the increased T-cell infiltration seen after neuraminidase treatment. Nevertheless, to really pin down the mechanism, the author could directly test ligand–receptor interactions and dependence. For example, checking Siglec-Fc binding to ECM before and after desialylation, blocking Siglecs with antibodies or sialic acid competitors, or knocking out siglecs in T cells and macrophages (which is rather hard to do) would provide a much clearer answer. Also, knowing exactly the nature of the sialic acids involved could provide a more educated notion about the siglecs or other immune ligands involved. This reinforces the need for more informative glycomics.

Response. We thank the reviewer for this thoughtful and constructive interpretation. We agree that unchanged Siglec-7 and Siglec-10 expression on T cells does not, by itself, exclude a role for Siglec-mediated signaling. As the reviewer correctly points out, ECM desialylation primarily alters ligand availability rather than receptor expression, and reduced engagement of inhibitory Siglecs would indeed be consistent with the increased T cell infiltration observed following neuraminidase treatment.

While we therefore cannot exclude a contribution of Siglec signaling, our current data do not provide sufficiently strong or direct evidence to justify a more extensive mechanistic dissection of this axis within the scope of the present study.

We fully agree that a more detailed characterization of the sialic acid structures present in the tumor ECM would help identify the relevant immune receptors, underscoring the value of advanced glycomic approaches for future work.

In parallel, and as suggested by the reviewer, we explored alternative sialic acid-dependent pathways and focused on selectins. We observed that CLA, CD62L (L-selectin), and CD162 (PSGL-1) are highly expressed on T cells in our model (Updated Fig. 8a,b). Notably, MAM-educated T cells within tumor tissue exhibited significantly increased CD62L and CD162 expression compared to adjacent tissue (Updated Fig. 8c). This phenotype is consistent with a less cytotoxic and less infiltrative T-cell state, as previously reported. Importantly, neuraminidase treatment of tumor tissues significantly reduced CD62L and CD162 expression, in line with enhanced cytotoxic potential, reduced exhaustion, and improved infiltration capacity of T cells in the tumor microenvironment.

Together, these data suggest that tumor ECM sialylation influences T cell phenotype and function, potentially through multiple sialic acid-dependent pathways, including selectin-mediated interactions, while Siglec involvement remains an important avenue for future mechanistic studies. These data are referred to in page 9 (line 367-379) and the new graph is shown in Fig.7f and Supplementary Fig 24d,e.

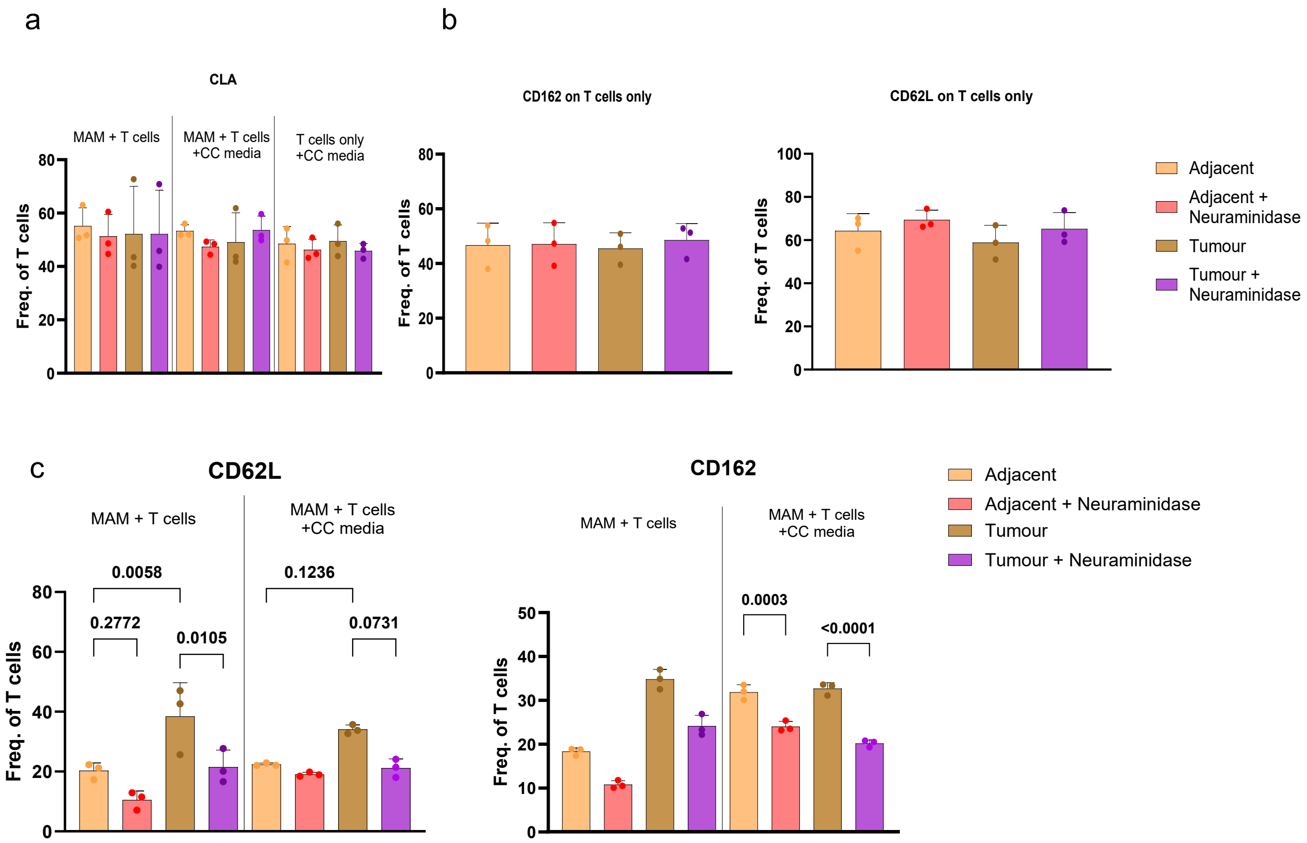

Updated Fig.8 – a-b, Barchart of flow cytometry expression patterns of CLA, CD62L and CD162 on T cells shown as percentage of positive cells from T cell population. Line at mean with standard deviation error bars. Mixed-effects analysis with Tukey's multiple comparisons test. N=3 (N=3 adjacent, N=3 tumor, N=3 blood cones). **c,** Barchart of flow cytometry expression patterns of CD62L and CD162 shown as percentage of positive cells from T cell population. Line at mean with standard deviation error bars. Mixed-effects analysis with Tukey's multiple comparisons test. N=3 (N=3 adjacent, N=3 tumor, N=3 blood cones).

11. Building on the previous comment, the manuscript repeatedly refers to "ECM hyperglycosylation" as a barrier to immune infiltration. Notably the authors mention increased sialylation and fucosylation of N-glycans. However, the nature of the fucosylation, particularly core vs outer branching, and the type of sialylation (α2,3 vs α2,6) are not fully disclosed. This distinction matters, since different Siglec receptors exhibit divergent binding preferences. To strengthen the mechanistic claim, the authors should clarify which sialylation type is predominantly enriched in tumor ECM and, if possible, align this with Siglec-binding specificities. Exploiting both MAL and SNA lectin and linkage specific sialidases could perhaps help clarify this aspect.

Response. We intentionally used a prolonged (24 h) incubation of neuraminidase at a high concentration (0.5 U) to facilitate cleavage across a broad spectrum of sialic acid linkages, thus dissecting the individual contributions of each linkage type was beyond the original scope of this study.

As suggested by the reviewer, we have used SNA lectin binding as a marker of α 2,6-linked sialylation and MAL I as a marker for α 2,3-linked sialylation. α 2,6-linked sialylation is significantly increased in tumour tissue compared to adjacent whereas α 2,3-linked sialylation is not significantly changed between adjacent and tumour. We have also stained following neuraminidase treatment on tumour tissue and found that α 2,6 levels were significantly decreased but α 2,3 were unchanged (Update Fig7). In conclusion, our data suggests that α 2,6-linked sialic acids are predominantly enriched in tumour ECM and we confirm that our neuraminidase cleaves these.

To our knowledge, α 2,6 can bind Siglec-2, -3, -6, -9, -10, -15. We have already shown in Supplementary Fig. 18 that Siglec - 5, -7, -9, -10, -15 do not change on CAR-T with neuraminidase treatment. However, as discussed before, unchanged Siglecs expression on T cells does not, by itself, exclude a role for Siglec-mediated signaling.

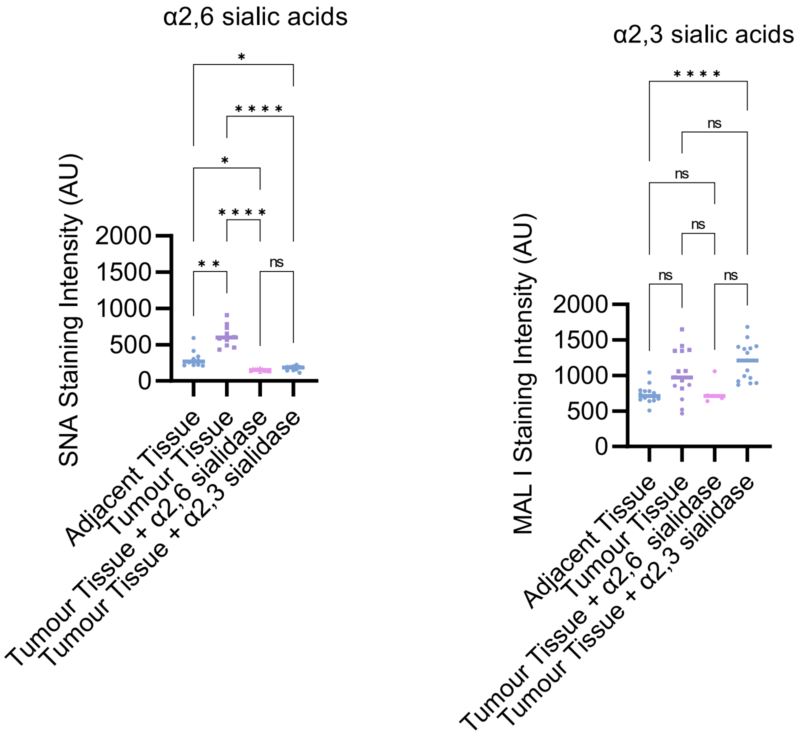

Updated Fig.7 - Analysis of SNA and MAL I lectin staining intensity using IMARIS image analysis software. N=2 matched tissue donors for each group, 5 FOV per tissue, One-way ANOVA with Tukey's post-hoc test.

12. The manuscript also reports increased fucosylation of N-glycans, which is an interesting finding. However, the specific nature of this change remains unresolved, and this distinction could have very different implications at multiple levels. I understand that the MALDI-based workflow provides only compositional information without resolving linkage or positional details, and I acknowledge that the authors have been careful not to overinterpret their data. Nevertheless, only UEA-I was included in the initial lectin panel, which does not address functionally relevant core or Lewis-type fucosylation. Complementary use of more informative probes or MS/MS-based structural characterization would substantially strengthen the mechanistic interpretation.

Response. We appreciate the reviewer's comment and agree in principle that orthogonal quantitative approaches, such as LC-MS/MS with appropriate standards, could provide additional quantitative confidence. However, the scope and practical constraints of this study limit the feasibility of such analyses. This work is an ambitious, multi-modal investigation that integrates several experimental techniques and relies on rare, clinically derived patient tissues that are extremely difficult to obtain. As a result, both sample number and available material were highly limited, necessitating the use of a single MS-based glycomic approach.

While we acknowledge that relative abundance data are compositional in nature, reporting permethylated glycan profiles by MALDI-TOF MS remains a well-established and widely accepted strategy in glycomics for comparative analyses across biological conditions. Moreover, although absolute quantification using glycan standards would indeed be desirable, the availability of comprehensive quantitative standards for the wide diversity of glycans analyzed here is currently very limited. Unlike proteomics (where isotope-labeled peptide standards are relatively straightforward to obtain) glycan synthesis and standardization remain a significant technical challenge in the field.

We therefore believe that the analytical approach used represents a pragmatic and appropriate balance between technical rigor and feasibility, given the complexity of the samples and the exploratory nature of the study, however we do fully agree that the work suggested could be very insightful and something we will look to explore in the future.

13. In the final set of experiments, the authors analyze typical T-cell activation/exhaustion and macrophage lineage/polarization markers after culture on tumor versus adjacent ECM with a with sialidase. While these experiments are very informative, the absence of cytokine profiling limits interpretation of the functional consequences. Assessing them would provide a more complete picture of immune modulation and strengthen the mechanistic link to immune exclusion.

Response. We agree with the reviewer that cytokine profiling provides important functional insight into immune modulation. In response to this comment, we performed cytokine analysis using a LEGENDplex (BioLegend) multiplex assay, assessing eight cytokines in supernatants from MAM-T cell co-cultures cultured on tumor versus adjacent ECM.

We observed that IFN-γ and TNF-α are reduced in the supernatant of MAMs and T cell co-cultures on tumor tissue indicating that tumor ECM influences the co-culture in decreasing immunoinflammatory cytokines release (Updated Fig. 9). Interestingly, neuraminidase treatment on both adjacent and tumor tissues reduced the release of anti-tumoral cytokines, such as IL-6, IL-10, IL-17A and IL-23 (Updated Fig.9), indicating that sialic acid removal decreases pro-tumoral cytokines production, dampening immune suppression, which correlates with the reduction seen in the exhaustion phenotype.

Importantly, cytokines were measured in supernatants collected after 48 h of co-culture. As such, the detected levels likely reflect cumulative secretion over time, potentially integrating early activation and later regulatory phases of the immune response. This temporal integration may mask transient or early differences in cytokine release dynamics. Therefore, we interpret the cytokine data in conjunction with the phenotypic and functional changes observed by flow cytometry at 48 h, which together provide a more comprehensive picture of immune

modulation by tumor ECM sialylation. These data are referred to in page 9 (line 355-366) and the new graph is shown in Fig.7e and Supplementary Fig 24c.

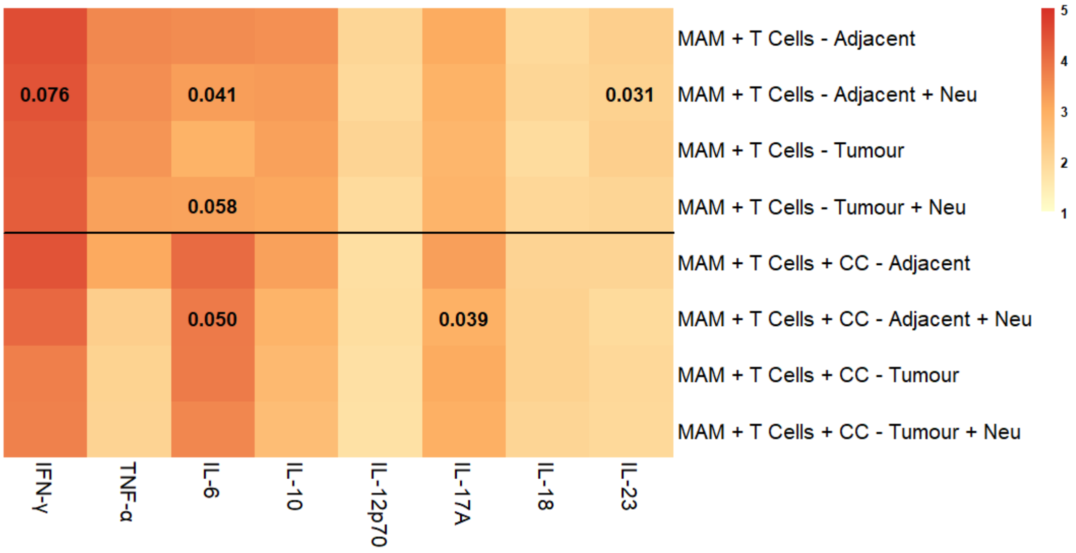

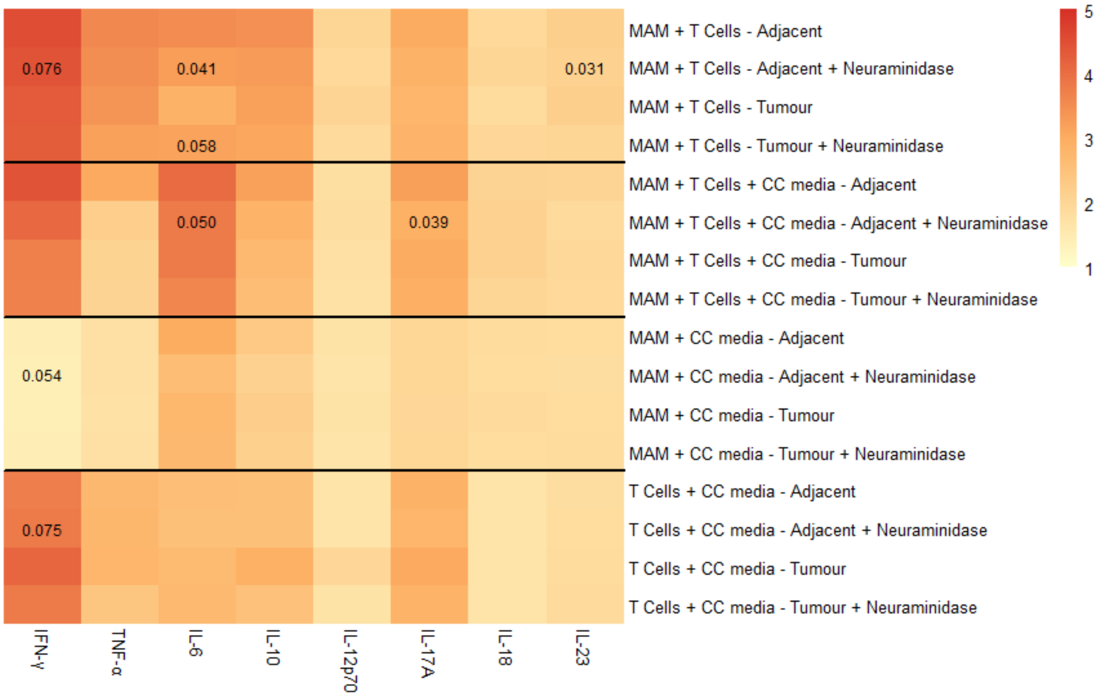

Updated Fig.9 - Heatmaps showing log10-transformed concentrations (pg/mL) of 8 cytokines (IFN-γ, TNF-α, IL-6, IL-10, IL-12p70, IL-17A, IL-18 and IL-23) measured in culture supernatants from macrophage (MAM) and T cell co-cultures (or monoculture) grown on decellularized adjacent or tumor ECM scaffolds, in the absence or presence of cancer cell–conditioned medium (CC). ECM scaffolds were either untreated or treated with neuraminidase (Neu) prior to cell seeding. N=3 (N=3 adjacent, N=3 tumor, N=3 blood cones). Overlaid numbers indicate paired t-test p-values for comparisons between untreated and neuraminidase-treated ECM within the same tissue and CC condition.

14. While not strictly mandatory for the current scope, it would be interesting to address whether tumor ECM exposure also remodels the glycosylation of the immune cells, thereby shaping function.

Response. We agree with the reviewer that tumor ECM-mediated remodeling of immune cell glycosylation represents a highly interesting and potentially important mechanism shaping immune cell function. While addressing this question is beyond the scope of the present study, we view it as a key direction for future work. In ongoing and planned studies, we aim to investigate how tumor-associated cues influence glycosylation programs in both cancer cells and immune cells, and how these changes impact immune regulation within the tumor microenvironment.

15. Regarding the adopted models scope envisaging translational relevance. The central findings rely on an elegant ex vivo platform using decellularized human tumor ECM recellularized with TNBC cell lines and CAR-T surrogates. I recognize the considerable challenges of providing in vivo validation in immunocompetent models. Nevertheless, the translational value of the manuscript would be substantially strengthened if key results were reproduced in well-characterized human tumor/ECM slices with autologous TILs under the glycosidases treatment.

Response. We appreciate the reviewer's recognition of the translational intent of our ex vivo platform and agree that validation in human tumor/ECM slices with autologous tumor-infiltrating lymphocytes (TILs) would further strengthen translational relevance. However, at present, the use of autologous TILs is not technically feasible within our working protocols.

Isolation and expansion of autologous TILs from the patient-derived tissues used here is not possible due to the very limited size and availability of these specimens, which are prioritized for decellularization and ECM analyses. Similarly, although the use of autologous immune cells derived from peripheral blood is an attractive approach, this workflow is not yet established in our laboratory.

We are actively working toward overcoming these limitations, including efforts to establish collaborations with tissue banks that would enable access to matched blood and tissue samples in the future. Development of fully autologous models remains a key long-term goal; however, it currently represents a significant technical and logistical challenge.

We have acknowledged these limitations, butbelieve that the current model nonetheless provides valuable mechanistic insight into how tumor ECM glycosylation modulates immune cell behavior.

Reviewer #3 (Remarks to the Author):

In the present manuscript Tyler et al. investigate the contribution of the glycosylated ECM on T cell infiltration and macrophage phenotype. They present a full characterization of the ECM glycosylation patterns, showing that tumor ECM carries more sialylated structures, resembling the desert phenotype TNBC tumors. This is an elegant study, adding yet another layer to the intricate role glycosylation plays in the tumor microenvironment. I do have a few issues listed below that the researcher should address to further strengthen their results.

Major comments:
- In figure 6 the authors only use the BT20 cell line to assess T cell infiltration and the induction of a desert phenotype. These results need to be confirmed using another cell line. Are similar results obtained in the same model but now using the HCC38 cells?

Response. We agree that it would be interesting to see what the effect on T cells is when co-cultured on top of PNGase F and Neuraminidase treated tissue in presence of a different cancer cell line. For this reason, we followed the reviewer suggestion and repeated the whole experiment with HCC38, another TNBC cell line, which is a bit more mesenchymal-like, compared to BT20. Additionally, we decided for this experiment to use PBMCs-isolated T cells, and subsequently activated, to better recapitulate the human setting.

Similarly to the BT20 experiment already performed for the original version of the manuscript (now moved to Supplementary Fig.13) , we demonstrated that comparing T cells infiltration between tumor-derived ECM and adjacent tissue, T cell infiltration was significantly reduced in tumor-derived ECM (Updated Fig. 3a). Furthermore, fewer T cells penetrated the tumor nest (≤5 μm from a cancer cell) in tumor ECM compared to adjacent tissue (Updated Fig. 3b), resembling an immune-excluded phenotype. In both tumor and adjacent tissues, T cells preferentially accumulated in the stroma (Fig.6b). We have also considered the reviewer comment of combining the graphs showing T cell number near and far from tumor in adjacent and tumor tissue in one graph (Updated Fig.3b).

Analysis of T cell motility revealed that T cells located near a cancer cell (<5μm) have an increased speed and displacement in adjacent tissues compared to tumor tissues (Updated Fig. 3c,d). Moreover, within tumor ECM, T cells that are located near tumor islets exhibited a decreased speed and displacement compared to those in the surrounding ECM (Updated Fig. 3e,f).

We then wanted to test whether enzymatic removal of N-glycans or sialic acids significantly enhanced T cell infiltration into tumor ECM (Updated Fig. 3g) and increased the proportion of T cells penetrating the tumor nest (Updated Fig. 3g,h). Interestingly, T cells in PNGase F or neuraminidase-treated tissues, compared to untreated tumor tissue, exhibited greater and improved speed and displacement when in proximity of a cancer cell (<5μm), indicating that T cells are more easily able to move and reach the tumor islets through the ECM in enzyme-treated tissues (Updated Fig. 3j,k).
These data are referred to in page 5-7 (line 206-253) and the new graph is shown in Fig.5.

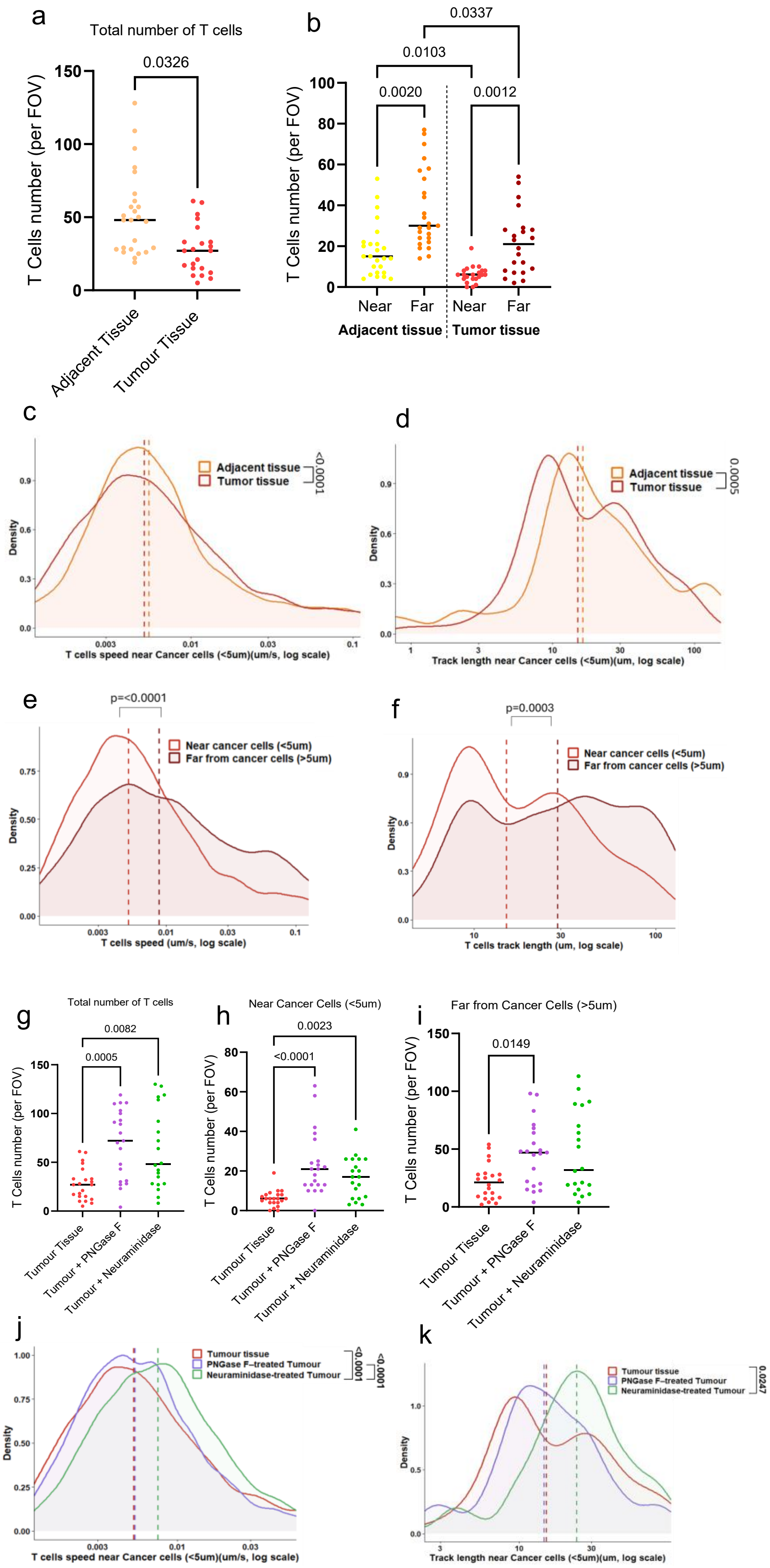

a
Total number of T cells
0.0326
T Cells number (per FOV)
Adjacent Tissue
Tumour Tissue
b
0.0337
0.0103
0.0020
0.0012
T Cells number (per FOV)
Near
Far
Near
Far
Adjacent tissue
Tumor tissue
c
Adjacent tissue
Tumor tissue
<0.0001
Density
T cells speed near Cancer cells (<5um)(um/s, log scale)
d
Adjacent tissue
Tumor tissue
0.0005
Density
Track length near Cancer cells (<5um)(um, log scale)
e
p=<0.0001
Near cancer cells (<5um)
Far from cancer cells (>5um)
Density
T cells speed (um/s, log scale)
f
p=0.0003
Near cancer cells (<5um)
Far from cancer cells (>5um)
Density
T cells track length (um, log scale)
g
Total number of T cells
0.0082
0.0005
T Cells number (per FOV)
Tumour Tissue
Tumour + PNGase F
Tumour + Neuraminidase
h
Near Cancer Cells (<5um)
0.0023
<0.0001
T Cells number (per FOV)
Tumour Tissue
Tumour + PNGase F
Tumour + Neuraminidase
i
Far from Cancer Cells (>5um)
0.0149
T Cells number (per FOV)
Tumour Tissue
Tumour + PNGase F
Tumour + Neuraminidase
j
Tumour tissue
PNGase F-treated Tumour
Neuraminidase-treated Tumour
<0.0001
<0.0001
Density
T cells speed near Cancer cells (<5um)(um/s, log scale)
k
Tumour tissue
PNGase F-treated Tumour
Neuraminidase-treated Tumour
0.0247
Density
Track length near Cancer cells (<5um)(um, log scale)

Updated Fig.3 – a-b, Count of **a,** total T cell number in each field of view (FOV) and **b,** T cell number near (<5µm) and far (>5µm) from a cancer cell in tumor and adjacent tissues recellularized with HCC38 cells. N=3 tissue donors for each group, 5-10 FOV per tissue. Unpaired T-test. **c,** Density distributions of T cell speed near (<5 µm) cancer cells on tumor tissue (red) and adjacent tissue (orange). **d,** Density distributions of T cell track length near (<5 µm) cancer cells on tumor tissue (red) and adjacent tissue (orange). **c-d,** Dashed lines indicate medians. Data presented in log scale. Kruskal-Wallis test with Dunn's multiple comparisons test. N=3, with 2 technical replicates each. **e,** T cell speed (µm/s) near (<5µm) and far (>5µm) from cancer cells in tumor tissue. **f,** T cell track length (µm) near (<5µm) and far (>5µm) from cancer cells in tumor tissue. **e-f,** Line at median. Data presented in log scale. Mann-Whitney test. N=3, with 2 technical replicates each. **g-i,** Count of **g,** total T cell number in each FOV, **h,** T cell number within 5µm of a tumor cell ('near cancer cells') and **i,** T cell number more than 5µm from a cancer cell ('far from cancer cells'). N=3 tissue donors, 6-10 FOV per tissue. One-way ANOVA with Dunnett's multiple comparison's test. **g-i,** Line at median. **j,** Density distributions of T cell speed near (<5µm) cancer cells on tumor tissue (red), tumour + PNGase F treated tissue (violet) or tumour + neuraminidase treated tissue (green). **k,** Density distributions of T cell track length near (<5µm) cancer cells on tumor tissue (red), tumor + PNGase F treated tissue (violet) or tumor + neuraminidase treated tissue (green). **J-k,** Dashed lines indicate medians. Data presented in log scale. Kruskal-Wallis test with Dunn's multiple comparisons test. N=3, with 2 technical replicates each.values +/- standard deviation (SD). Two-way ANOVA with Sidak's multiple comparisons test. N=3.

- For the T cell infiltration experiments the authors fully focus on the sialic acid-Siglec axis, completely ignoring other sialic acid-dependent receptors and adhesion molecules, most importantly the selectins. What is the expression of selectins on the CAR-T cells and are the selectins involved in the phenotype?

Response. We really appreciate the suggestion from the reviewer and decided to explore alternative sialic acid‑dependent pathways and focused on selectins. We observed that CLA, CD62L (L-selectin), and CD162 (PSGL-1) are highly expressed on T cells in our model (Updated Fig. 8a,b). Notably, MAM-educated T cells within tumor tissue exhibited significantly increased CD62L and CD162 expression compared to adjacent tissue (Updated Fig. 8c). This phenotype is consistent with a less cytotoxic and less infiltrative T-cell state, as previously reported. Importantly, neuraminidase treatment of tumor tissues significantly reduced CD62L and CD162 expression, in line with enhanced cytotoxic potential, reduced exhaustion, and improved infiltration capacity of T cells in the tumor microenvironment.

Together, these data suggest that tumor ECM sialylation influences T cell phenotype and function, potentially through multiple sialic acid‑dependent pathways, including selectin-mediated interactions, while Siglec involvement remains an important avenue for future mechanistic studies. These data are referred to in page 9 (line 367-379) and the new graph is shown in Fig.7f and Supplementary Fig 24d,e.

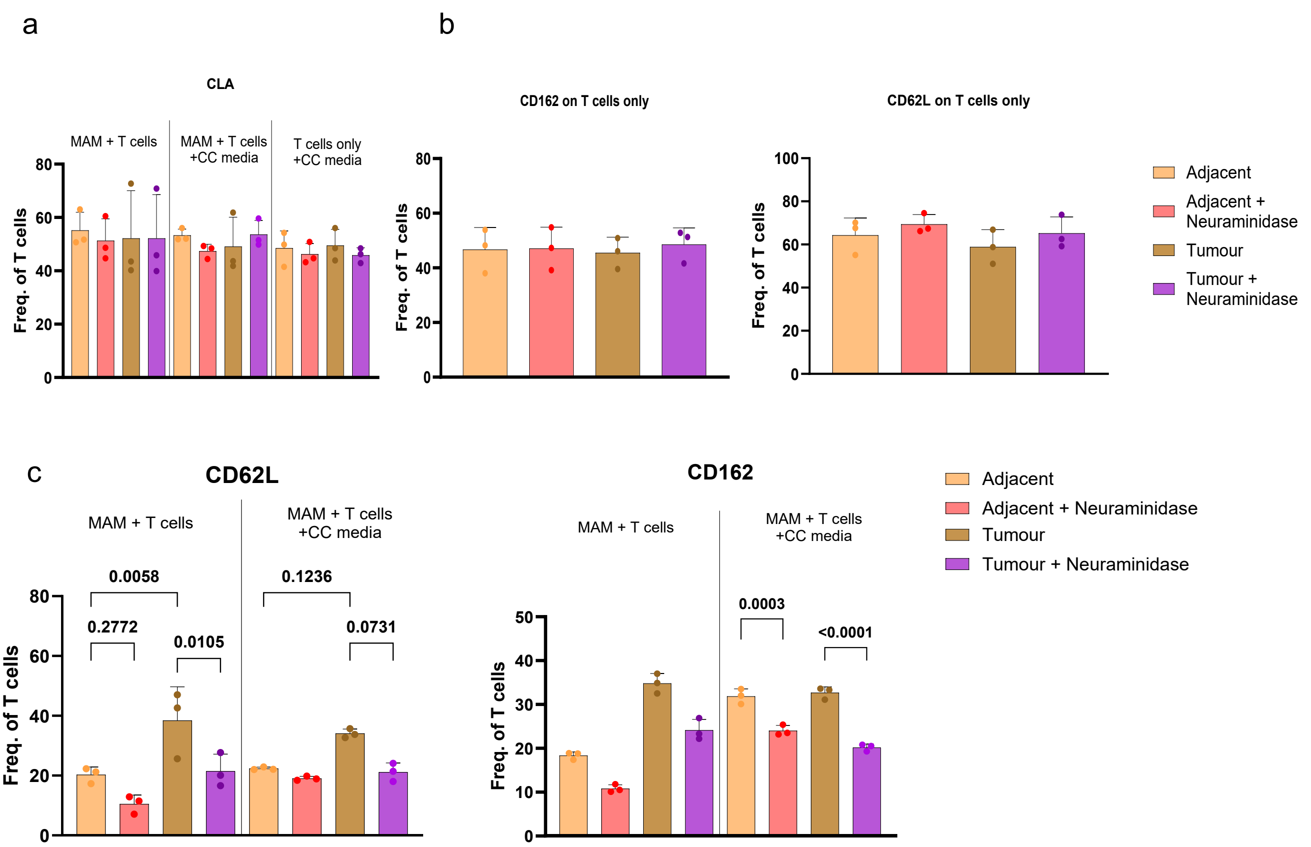

Updated Fig.8 – a-b, Barchart of flow cytometry expression patterns of CLA, CD62L and CD162 on T cells shown as percentage of positive cells from T cell population. Line at mean with standard deviation error bars. Mixed-effects analysis with Tukey's multiple comparisons test. N=3 (N=3 adjacent, N=3 tumor, N=3 blood cones). **c,** Barchart of flow cytometry expression patterns of CD62L and CD162 shown as percentage of positive cells from T cell population. Line at mean with standard deviation error bars. Mixed-effects analysis with Tukey's multiple comparisons test. N=3 (N=3 adjacent, N=3 tumor, N=3 blood cones).

- I understand why the researchers omit the tumor cells and focus only on the macrophages in the experiments depicted in Figs 7 & 8. However, in patients the tumor cells are always present, influencing the macrophages through direct interactions or soluble mediators. Are the effects on macrophage phenotype and subsequent T cells activation still observed when tumor supernatants are mixed in in the co-culture.

Response. We agree with the reviewer that, in patients, cancer cells are always present and influence macrophage phenotype and function through both direct contact and soluble mediators. In response to this comment, we incorporated conditioned media from TNBC cancer cells into the MAM-T cell co-culture system to assess whether tumor-derived soluble factors modulate macrophage education and downstream T-cell responses (updated Fig. 10a).

Specifically, donor-matched T cells were co-cultured with MAMs educated on tumor or adjacent ECM, with or without neuraminidase treatment, and in the presence or absence of cancer cell-conditioned media. This design allowed us to dissect the relative contributions of tumor ECM sialylation and cancer cell-derived soluble factors to macrophage-T cell crosstalk.

Combinatorial analysis of activation markers (CD137 and ICOS) revealed that T cells co-cultured with MAMs educated on tumor ECM exhibited significantly higher activation compared with those co-cultured with MAMs educated on adjacent ECM (updated Fig. 10b). Addition of cancer cell-conditioned media reduced T-cell activation in co-cultures with MAMs educated on adjacent ECM, whereas it did not significantly alter T-cell activation in the tumor ECM condition (updated Fig. 10b). This suggests that tumor ECM imposes a dominant imprint on MAM-driven T-cell activation that is not overridden by cancer cell-derived soluble factors.

Similarly, combinatorial expression of exhaustion markers (PD-1, LAG-3, and TIM-3) was significantly increased on T cells co-cultured with MAMs educated on tumor ECM compared with adjacent ECM (updated Fig. 10c,d). Neuraminidase treatment markedly reduced exhaustion in T cells co-cultured with tumor-educated MAMs, while only modest effects were observed in the adjacent ECM condition (updated Fig. 10c,d). These data support a role for hypersialylated tumor ECM in driving an immunosuppressive macrophage-T cell axis. Notably, cancer cell-conditioned media did not significantly alter exhaustion marker expression in any condition.

Importantly, T cells cultured on decellularized ECM alone, with or without cancer cell-conditioned media, did not recapitulate these phenotypic changes, indicating that the effects on T cells require macrophage-mediated conditioning rather than direct ECM-T cell interactions.

Cytokine profiling further supported these findings: IFN-γ and TNF-α levels were reduced in supernatants from MAM-T cell co-cultures on tumor ECM, consistent with suppressed immunoinflammatory responses (updated Fig. 10e). Neuraminidase treatment reduced the secretion of cytokines associated with pro-tumoral immune regulation (IL-6, IL-10, IL-17A, and IL-23) in both adjacent and tumor ECM conditions, correlating with reduced T-cell exhaustion (updated Fig. 10c-e). Cancer cell-conditioned media did not substantially alter these cytokine trends.

Finally, we assessed whether ECM desialylation influenced selectin-related pathways on T cells. CLA, CD62L (L-selectin), and CD162 (PSGL-1) were highly expressed on T cells in our model. MAM-educated T cells on tumor ECM displayed significantly increased CD62L and CD162 expression compared with adjacent ECM (updated Fig. 10f), consistent with a less cytotoxic and less infiltrative phenotype. Neuraminidase treatment significantly reduced CD62L and CD162 expression in the tumor ECM condition, in line with improved cytotoxic potential and reduced exhaustion. Cancer cell-conditioned media did not significantly affect selectin or selectin ligand expression.

Together, these data indicate that tumor ECM sialylation exerts a dominant influence on macrophage education and downstream T-cell phenotype, which is not overridden by cancer cell-derived soluble factors. This supports a model in which the ECM provides a stable immunomodulatory niche that shapes macrophage-T cell crosstalk in the tumor microenvironment.

These data are referred to in page 8-9 (line 325-379) and the new graph is shown in Fig.7 and Supplementary Fig 24.

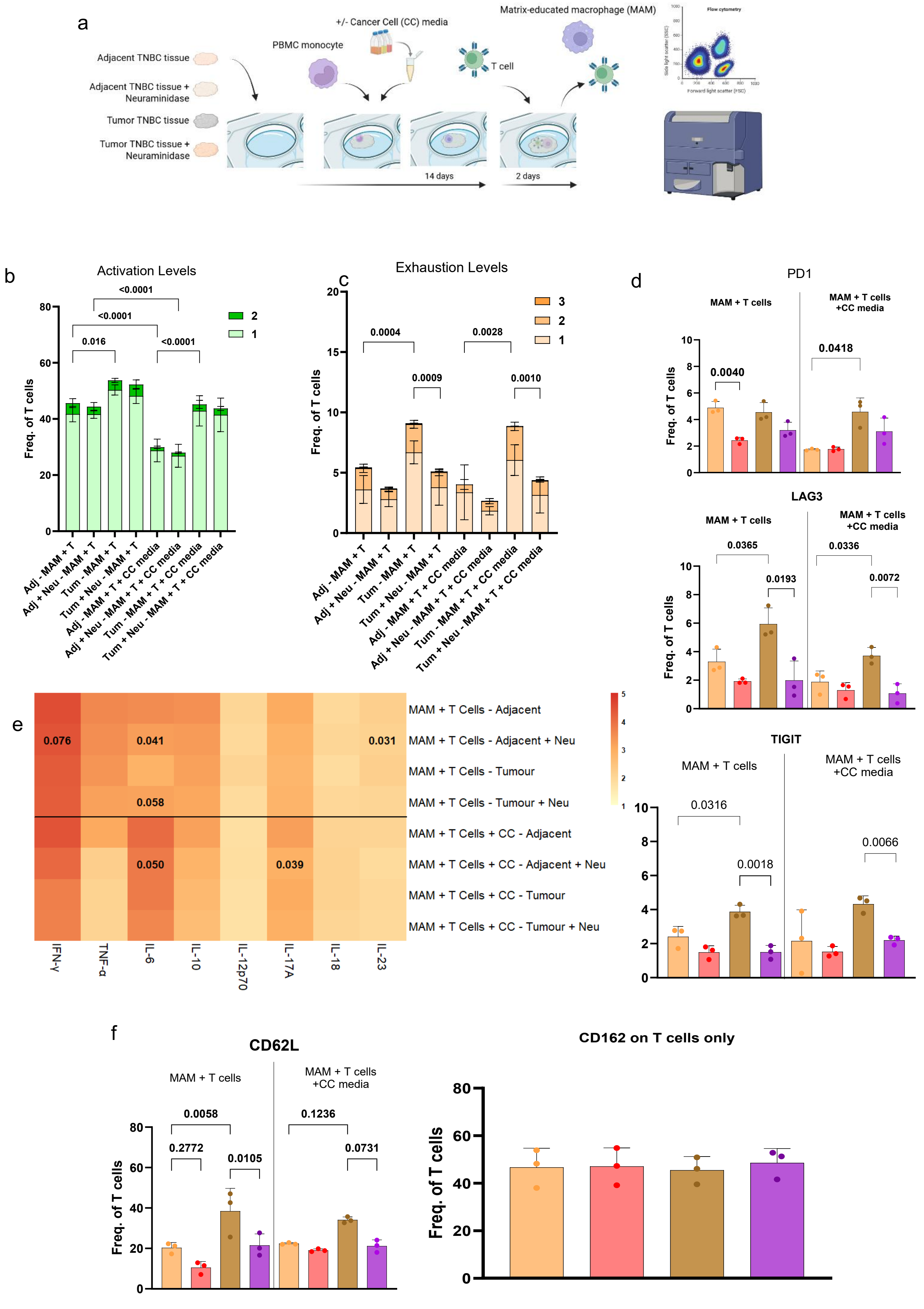

Created in BioRender. Tarantola, L. (2026) https://BioRender.com/ohvhmfy

Updated Fig.8 a, Schematic of macrophage decellularized tissue culture co-cultured with donor-matched T cells, with or without cancer cell (CC) media. Monocytes from three separate blood donors were cultured for 14 days on tumor (N=3) or adjacent (N=3) decellularized tissues treated with neuraminidase (Neu) or sodium acetate vehicle control. Flow gating strategy provided in Supplementary Fig. 23. Created by BioRender. **b-c**, stacked bar of **b**, activation markers (ICOS & CD137) or **c**, exhaustion markers (PD1, LAG3, TIM3) shown as percentage positive cells expressing one marker, two markers or three markers from the T cell population. 2way ANOVA test. N=3 (N=3 adjacent, N=3 tumor, N=3 blood cones). **d**, Barchart of flow cytometry expression patterns of PD1, LAG3 and TIGIT shown as percentage of positive cells from T cell population. Line at mean with standard deviation error bars. Mixed-effects analysis with Tukey's multiple comparisons test. N=3 (N=3 adjacent, N=3 tumor, N=3 blood cones). **e**, Heatmap showing log10-transformed concentrations (pg/mL) of 8 cytokines (IFN-γ, TNF-α, IL-6, IL-10, IL-12p70, IL-17A, IL-18 and IL-23) measured in culture supernatants from macrophage (MAM) and T cell co-cultures grown on decellularized adjacent or tumor ECM scaffolds, in the absence or presence of cancer cell–conditioned medium (CC). ECM scaffolds were either untreated or treated with neuraminidase (Neu) prior to cell seeding. N=3 (N=3 adjacent, N=3 tumor, N=3 blood cones). Overlaid numbers indicate paired t-test p-values for comparisons between untreated and Neu-treated ECM within the same tissue and CC condition. **f**, Barchart of flow cytometry expression patterns of CD62L and CD162 shown as percentage of positive cells from T cell population. Line at mean with standard deviation error bars. Mixed-effects analysis with Tukey's multiple comparisons test. N=3 (N=3 adjacent, N=3 tumor, N=3 blood cones).

- I am not convinced that just by having less CD11b+ macrophages you can draw conclusions about the suppressive nature of these cells. I therefore find the conclusion presented in lines 287-290 a substantial overstatement and I recommend to rephrase these lines.

Response. We understand and on reflection agree that this was an overstatement. We have edited the manuscript with the following text:

"We observed significantly fewer CD11b+ MAMs educated on tumor tissue than adjacent tissue. Given that CD11b has been implicated in regulating myeloid cell polarization and functional states, this shift suggests that tumor ECM exposure may be associated with altered macrophage differentiation or immunomodulatory programs."

However, CD11b expression alone is not sufficient to define suppressive function, and therefore this observation should be interpreted as a phenotypic change consistent with, but not definitive for, an immunoregulatory macrophage state.

This edited text can be found at page 7, lines 295-299.

- Along the same line, the increase in CD163+ macrophages on the neuraminidase treated ECM is explained as beneficial (lines 299-300). Without tumor cells (see above) one cannot conclude this. Actually, in most tumor types CD163 is considered a TAM marker associated with immune tolerance. In the Puttock paper by the same authors (Nat Comm 2023) they perform similar experiments with ECM from high and low disease patients. In this study CD163 is associated

with high disease and a more suppressive T cell phenotype. How do the authors explain their results also in the context of other tumors and their previous paper?

Response. We thank the reviewer for this important point and agree that increased CD163 expression alone cannot be interpreted as a beneficial macrophage phenotype, particularly in the absence of tumor cells. Indeed, in many tumor types, including TNBC, CD163 is widely used as a marker of tumor-associated macrophages with immunoregulatory or tolerogenic features. We therefore agree that our original wording was not clear and have revised this interpretation to avoid framing CD163 upregulation as inherently “beneficial”.

We also acknowledge that in our previous study in high-grade serous ovarian cancer (HGSOC; Puttock et al., Nat Comm 2023), CD163 expression was associated with high disease burden and a more suppressive T-cell phenotype. The apparent discrepancy likely reflects differences in ECM composition and architecture (HGSOC versus TNBC), highlighting that macrophage phenotypes and marker-function relationships are not universally conserved across cancers.

Importantly, in the current study we assessed a limited set of macrophage surface markers (CD163, CD206, CD209), which does not provide a comprehensive view of macrophage functional state. We therefore refrain from assigning a definitive pro- or anti-tumoral identity based on these markers alone and have revised the manuscript accordingly. Future work using transcriptomic profiling (e.g., RNA-seq) will be required to more fully resolve the functional programs induced by ECM sialylation.

Crucially, while neuraminidase treatment of tumor ECM led to increased CD163 and CD209 expression on macrophages, this condition was associated with reduced exhaustion and improved functional profiles in co-cultured T cells. Thus, although the macrophage phenotype inferred from selected surface markers may appear “TAM-like”, the net functional consequence at the level of macrophage-T cell crosstalk is a more anti-tumoral T-cell state. We have clarified this distinction in the revised text, emphasizing that macrophage marker expression does not necessarily predict downstream effects on T cell function in a linear manner.

- In fig. 7b the authors conclude that similar effects are not observed in adjacent ECM, however the same trend is clearly visible. It seems to me a numbers issue. Can the authors include 3 more donors and still draw that same conclusion?

Response. We have added 3 more adjacent donors, and we have seen the same result.

The main change between adjacent and tumor tissue was the downregulation of CD11b expression on tumor tissue (Updated Fig.4).

These data are referred to in page 7 (line 295-299) and the new graph is shown in Supplementary Fig.22a.

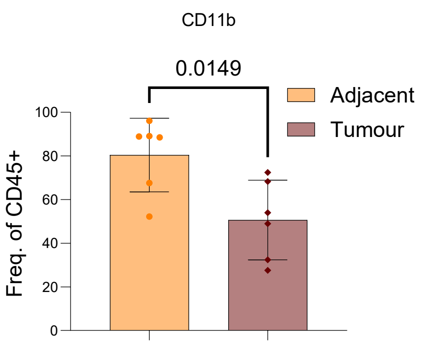

Supplementary Fig.4 Barchart of flow cytometry expression patterns of CD11b shown as percentage of positive cells from the CD45+ population. Line at mean with standard deviation error bars. Mixed-effects analysis with Tukey's multiple comparisons test. N=6 (N=6 adjacent, N=6 tumor, N=6 blood cones).

We then treated tumor ECM with neuraminidase which significantly increased the proportion of CD11b+, CD163+ and CD209+ macrophages (Updated Fig. 11a). This effect was not observed on neuraminidase-treated adjacent ECM, implicating only sialic acids displayed on tumor ECM as modulators of macrophage phenotype. Combinatorial flow analysis of immunomodulatory markers (CD163, CD206, CD209) revealed that the proportion of macrophages co-expressing two immunomodulatory markers was significantly increased on neuraminidase-treated tumor tissue (Updated Fig. 11b), suggesting that sialic acids play a role in immunomodulatory changes of macrophage phenotype in the TME.

These data are referred to in page 7-8 (line 284-316) and part of these new graphs are shown in Fig.6.

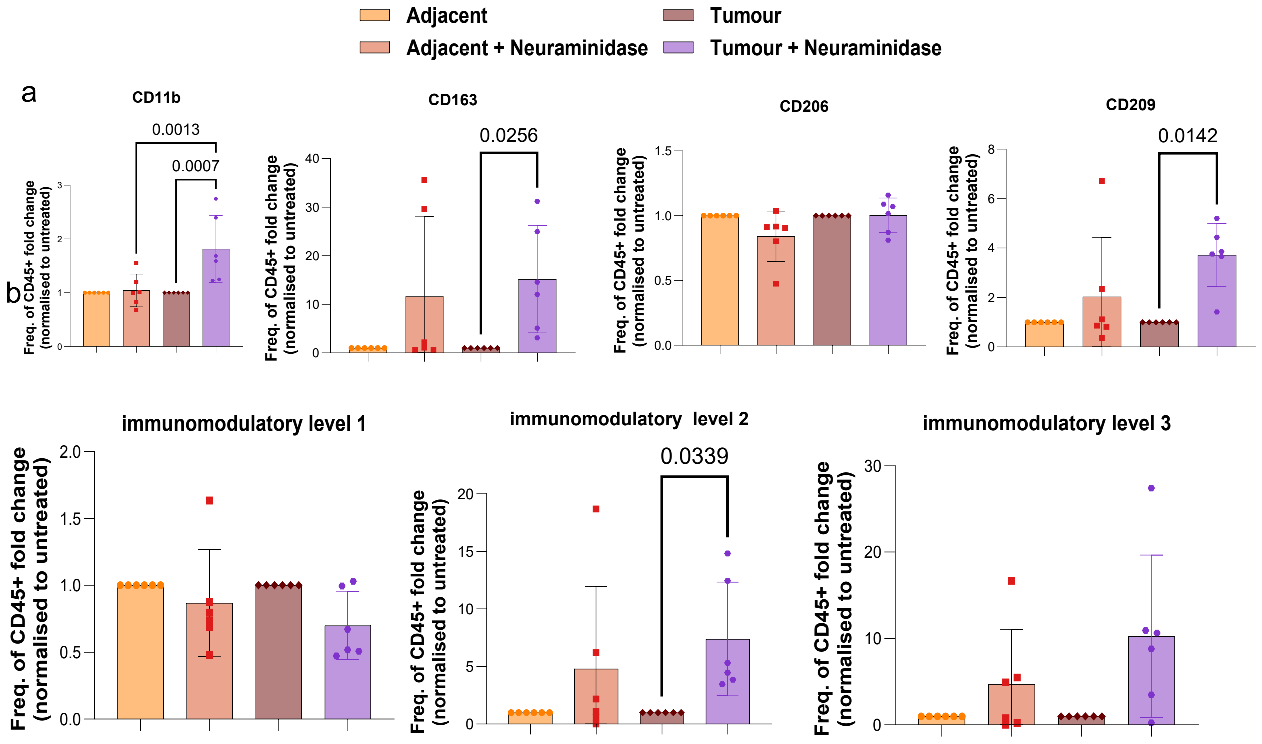

Supplementary Fig.11 a, Barchart of flow cytometry expression patterns of CD11b, CD163, CD206, CD209, shown as fold change normalized to untreated tissue of positive cells from the CD45+ population. Line at mean with standard deviation error bars. Mixed-effects analysis with Tukey's multiple comparisons test. N=6 (N=6 adjacent, N=6 tumor, N=6 blood cones). **b**, Barchart of combinatorial gating for activation markers (CD163, CD206, & CD209) shown as fold change normalized to untreated tissue of positive cells expressing one marker (activation level 1), two markers (activation level 2), or three markers (activation level 3) from the CD45+ population. Line at mean with standard deviation error bars. Mixed-effects analysis with Tukey's multiple comparisons test. N=6 (N=6 adjacent, N=6 tumor, N=3 blood cones).

- Supplementary figure 9 is not mentioned in the main text (maybe Suppl. Fig 10 reference in line 194, should be the suppl fig 9?).

Response. Thank you for spotting this. We have now added the reference to Supplementary Fig.9 in the text. It is now referred to in line 199.

Minor comments:
- Why did the authors co-culture the CAR-T cells first for 1 hour on the ECM before staining the Siglecs? One hour is not enough time for new proteins to come to the cell surface, so this seems like a redundant step to me. Did the authors observe differences in staining the CAR-T directly or after this 1 hour on the ECM?

Response. We have some data on Siglec-9 after 48h and we had the same result. There is no change between conditions and it is also very lowly expressed (around 1.5% of T cells) (Updated Fig. 12)..

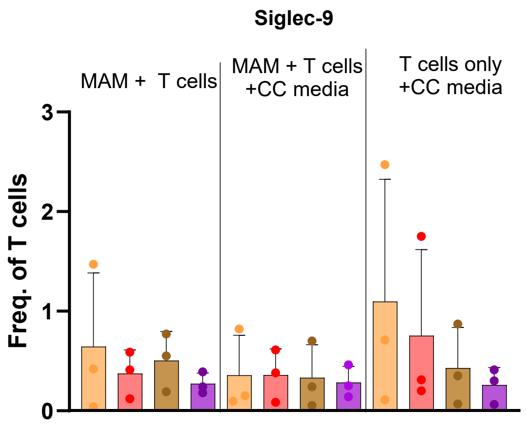

Supplementary Fig.12 a, Barchart of flow cytometry expression patterns of Siglec-9, shown as percentage of positive cells from the T cell population. Line at mean with standard deviation error bars. N=6 (N=6 adjacent, N=6 tumor, N=6 blood cones).

- I would opt to move suppl figures 11C&D to the main figures, as these contain a crucial validation of the ECM components.

- Supplementary methods, line 277: PNA does not stain terminal β-galactose as stated, PNA is really highly specific for the Galβ1-3GalNAc linkage found in O-glycans. This needs correcting.

Response. Thank you for pointing this out. This is now corrected.

- Suppl. Fig 6b: PNA recognizes Gal-GalNAc, not Gal-GlcNAc

Response. Thank you for pointing this out. This is now corrected.

- Also some of the lectin images in the suppl. Figures need correcting:
Suppl. Fig 6A/11A: ConA does not interact with terminal mannoses, but actually binds the internal mannose core (also present in complex N-glycans).
Suppl. Fig 6A: SNA and MAL-I cannot bind the same sialic acid on an N-glycan, as this sialic acid is either α2-3 or α2-6 linked.
Both of these are confusing (and incorrect) and should be changed.

Response. We have updated the images to make them clearer. ConA in our experience and from literature will bind terminal mannose with higher affinity than internal units, so we have not changed the schematic for ConA binding.
- The colors of figures 1g and 2e are difficult to distinguish, so I would suggest to alter the colors to make these figures more readable.

Response. Thank you for pointing this out. We have made this clearer in the figure legend for what each colour represents.

General Response to all reviewers

REVIEWERS' COMMENTS

Reviewer #1 (Remarks to the Author):

I feel the revised manuscript is substantially improved, more robust and focused. All my comments have been addressed and I support its publication.

Reviewer #2 (Remarks to the Author):

The revised manuscript by Tyler, Liu and Tarantola et al. presents a comprehensive and well-executed study addressing the role of ECM glycosylation in shaping the immune microenvironment of TNBC. By combining multi-omics profiling with functional decellularized tissue models, the authors provide compelling evidence that ECM glycan remodeling, particularly increased sialylation, contributes to T cell exclusion, macrophage programming, and macrophage-T cell crosstalk.

The revised version of the manuscript represents a clear improvement over the original submission. The authors have reasonably addressed my concerns and strengthened the study through the inclusion of additional data, improved clarity in data presentation, and a more focused narrative. Overall, I consider the work to be novel, methodologically robust, and of clear relevance to the fields of tumor immunology and glycobiology, with potential translational implications.

That said, a few minor points should be addressed to further strengthen the mechanistic interpretation and overall clarity of the study:

1. Functional data convincingly show that enzymatic removal of glycans enhances T cell infiltration and alters macrophage phenotypes. However, the extent to which these effects are driven by glycan-mediated signaling versus changes in ECM architecture remains to be fully clarified. As glycosidase treatment is likely to impact matrix organization and physical properties, the resulting structural alterations may substantially contribute to the observed phenotypes. Given that this issue is central to the mechanistic interpretation of the study, it would be important to more explicitly acknowledge and discuss this limitation.

We agree and have now addressed and acknowledged the limitation. This can be found in the manuscript at line 283-289: “Notably, removing glycans from the ECM leads to a reorganization of fibre architecture. Although this phenomenon was not explored further here, it may arise from altered glycan–glycan or glycan–protein interactions between ECM fibres, and/or from the release of ECM-associated factors that are normally retained through glycan-mediated binding. Such structural changes are likely to influence T-cell infiltration by jointly affecting glycan-dependent signalling, matrix organization, and the physical properties of the tissue.”

2. The classification of tumors into inflamed, excluded, and desert phenotypes provides a valuable framework. However, its integration with ECM glycosylation features and matrix-related signatures across the study remains somewhat limited. I appreciate that the authors

have reasonably highlighted constraints related to model systems and intratumoral heterogeneity. Still, a clearer discussion of these limitations and their implications for translational interpretation would further improve the overall coherence of the manuscript.

We addressed this point in the discussion in line: 495-503. “Importantly, a related limitation of our study is the integration of immune phenotypes (inflamed, excluded, and desert) with ECM glycosylation features, as intratumoral heterogeneity constrains direct mapping in the decellularised model. Consistent with our previous analysis (Hirani et al.), 69% of TNBC tissues contained mixed immune phenotypes, which may be reflected in the decellularised models made from tissue. Therefore the ECM features and immune behaviours observed likely reflect localized microenvironments specific to each model, which should be considered in translational interpretation and addressed in future spatially resolved studies”.

In summary, this is a strong and timely study that provides important insight into how ECM glycosylation contributes to immune regulation in TNBC. The remaining points relate primarily to interpretation and framing.

Reviewer #3 (Remarks to the Author):

I thank the authors for their elaborative effort and the additonal experiments performed according to my suggestions. In my opinion this has significantly improved the manuscript.
